# Vibralactone derivatives isolated from co-cultures of the basidiomycetes *Stereum hirsutum* and *Boreostereum vibrans*

**DOI:** 10.1007/s13659-025-00505-y

**Published:** 2025-03-31

**Authors:** Jinjuan Wei, Zhe-Xi Li, Gao-Ke Peng, Xinyang Li, He-Ping Chen, Ji-Kai Liu

**Affiliations:** School of Pharmaceutical Sciences, South-Central Minzu University, Wuhan, 430074 China

**Keywords:** *Stereum hirsutum*, *Boreostereum vibrans*, Basidiomycetes, Co-culture, Vibralactone derivatives

## Abstract

The basidiomycetes *Stereum hirsutum* and *Boreostereum vibrans* are two fungi of the same genus. In this study, chemical investigation on the co-cultures of the two congeneric fungi led to the isolation of eleven new vibralactone derivatives, hirsutavibrins A–K (**1**–**11**). The structures of **1**–**11** were elucidated by extensive NMR and HRESIMS spectroscopic analysis, and computational methods. Hirsutavibrins A (**1**) and B (**2**) showed weak cytotoxicity against the human lung cancer cell line A549. Hirsutavibrin D (**4**) showed moderate anti-nitric oxide activity in murine monocytic RAW 264.7 macrophages. This work not only expands the members of vibralactone derivatives with variable configurations but also opens a new avenue for fungal co-culturing study between congeneric fungi.

**Graphical Abstract:**

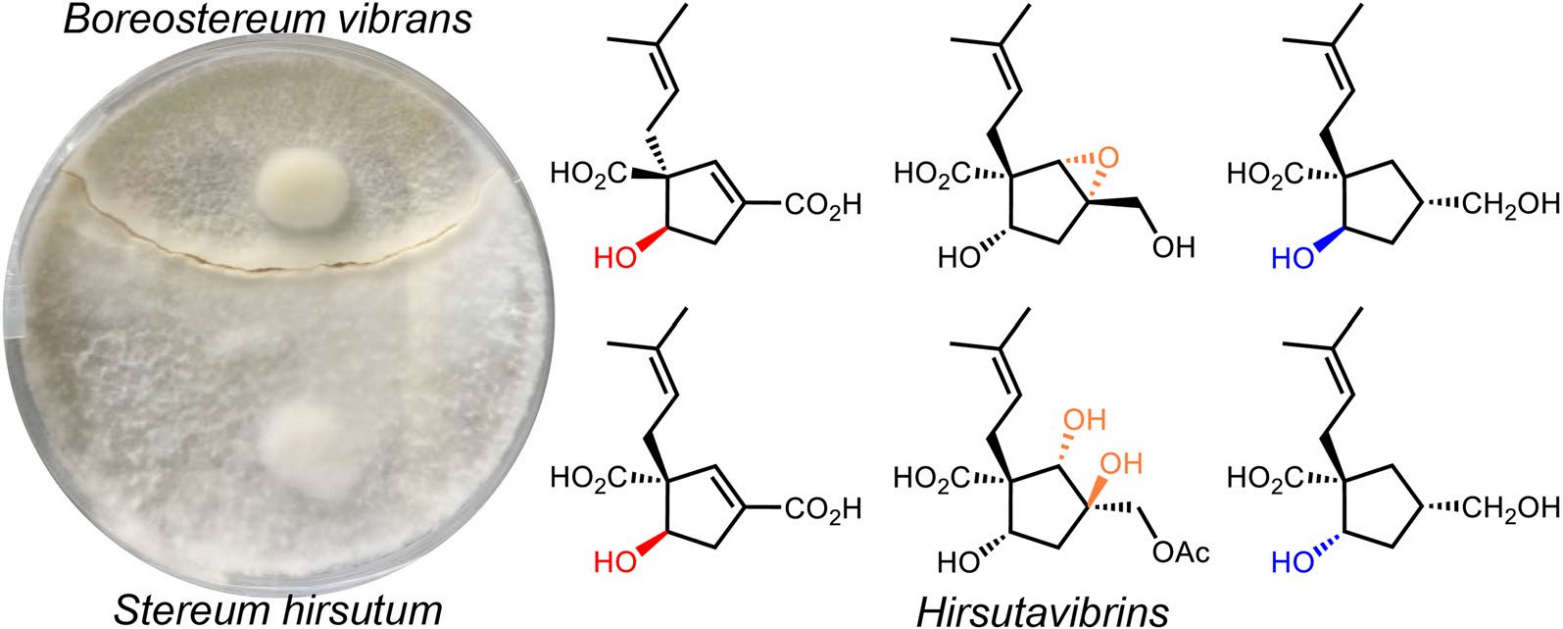

**Supplementary Information:**

The online version contains supplementary material available at 10.1007/s13659-025-00505-y.

## Introduction

Natural products are a vital reservoir of compounds with intriguing structures and diverse bioactivity [1–6]. Microbes are particularly advantageous for natural product discovery due to their easy accessibility and amenability to genetic manipulation [7]. Secondary metabolites of fungal origin represent a crucial subgroup of natural products, exhibiting structural and biological diversity distinct from those found in plants and animals [8]. Several effective strategies are commonly employed to fully explore and expand the chemodiversity of microbial natural products. These include culturing in the presence of epigenetic modifiers [9] or in large-scale [10], co-culturing fungi with other fungi or bacteria [11], overexpressing key transcription factors within gene clusters of interest [12], and heterologous expression of biosynthetic gene clusters [13].

Our research group has long been dedicated to the chemical and biological study of specialized metabolites from higher fungi [14–18]. In previous work, vibralactone, a bicyclic β-lactone-containing meroterpene isolated from *Boreostereum vibrans*, a basidiomycetous fungus [19], was found to exhibit significant pancreatic lipase inhibitory activity (IC_50_ 0.4 μg/mL). Subsequent studies on the cultures of *B. vibrans* resulted in the isolation of numerous vibralactone derivatives [20–23]. Both the total synthesis and biosynthesis of vibralactone have been successfully achieved [24–28]. Beyond our work with *B. vibrans*, we also focus on the chemodiversity of *Stereum hirsutum* HFG27, the type strain of the genus *Stereum*. *Stereum hirsutum* is a prolific producer of meroterpenoids [29], steroids [30], and sesquiterpenoids [31], and has also been found to produce trace amount of vibralactone (about 100 μg/L). Genetic analysis of these two fungi revealed that they both harbor the key biosynthetic genes for vibralactone and extensive homologous genes [26], but they also display distinct biosynthetic potential for other types of natural products.

Co-culturing microorganisms often involves combining two phylogenetically distinct fungi or a fungus with a bacterium to significantly stimulate the production of secondary metabolites during microbial interactions [32]. While only a few studies have focused on the co-culture of two pairs of basidiomycetous fungi from different families, such as co-culture *Trametes versicolor* with *Ganoderma applanatum*, *Pleurotus ostreatus* with *Trametes robiniophila*. As a result, eleven compounds, including phenolic glycosides and terpenoids, have been reported in total [33–35]. *Boreostereum vibrans* and *Stereum hirsutum*, two closely related fungal species with great potential of producing secondary metabolites, were co-cultured. Mixed cultures of these two fungi may increase the probability of producing a diverse range of compounds, potentially influenced by homologous biosynthetic enzymes. In this study, these two fungi were co-cultured to investigate their chemical profiles. This work details the isolation, structural characterization, and biological assessment of the resulting compounds.

## Results and discussion

### Structural elucidation of the previously undescribed compounds

**Fig. 1:**
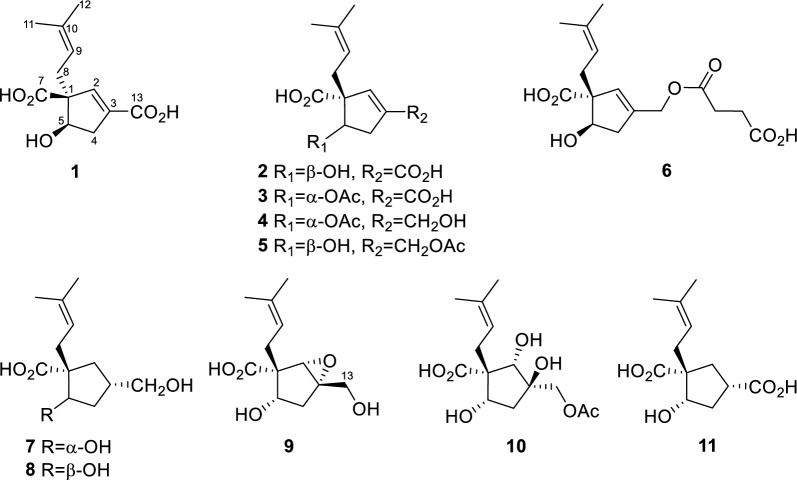
Chemical structures of compounds **1**–**11**

**Fig. 2:**
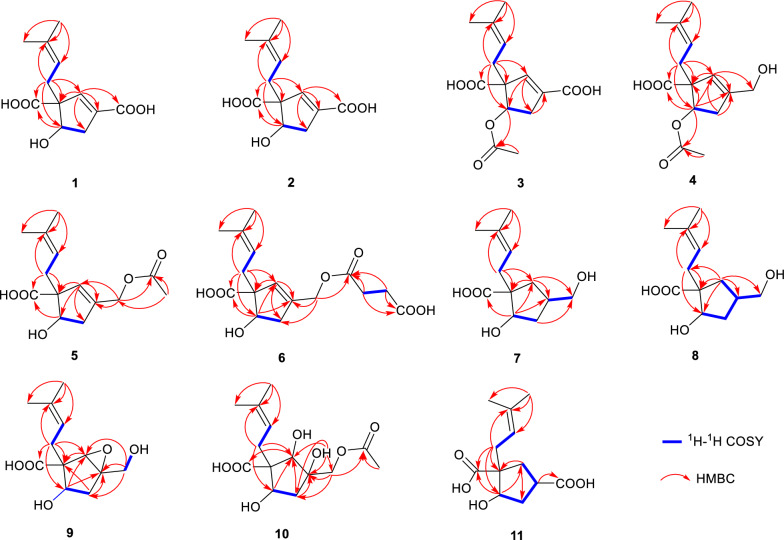
Key ^1^H-^1^H COSY and HMBC correlations of compounds **1**–**11**

Hirsutavibrin A (**1**, Fig. 1) was isolated as a colorless oil. High-resolution positive electrospray ionization mass spectrometric [( +)-HRESIMS] analysis of **1** returned a protonated ion peak at *m/z* 241.10703 [M + H]^+^, corresponding to the molecular formula of C_12_H_16_O_5_ (mass error 0.08201 ppm) with five degrees of unsaturation. The ^1^H NMR spectrum of **1** (Table 1) revealed two olefinic protons at *δ*_H_ 6.72 (1H, s) and 5.12 (1H, t, *J* = 7.6 Hz), in addition to two methyl groups at *δ*_H_ 1.70 (3H, s) and 1.61 (3H, s). The ^13^C NMR (Table 2) and Distortionless Enhancement by Polarization Transfer (DEPT) spectroscopic data of **1** presented 12 carbon signals, including two CH_3_ at *δ*_C_ 18.2 (C-11) and 26.3 (C-12), two CH_2_ at *δ*_C_ 36.0 (C-8) and 40.8 (C-4), three methine carbons at *δ*_C_ 78.4 (C-5), 145.7 (C-2), and 120.6 (C-9), three proton-deficiency carbons at *δ*_C_ 67.4 (C-1), 135.9 (C-10), and 136.6 (C-3), and two carbonyl groups at *δ*_C_ 177.3 (C-7) and 169.1 (C-13). The ^1^H-^1^H COSY correlations of H-4/H-5, along with the HMBC correlations from H-2 to C-1, C-3, C-4, C-5, C-13 (Fig. 2) revealed the existence of a five-membered ring in **1**, which, together with the two carbonyl groups and two double bonds mentioned above, satisfied the unsaturation degree of compound **1**. The ^1^H–^1^H COSY correlation of H_2_-8/H-9, and the HMBC correlations from H_3_-12 to C-9, C-10, C-11, and from H-8 to C-1 indicated a prenyl group attach to C-1. In addition, the HMBC correlations from H-2 to C-13, from H-8 and H-5 to C-7 enabled the assignment of two carboxylic groups at C-1 and C-3, respectively. Therefore, the planar structure of **1** was established (Fig. 1), which highly resembled vibralactone E [21], except that the CH_2_OH group (C-13) was replaced by a COOH group in **1**.

**Table 1:** ^1^H NMR spectroscopic data of compounds **1**–**6**

No	**1**^ac^	**2**^ad^	**3**^ac^	**4**^ac^	**5**^bc^	**6**^ac^
2	6.72, s	6.62, s	6.73, s	5.72, s	5.70, s	5.66, s
4	2.87, dd (16.7, 5.8) 2.53, dd (16.7, 2.4)	2.88, dd (16.3, 7.1) 2.46, dd (16.3, 6.2)	2.94, dd (17.5, 4.7) 2.52, d (17.5)	2.78, dd (17.1, 6.1) 2.33, dd (17.1, 2.1)	2.67, dd (16.1, 7.1) 2.34, dd (16.1, 6.7)	2.66, dd (16.0, 7.1) 2.30, dd (16.0, 5.4)
5	4.22, dd (5.8, 2.4)	4.59, dd (7.1, 6.2)	5.27, d (4.7)	5.27, dd (6.1, 2.1)	4.66, dd (7.1, 6.7)	4.57, dd (7.1, 5.4)
8	2.56, dd (14.0, 7.6) 2.30, dd (14.0, 7.6)	2.60, dd (14.2, 7.6) 2.41, dd (14.2, 7.6)	2.60, dd (13.8, 7.3) 2.35, dd (13.8, 7.3)	2.58, dd (13.9, 7.2) 2.29, dd (13.9, 7.2)	2.53, dd (14.1, 7.2) 2.42, dd (14.1, 7.2)	2.53, dd (14.0, 6.8) 2.35, dd (14.0, 8.3)
9	5.12, t (7.6)	5.20, t (7.6)	5.12, t (7.3)	5.10, t (7.2)	5.14, t (7.2)	5.18, t-like (7.4)
11	1.61, s	1.61, s	1.60, s	1.61, s	1.60, s	1.61, s
12	1.70, s	1.70, s	1.69, s	1.69, s	1.69, s	1.69, s
13				4.11, s	4.59, s	4.61, d (14.1) 4.64, d (14.1)
2'						2.58, overlapped
3'						2.62, overlapped
5-OCOCH_3_			1.98, s	1.97, s		
13-OCOCH_3_					2.08, s	

^a^Measured in CD_3_OD;

^b^Measured in CDCl_3_;

^c^Measured at 600 MHz;

^d^Measured at 500 MHz

**Fig. 3:**
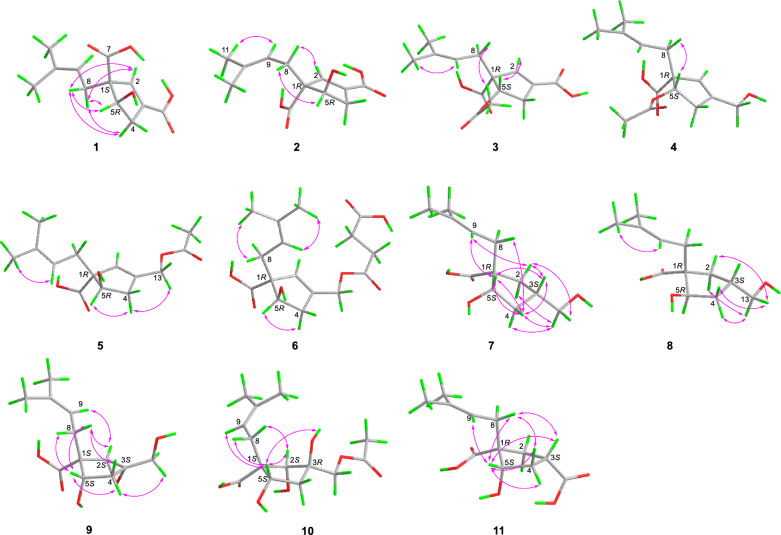
Key ROESY correlations of compounds **1**–**11**

**Fig. 4:**
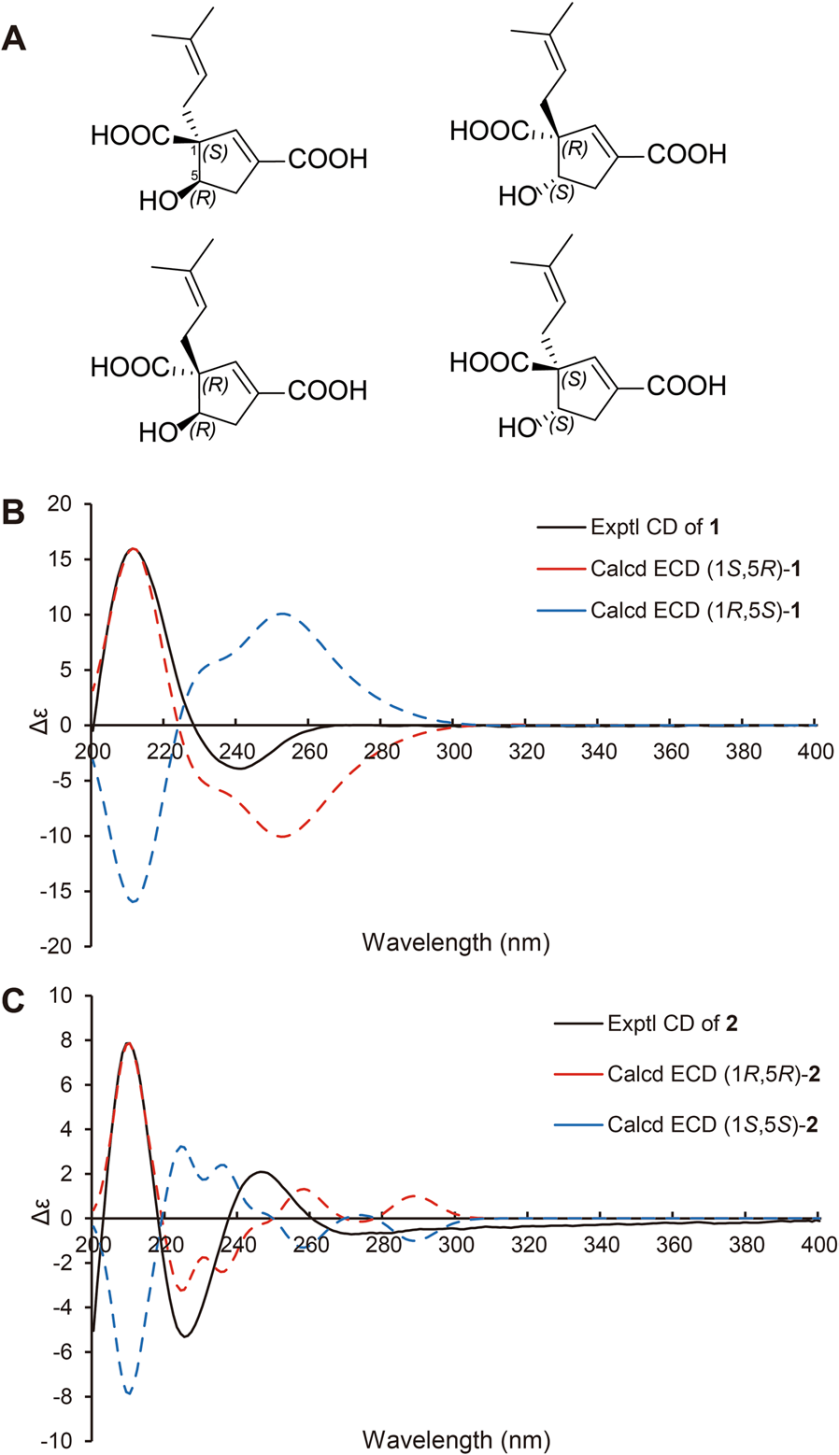
**A** The four possible stereoisomers of **1** and **2**. **B** Comparisons of the experimental CD and calculated ECD of **1**. **C** Comparisons of the experimental CD and calculated ECD of **2**

The conclusion of the relative configuration of **1** arrived by analyzing the ROESY spectrum (Fig. 3). The key correlations of H-5 (*δ*_H_ 4.22)/H-8a (*δ*_H_ 2.30), H-5/H-4α (*δ*_H_ 2.87), and H-8a/H-4α helped to locate H-5 and the prenyl group orienting the same side of the five-membered ring. Besides, almost all the prenyl group at C-1 from the previously reported vibralactone and its derivatives are β configuration, thus the relative configuration of **1** was determined to be 1R*,5S*. The absolute configuration of **1** was determined by ECD calculations (Supplementary Material, Fig. 4). As a result, the calculated ECD of the stereoisomer 1*S*,5*R* was most consistent with the experimental CD data of **1**, while the calculated ECD data of 1*R*,5*S* stereoisomer showed mirror Cotton effects at specific wavelengths (Fig. 4B). Therefore, the absolute configuration of **1** was assigned as depicted in Fig. 1, which was different from the previously reported vibralactone derivatives.

Hirsutavibrin B (**2**, Fig. 1) was obtained as a colorless oil. Its molecular formula was determined to be C_12_H_16_O_5_ based on (+)-HRESIMS, which presented an [M + H]^+^ ion peak at *m/z* 241.10707 (calcd for C_12_H_17_O_5_, 241.10705). The ^13^C NMR and DEPT spectroscopic data of **2** (Table 2) showed two CH_3_ at *δ*_C_ 18.0 and 26.1, two CH_2_ at *δ*_C_ 30.9 and 39.9, three CH at *δ*_C_ 76.9, 145.1, and 120.7, three non-protonated carbons at *δ*_C_ 64.6, 135.5, and 135.4, and two carbonyl carbons at *δ*_C_ 178.0 and 168.5. The 1D NMR data showed high similarity with those of **1**. By interpretating of the 2D NMR spectra, including HSQC, ^1^H–^1^H COSY, and HMBC spectra (Fig. 2), **2** was determined to have a planar structure identical to **1**. The weak correlation signal of H-5 (*δ*_H_ 4.59)/H-8a (*δ*_H_ 2.60) in the ROESY spectrum indicating that H-5 and the prenyl group resided the opposite sides of the five-membered ring, which helped to establish the relative configuration of **2** as 1R*,5R*. An ECD calculation workflow was applied to determine the absolute configuration of **2**. As shown in Fig. 4C, the calculated ECD of the 1*R*,5*R* stereoisomer matched well with the experimental CD data. Therefore, the evidence allowed the complete assignment of 2D structure and absolute configuration of **2** (Fig. 1).

**Fig. 5:**
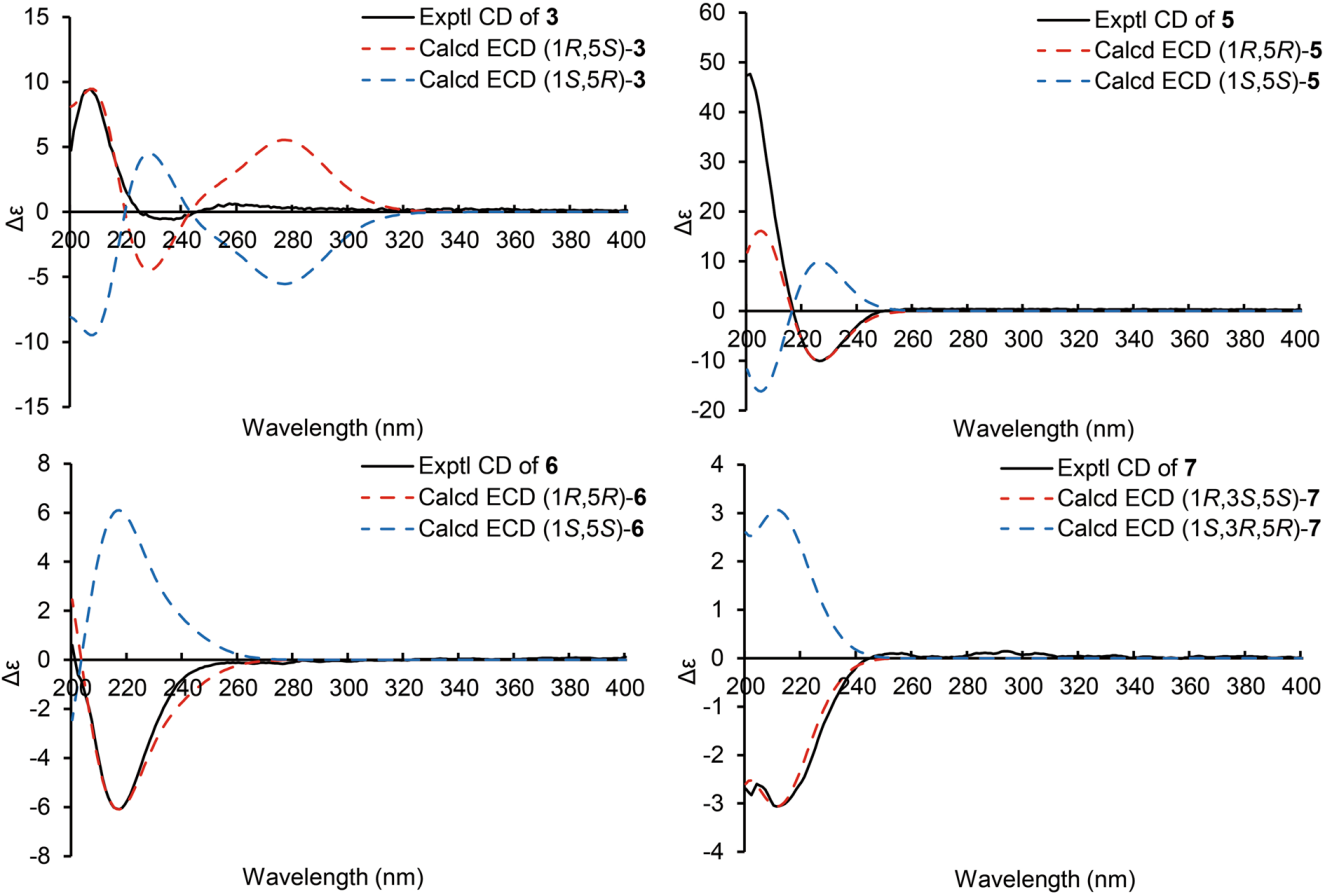
ECD calculations of **3**, **5**–**7**

Hirsutavibrin C (**3**, Fig. 1) was obtained as a colorless oil. The molecular formula of **3** was determined as C_14_H_18_O_6_ according to the HRESIMS data (*m/z* 283.11765 [M + H]^+^, calcd for C_14_H_19_O_6_, 283.11761), indicating six degrees of unsaturation. The ^1^H and ^13^C NMR data of **3** (Tables 1 and 2) were closely similar to the data of **2**, indicating the structure of **3** closely resembled that of **2**, with the primary difference being associated with an extra acetoxyl group in **3**. The methyl singlet at *δ*_H_ 1.98, the carbonyl carbon at *δ*_C_ 172.1, together with the key HMBC correlation from H-5 (*δ*_H_ 5.27) to *δ*_C_ 172.1 (Fig. 2) of **3** enabled the assignment of the acetoxyl group attaching to C-5. Although in the ^13^C NMR spectrum, the signal for C-13 was not detected, which is common for carboxylic groups [36], the HRESIMS result and the chemical shifts of C-2, C-3 and C-4 confirmed that a carboxylic acid group attached to C-3. The crucial and significant correlation of H-5/H-8 (*δ*_H_ 2.60, 2.35) in the ROESY spectrum (Fig. 3) assigned the H-5 and the prenyl group locating at the same side of the five-membered ring (Fig. 2). In addition, the calculated ECD spectrum of (1*R*,5*S*)-**3** showed similar adsorption trend with the experimental CD spectrum of **3**. Therefore, the absolute configuration of **3** was established as 1*R*,5*S* (Figs. 1 and 5).

The colorless oil hirsutavibrin D (**4**, Fig. 1) had a molecular formula of C_14_H_20_O_5_ which returned from the (+)-HRESIMS analysis (*m/z* 269.13834 [M + H]^+^), revealing five degrees of unsaturation. In the ^13^C NMR spectrum of **4**, 14 carbon signals were displayed (Table 2), which showed similarity to **3**, except that an oxygenated methylene (C-13, *δ*_C_ 61.6) in **4** replaced the carbonyl group signal of **3**. The key HMBC correlations from H-2 (*δ*_H_ 5.72) and H-13 (*δ*_H_ 4.11) to C-3 (*δ*_C_ 144.4), and from H-2 to C-13 (Fig. 2) supported the conclusion that the C-13 of **4** was a hydroxymethyl group instead of being a carboxylic acid group as in **3**. The key ROESY correlation between H-5 and H-8 (Fig. 3) suggested that the prenyl group and H-5 were in the same plane of the five-membered ring. Therefore, compound **4** was identified as a C-13 reduced product of **3**.

The molecular formula of hirsutavibrin E (**5**, Fig. 1) was determined as C_14_H_20_O_5_ according to the (+)-HRESIMS data (*m/z* 269.13834 [M + H]^+^, calcd. for C_14_H_21_O_5_). The 1D NMR spectroscopic data suggested that **5** (Table 2) was a congener of **4**, except that the acetoxyl group was changed to locate at C-13 according to the ^3^*J*-HMBC correlations from the proton at *δ*_H_ 4.59 (H-13) to *δ*_C_ 170.9, and the ^4^*J*-HMBC correlations from methyl at *δ*_H_ 2.08 (13-OCOCH_3_) to C-13 (*δ*_C_ 62.8) (Fig. 2). The key correlation between H-5 and H-8 (Fig. 3) was absent in the ROESY spectrum, while between H-5 and H-4α was seen. Therefore, the relative configuration of **5** was speculated to be 1R*,5R*. The calculated ECD curve of (1*R*,5*R*)-**5** showed a Cotton effect similar to the experimental one (Fig. 5), thus establishing the absolute configuration of **5** to be 1*R*,5*R*.

Compound **6** (Fig. 1) was isolated as a colorless oil. The molecular formula was assigned as C_16_H_22_O_7_ based on the positive HRESIMS ion peak at *m/z* 327.14359 [M + H]^+^ (calcd for C_16_H_23_O_7_, 327.14383). Compared to the NMR data of **5** (Tables 1 and 2), the signals for acetyl group were absent in compound **6**. In addition, four additional carbon resonances which were ascribable to two carbonyl groups (*δ*_C_ 174.1, 176.7) and two methylenes (*δ*_C_ 30.2, 30.3) (Table 2) were presented in **6**. By interpreting the 2D NMR spectra, the four carbons were assigned to be a succinic acid moiety, which was determined by the correlations from H-2’ (*δ*_H_ 2.58) and H-3’ (*δ*_H_ 2.62) to C-1’ (*δ*_C_ 174.1) and C-4’ (*δ*_C_ 176.7), from H-13 (*δ*_H_ 4.61) to C-1’ and C-3 (*δ*_C_ 138.3) (Fig. 2) in the HMBC spectrum. The correlation between H-5 and H-8 was not seen in the ROESY spectrum, suggesting a 1R*,5R* configuration of **6**. With the help of ECD calculations, an absolute configuration of 1*R*,5*R* was assigned to **6** based on the highly similar Cotton effect between the experimental CD diagram and the calculated ECD data of (1*R*,5*R*)-**6** (Fig. 5). Therefore, compound **6** was trivially named hirsutavibrin F.

The ^1^H and ^13^C NMR data of **7** (Tables 2 and 3) displayed twelve carbon resonances which were classified into two CH_3_, four CH_2_ (one oxygenated), two *sp*^3^ CH (one oxygenated), one *sp*^2^ CH, one *sp*^3^ qC, and two *sp*^2^ qC (one carbonyl and one olefinic carbon). The NMR data of **7** displayed similarity to those of vibralactone E, except that the C-2–C-3 double bond was reduced to a single bond, which was corroborated by the key ^1^H-^1^H COSY correlations between H-2 (*δ*_H_ 2.31) and H-3 (*δ*_H_ 2.21) (Fig. 2). These assignments reached the conclusion that the molecular formula of **7** was C_12_H_20_O_4_, which was consistent with the HRESIMS analysis results (*m/z* 229.14349 [M + H]^+^, calcd. for C_12_H_21_O_4_, 229.14344). The key correlations of H-2β/H-3/H-4β/H-5 (*δ*_H_ 4.27)/H-8a (*δ*_H_ 2.51), H-2α/H-13/H-4α, and H-5/H-9 in the ROESY spectrum (Fig. 3) indicated the relative configurations of **7** to be 1R*,3S*,5S*. The ECD calculation steps were applied to deduce the absolute stereochemistry of **7**. Comparative analysis of the measured and theoretically calculated ECD curves (Fig. 5) revealed a 1*R*,3*S*,5*S* configuration of **7**. Compound **7** was named hirsutavibrin G.

**Table 2:** ^13^C NMR spectroscopic data of compounds **1**–**11**

No	**1**^ae^	**2**^af^	**3**^ae^	**4**^ae^	**5**^be^	**6**^ae^	**7**^ae^	**8**^ae^	**9**^cf^	**10**^ce^	**11**^af^
1	67.4, C	64.6, C	66.8, C	65.2, C	61.9, C	63.7, C	60.5, C	59.9, C	59.7, C	54.3, C	60.8, C
2	145.7, CH	145.1, CH	144.3, CH	127.7, CH	128.6, CH	131.2, CH	36.2, CH_2_	37.1, CH_2_	63.9, CH	83.7, CH	34.8, CH_2_
3	136.6, C	135.4, C	137.3, C	144.4, C	138.0, C	138.3, C	39.0, CH	38.5, CH	66.8, C	76.9, C	41.6, CH
4	40.8, CH_2_	39.9, CH_2_	39.4, CH_2_	39.7, CH_2_	39.5, CH_2_	41.1, CH_2_	38.0, CH_2_	35.2, CH_2_	36.1, CH_2_	45.0, CH_2_	37.4, CH_2_
5	78.4, CH	76.9, CH	80.2, CH	80.3, CH	76.1, CH	77.1, CH	77.6, CH	78.3, CH	75.0, CH	70.6, CH	79.7, CH
7	177.3, C	178.0, C	176.6, C	177.4, C	180.9, C	179.5, C	180.9, C	n.d.^*d*^, C	173.6, C	180.3, C	179.0, C
8	36.0, CH_2_	30.9, CH_2_	36.2, CH_2_	36.3, CH_2_	30.6, CH_2_	31.3, CH_2_	33.0, CH_2_	31.9, CH_2_	32.3, CH_2_	32.2, CH_2_	34.8, CH_2_
9	120.6, CH	120.7, CH	120.2, CH	120.3, CH	119.0, CH	121.3, CH	122.0, CH	122.4, CH	119.0, CH	120.5, CH	120.9, CH
10	135.9, C	135.5, C	136.0, C	135.8, C	135.3, C	134.6, C	134.0, C	133.9, C	133.9, C	132.6, C	135.4, C
11	18.2, CH_3_	18.0, CH_3_	18.0, CH_3_	18.1, CH_3_	18.0, CH_3_	18.1, CH_3_	18.0, CH_3_	18.1, CH_3_	17.7, CH_3_	17.7, CH_3_	18.1, CH_3_
12	26.3, CH_3_	26.1, CH_3_	26.1, CH_3_	26.1, CH_3_	26.1, CH_3_	26.2, CH_3_	26.1, CH_3_	26.2, CH_3_	25.8, CH_3_	26.0, CH_3_	26.1, CH_3_
13	169.1, C	168.5, C	n.d.^*d*^, C	61.6, CH_2_	62.8, CH_2_	64.0, CH_2_	67.3, CH_2_	67.6, CH_2_	60.7, CH_2_	70.7, CH_2_	181.0, C
5-OCOCH_3_			21.2, CH_3_	21.1, CH_3_							
5-OCOCH_3_			172.1, C	172.1, C							
13-OCOCH_3_					21.0, CH_3_					20.9, CH_3_	
13-OCOCH_3_					170.9, C					170.9, C	
1'						174.1, C					
2'						30.3, CH_2_					
3'						30.2, CH_2_					
4'						176.7, C					

^a^Measured in CD_3_OD;

^b^Measured in CDCl_3_;

^c^Measured in DMSO-*d*_6_;

^d^n.d., not detected;

^e^Measured at 150 MHz;

^f^Measured at 125 MHz

**Table 3:** ^1^H NMR spectroscopic data of compounds **7**–**11**

No	**7**^ac^	**8**^ac^	**9**^bd^	**10**^bc^	**11**^ad^
2	2.31, overlapped 1.33, dd (13.0, 8.9)	1.80, m 1.65, overlapped	3.38, s	3.45, s	2.44, dd (13.5, 8.6) 1.93, dd (13.5, 9.1)
3	2.21, m	2.34, m			2.86, dddd (9.8, 9.1, 8.6, 6.6)
4	2.10, ddd (13.6, 9.4, 5.3) 1.43, ddd (13.6, 5.9, 3.8)	1.90, dd (13.5, 6.7) 1.76, dd (13.5, 9.6)	2.19, dd (14.9, 6.3) 1.87, dd (14.9, 1.0)	1.73, dd (13.1, 6.9) 1.61, dd (13.1, 10.6)	2.31, ddd (14.0, 9.8, 5.5) 2.00, ddd (14.0, 6.6, 4.0)
5	4.27, dd (5.3, 3.8)	4.31, br. s	3.76, dd (6.3, 1.0)	3.57, dd (10.6, 6.9)	3.99, dd (5.5, 4.0)
8	2.51, dd (14.3, 7.2) 2.33, overlapped	2.51, dd (13.8, 7.4) 2.29, dd (13.8, 7.4)	2.31, dd (14.1, 7.1) 2.01, dd (14.1, 8.3)	2.29, dd (14.2, 7.6) 2.24, dd (14.2, 7.6)	2.41, dd (14.3, 7.5) 2.10, dd (14.3, 7.5)
9	5.13, t (7.2)	5.14, t (7.4)	5.08, t-like (7.7)	5.22, t (7.6)	5.10, t (7.5)
11	1.63, s	1.64, s	1.55, s	1.58, s	1.64, s
12	1.68, s	1.68, s	1.66, s	1.67, s	1.71, s
13	3.49, dd (10.4, 6.0) 3.46, dd (10.4, 6.0)	3.46, dd (10.3, 6.9) 3.42, dd (10.3, 6.5)	3.64, d (12.4) 3.52, d (12.4)	3.97, d (11.3) 3.89, d (11.3)	
5-OCOCH_3_					
13-OCOCH_3_				1.99, s	
3-OH				4.60, s	
13-OH			4.90, br. s		

^a^Measured in CD_3_OD;

^b^Measured in DMSO-*d*_6_;

^c^Measured at 600 MHz;

^d^Measured at 500 MHz

The molecular formula of compound **8** (Fig. 1) was deduced to be C_12_H_20_O_4_, which was identical with **7**. Detailed interpretation of the NMR of **8** revealed the same planar structure as **7** (Tables 2 and 3). The lack of key ROESY signals between H-5 and H-8 indicated a 1R*,3S*,5R* configuration of **8** (Fig. 3). Considering the same biosynthetic pathways of **7** and **8**, the absolute configurations of **8** were determined to be 1*R*,3*S*,5*R*. Compound **8** was trivially named hirsutavibrin H.

Hirsutavibrin I (**9**, Fig. 1) was isolated as a colorless oil. The (+)-HRESIMS sodium adduct ion peak at *m/z* 265.10452 [M + Na]^+^ (calcd for C_12_H_18_O_5_Na, 265.10519) revealed a chemical formula of C_12_H_18_O_5_ of **9**. The ^13^C NMR data of **9** (Tables 2 and 3) displayed two methyls, three methylenes (one oxygenated), two oxygenated sp^3^ methines (*δ*_C_ 63.9, 75.0), two sp^3^ quaternary carbons (*δ*_C_ 59.7, 66.8), etc. These data are different from those of compounds **1**–**6**, while are similar to those of compound **7**, and are highly resemble to those of vibralactone B [20, 37]. Compound **9** was 18 Da larger than vibralactone B, suggesting that **9** was the lactone ring-opening product of vibralactone B. The crucial ROESY correlations of H-2 (*δ*_H_ 3.38)/H-8 (*δ*_H_ 2.31, 2.01)/H-5 (*δ*_H_ 3.76), H-2/H-9 (*δ*_H_ 5.08) indicated the 1*S*,2*S*,3*S*,5*S* configuration of **9**. Hence compound **9** was elucidated as depicted in Fig. 1.

The chemical formula of **10**, obtained as a colorless oil, was determined to be C_14_H_22_O_7_ (*m/z* 303.14383 [M + H]^+^, Δ 0.00048 ppm) by (+)-HRESIMS. The 1D NMR data between **10** (Tables 2 and 3) and **5** were quite similar, except that the C-2/C-3 double bond between in **5** was replaced by a vicinal diol in **10**, according to the key HMBC correlations from H-2 (*δ*_H_ 3.45) to C-1 (*δ*_C_ 54.3), C-3 (*δ*_C_ 76.9), C-5 (*δ*_C_ 70.6), from H-8 (*δ*_H_ 2.29) to C-2 (*δ*_C_ 83.7), and from H-13 (*δ*_H_ 3.97) to C-2, C-3, C-4 (*δ*_C_ 45.0), and from 3-OH (*δ*_H_ 4.60) to C-2, C-3, C-4, and C-13 (Fig. 2). The crucial ROESY correlations of H-2 (*δ*_H_ 3.45)/H-8/H-5 (*δ*_H_ 3.57)/3-OH (Fig. 3) indicated the 1*S*,2*S*,3*R*,5*S* stereochemistry of **10**. Therefore, the structure of compound **10** was established and named hirsutavibrin J.

( +)-HRESIMS spectrometric analysis established the molecular formula of **11** (Fig. 1) as C_12_H_18_O_5_Na (*m/z* 265.10461 [M + Na]^+^, mass error 0.11536 ppm). The 1D NMR spectroscopic data of **11** was similar to those of compound **7**, except with the existence of a new carbonyl carbon (*δ*_C_ 181.0) in **11** while absence of the hydroxymethyl group in **7**. This evidence indicated that compound **11** differs from **7** by the oxygenated status of C-13. The carbonyl group at *δ*_C_ 181.0 in compound **11** was assigned to a carboxylic group (C-13). The correlation from H-3 (*δ*_H_ 2.86) to C-13 in the HMBC spectrum (Fig. 2) as well as the molecular formula corroborated the above assignments. The vital ROESY correlations of H-8/H-5/H-3 suggested a 1*R*,3*S*,5*S* configuration of compound **11** (Fig. 3). Therefore, the structure of compound **11** was established and named hirsutavibrin K.

### Biological activity evaluation of 1–11

The isolated compounds with abundant yield (**1**, **2**, **4**, **6**) were subjected to a panel of biological activity screening, including the cytotoxicity against A549, a human lung cancer cell line, and anti-NO (nitric oxide) activity in murine monocytic RAW 264.7 macrophages. As a result, compounds **1** and **2** showed weak cytotoxicity activity toward A549 with the IC_50_ 39.7 and 34.3 μM, respectively (positive control cisplatin, IC_50_ 5.09 μM). Compound **4** displayed anti-NO activity with IC_50_ of 26.6, which were more significant than the positive control L-*N*^G^-monomethyl arginine (IC_50_ 51.2 μM).

## Conclusion

In conclusion, eleven new vibralactone derivatives (**1 − 11**) were obtained from the co-culture broth of the two basidiomycetous fungi *Stereum hirsutum* and *Boreostereum vibrans*. Comprehensive analyses of 1D & 2D NMR spectroscopic data and computational calculations allowed the establishment of the structures of all the isolates. Most of the isolates are structurally similar to vibralactone but display distinct configurations.

Co-culturing microbes typically involves ascomycetes or ascomycetes with bacteria, with few studies focusing on secondary metabolite discovery from co-cultures of two basidiomycetes. Our research demonstrates that co-culturing phylogenetically related fungal species is an effective approach for discovering natural products. For instance, previous studies on *Boreostereum vibrans* cultures primarily yielded vibralactone derivatives with the same absolute configuration as vibralactone. However, when *Boreostereum vibrans* was co-cultured with *Stereum hirsutum*, vibralactone derivatives with distinct configurations and additional modifications were discovered. Notably, none of the isolated compounds have been found from the individual cultures of each fungus. While further evidence is needed to confirm the involvement of new biosynthetic pathways in the modification of vibralactone, this study underscores that co-culturing two basidiomycetes is a promising strategy for natural product discovery. This work not only expands the members of vibralactone derivatives with different configurations but also opens a new avenue for fungal co-culturing study between congeneric fungi.

## Supplementary Information

Additional file 1. The NMR, HRESIMS spectra, and calculation details of the isolates.

## Data Availability

The datasets generated during and/or analyzed during the current study are available from the corresponding author on reasonable request.
