# Supplementary material for: Vibralactone derivatives isolated from co-cultures of the basidiomycetes Stereum hirsutum and Boreostereum vibrans
Source: Nat Prod Bioprospect. 2025 Mar 31;15(1):20. doi: 10.1007/s13659-025-00505-y (PMC11958929; doi:10.1007/s13659-025-00505-y)
Supplement: Supplementary file 1 — Additional file 1. The NMR, HRESIMS spectra, and calculation details of the isolates. [file 13659_2025_505_MOESM1_ESM.docx]

**Supplementary material for**

**Vibralactone Derivatives Isolated from Co-cultures of** **the Basidiomycetes *Stereum hirsutum* and *Boreostereum vibrans***

Jinjuan Wei^1^, Zhe-Xi Li^1^, Gao-Ke Peng^1^, Xinyang Li, He-Ping Chen^*^, Ji-Kai Liu^*^

School of Pharmaceutical Sciences, South-Central Minzu University, Wuhan 430074, China

^1^These authors contribute equally to this work.

Corresponding authors:

[chenhp@mail.scuec.edu.cn](mailto:chenhp@mail.scuec.edu.cn) (H.-P. Chen)

[liujikai@mail.scuec.edu.cn](mailto:liujikai@mail.scuec.edu.cn) (J.-K. Liu)

Contents

[1. Supplementary Figures of Compounds **1**–**11**. 2](#_Toc191581707)

[Figure S1. ^1^H NMR spectrum of **1** (600 MHz, CD_3_OD). 2](#_Toc191581708)

[Figure S2. ^13^C and DEPT NMR spectra of **1** (150 MHz, CD_3_OD). 2](#_Toc191581709)

[Figure S3. HSQC spectrum of **1**. 3](#_Toc191581710)

[Figure S4. ^1^H-^1^H COSY spectrum of **1**. 3](#_Toc191581711)

[Figure S5. HMBC spectrum of **1**. 4](#_Toc191581712)

[Figure S6. ROESY spectrum of **1**. 4](#_Toc191581713)

[Figure S7. HRESIMS report of **1**. 5](#_Toc191581714)

[Figure S8. ^1^H NMR spectrum of **2** (500 MHz, CD_3_OD). 6](#_Toc191581715)

[Figure S9. ^13^C and DEPT NMR spectra of **2** (125 MHz, CD_3_OD). 6](#_Toc191581716)

[Figure S10. HSQC spectrum of **2**. 7](#_Toc191581717)

[Figure S11. ^1^H-^1^H COSY spectrum of **2**. 7](#_Toc191581718)

[Figure S12. HMBC spectrum of **2**. 8](#_Toc191581719)

[Figure S13. ROESY spectrum of **2**. 8](#_Toc191581720)

[Figure S14. HRESIMS report of **2**. 9](#_Toc191581721)

[Figure S15. ^1^H NMR spectrum of **3** (600 MHz, CD_3_OD). 10](#_Toc191581722)

[Figure S16. ^13^C and DEPT NMR spectra of **3** (150 MHz, CD_3_OD). 10](#_Toc191581723)

[Figure S17. HSQC spectrum of **3**. 11](#_Toc191581724)

[Figure S18. ^1^H-^1^H COSY spectrum of **3**. 11](#_Toc191581725)

[Figure S19. HMBC spectrum of **3**. 12](#_Toc191581726)

[Figure S20. ROESY spectrum of **3**. 12](#_Toc191581727)

[Figure S21. HRESIMS report of **3**. 13](#_Toc191581728)

[Figure S22. ^1^H NMR spectrum of **4** (600 MHz, CD_3_OD). 14](#_Toc191581729)

[Figure S23. ^13^C and DEPT NMR spectra of **4** (150 MHz, CDCl_3_). 14](#_Toc191581730)

[Figure S24. HSQC spectrum of **4**. 15](#_Toc191581731)

[Figure S25. ^1^H-^1^H COSY spectrum of **4**. 15](#_Toc191581732)

[Figure S26. HMBC spectrum of **4**. 16](#_Toc191581733)

[Figure S27. ROESY spectrum of **4**. 16](#_Toc191581734)

[Figure S28. HRESIMS report of **4**. 17](#_Toc191581735)

[Figure S29. ^1^H NMR spectrum of **5** (600 MHz, CDCl_3_). 18](#_Toc191581736)

[Figure S30. ^13^C and DEPT NMR spectra of **5** (150 MHz, CDCl_3_). 18](#_Toc191581737)

[Figure S31. HSQC spectrum of **5**. 19](#_Toc191581738)

[Figure S32. ^1^H-^1^H COSY spectrum of **5**. 19](#_Toc191581739)

[Figure S33. HMBC spectrum of **5**. 20](#_Toc191581740)

[Figure S34. ROESY spectrum of **5**. 20](#_Toc191581741)

[Figure S35. HRESIMS report of **5**. 21](#_Toc191581742)

[Figure S36. ^1^H NMR spectrum of **6** (600 MHz, CD_3_OD). 22](#_Toc191581743)

[Figure S37. ^13^C and DEPT NMR spectra of **6** (150 MHz, CD_3_OD). 22](#_Toc191581744)

[Figure S38. HSQC spectrum of **6**. 23](#_Toc191581745)

[Figure S39. ^1^H-^1^H COSY spectrum of **6**. 23](#_Toc191581746)

[Figure S40. HMBC spectrum of **6**. 24](#_Toc191581747)

[Figure S41. ROESY spectrum of **6**. 24](#_Toc191581748)

[Figure S42. HRESIMS report of **6**. 25](#_Toc191581749)

[Figure S43. ^1^H NMR spectrum of **7** (600 MHz, CD_3_OD). 26](#_Toc191581750)

[Figure S44. ^13^C and DEPT NMR spectra of **7** (150 MHz, CD_3_OD). 26](#_Toc191581751)

[Figure S45. HSQC spectrum of **7**. 27](#_Toc191581752)

[Figure S46. ^1^H-^1^H COSY spectrum of **7**. 27](#_Toc191581753)

[Figure S47. HMBC spectrum of **7**. 28](#_Toc191581754)

[Figure S48. ROESY spectrum of **7**. 28](#_Toc191581755)

[Figure S49. HRESIMS report of **7**. 29](#_Toc191581756)

[Figure S50. ^1^H NMR spectrum of **8** (600 MHz, CD_3_OD). 30](#_Toc191581757)

[Figure S51. ^13^C and DEPT NMR spectra of **8** (150 MHz, CD_3_OD). 30](#_Toc191581758)

[Figure S52. HSQC spectrum of **8**. 31](#_Toc191581759)

[Figure S53. ^1^H-^1^H COSY spectrum of **8**. 31](#_Toc191581760)

[Figure S54. HMBC spectrum of **8**. 32](#_Toc191581761)

[Figure S55. ROESY spectrum of **8**. 32](#_Toc191581762)

[Figure S56. HRESIMS report of **8**. 33](#_Toc191581763)

[Figure S64. ^1^H NMR spectrum of **9** (500 MHz, DMSO-*d*_6_). 34](#_Toc191581764)

[Figure S65. ^13^C and DEPT NMR spectra of **9** (125 MHz, DMSO-*d*_6_). 34](#_Toc191581765)

[Figure S66. HSQC spectrum of **9**. 35](#_Toc191581766)

[Figure S67. ^1^H-^1^H COSY spectrum of **9**. 35](#_Toc191581767)

[Figure S68. HMBC spectrum of **9**. 36](#_Toc191581768)

[Figure S69. ROESY spectrum of **9**. 36](#_Toc191581769)

[Figure S70. HRESIMS report of **9**. 37](#_Toc191581770)

[Figure S71. ^1^H NMR spectrum of **10** (600 MHz, DMSO-*d*_6_). 38](#_Toc191581771)

[Figure S72. ^13^C and DEPT NMR spectra of **10** (150 MHz, DMSO-*d*_6_). 38](#_Toc191581772)

[Figure S73. HSQC spectrum of **10**. 39](#_Toc191581773)

[Figure S74. ^1^H-^1^H COSY spectrum of **10**. 39](#_Toc191581774)

[Figure S75. HMBC spectrum of **10**. 40](#_Toc191581775)

[Figure S76. ROESY spectrum of **10**. 40](#_Toc191581776)

[Figure S77. HRESIMS report of **10**. 41](#_Toc191581777)

[Figure S78. ^1^H NMR spectrum of **11** (500 MHz, CD_3_OD). 42](#_Toc191581778)

[Figure S79. ^13^C and DEPT NMR spectra of **11** (125 MHz, CD_3_OD). 42](#_Toc191581779)

[Figure S80. HSQC spectrum of **11**. 43](#_Toc191581780)

[Figure S81. ^1^H-^1^H COSY spectrum of **11**. 43](#_Toc191581781)

[Figure S82. HMBC spectrum of **11**. 44](#_Toc191581782)

[Figure S83. ROESY spectrum of **11**. 44](#_Toc191581783)

[Figure S84. HRESIMS report of **11**. 45](#_Toc191581784)

[Figure S85. CD spectrum of **1**. 46](#_Toc191581785)

[Figure S86. CD spectrum of **2**. 47](#_Toc191581786)

[Figure S87. CD spectrum of **3**. 48](#_Toc191581787)

[Figure S89. CD spectrum of **5**. 49](#_Toc191581788)

[Figure S90. CD spectrum of **6**. 50](#_Toc191581789)

[Figure S91. CD spectrum of **7**. 51](#_Toc191581790)

[2. TLC analysis of the coculture condition. 52](#_Toc191581791)

[3. Calculation details. 52](#_Toc191581792)

[3.1 ECD calculation of **1**. 52](#_Toc191581793)

[3.2 ECD calculation of **2**. 55](#_Toc191581794)

[3.3 ECD calculation of **3** 58](#_Toc191581795)

[3.5 ECD calculation of **5** 63](#_Toc191581796)

[3.6 ECD calculation of **6** 67](#_Toc191581797)

[3.7 ECD calculation of **7** 70](#_Toc191581798)

[4. Experimental section 72](#_Toc191581799)

[4.1 General experimental procedures 72](#_Toc191581800)

[4.2 Fungal material 72](#_Toc191581801)

[4.3 Extraction and isolation 72](#_Toc191581802)

[4.4 Characterization data 73](#_Toc191581803)

[4.4.1 *Hirsutavibrin A (****1****)* 73](#_Toc191581804)

[4.4.2  *Hirsutavibrin B (****2****)* 73](#_Toc191581805)

[4.4.3  *Hirsutavibrin C (****3****)* 73](#_Toc191581806)

[4.4.4 *Hirsutavibrin D (****4****)* 74](#_Toc191581807)

[4.4.5 *Hirsutavibrin E (****5****)* 74](#_Toc191581808)

[4.4.6 *Hirsutavibrin F (****6****)* 74](#_Toc191581809)

[4.4.7 *Hirsutavibrin G (****7****)* 74](#_Toc191581810)

[4.4.8 *Hirsutavibrin H (****8****)* 74](#_Toc191581811)

[4.4.9 *Hirsutavibrin I (****9****)* 74](#_Toc191581812)

[4.4.10 *Hirsutavibrin J (****10****)* 74](#_Toc191581813)

[4.4.11 *Hirsutavibrin K (****11****)* 74](#_Toc191581814)

[4.5 Biological Activity Assessment 74](#_Toc191581815)

# Supplementary Figures of Compounds 1–11.

## Figure S1. ^1^H NMR spectrum of 1 (600 MHz, CD_3_OD).

## Figure S2. ^13^C and DEPT NMR spectra of 1 (150 MHz, CD_3_OD).

## Figure S3. HSQC spectrum of 1.

## Figure S4. ^1^H-^1^H COSY spectrum of 1.

## Figure S5. HMBC spectrum of 1.

## Figure S6. ROESY spectrum of 1.

## Figure S7. HRESIMS report of 1.


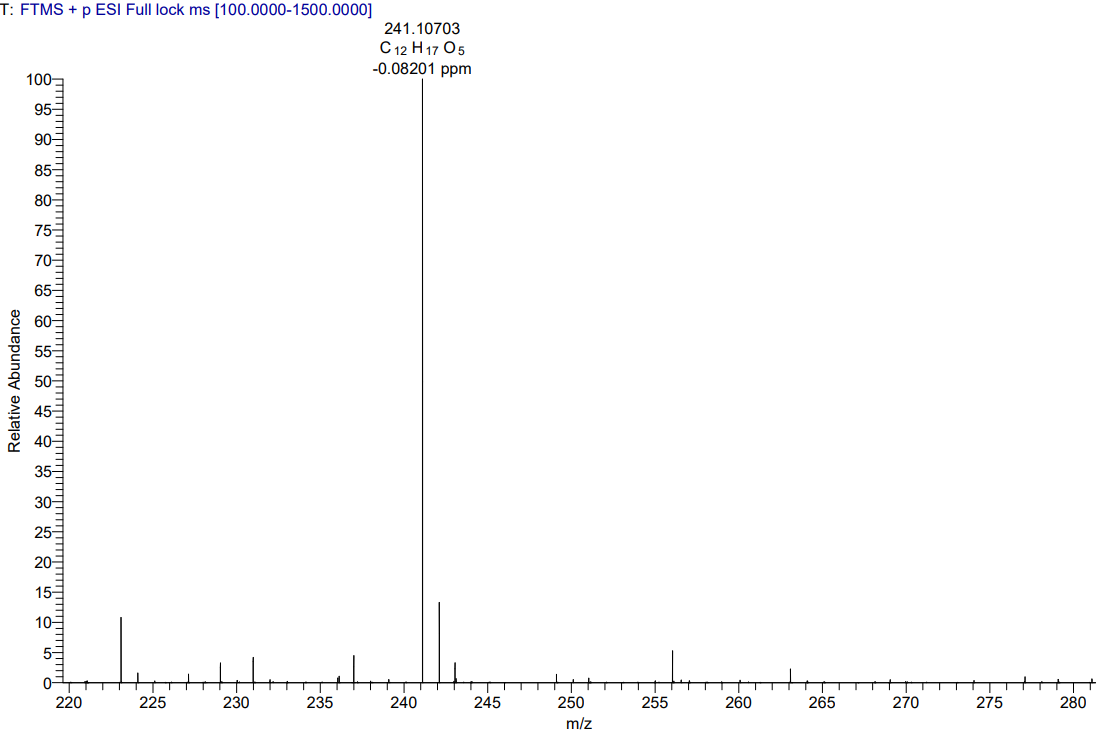


## Figure S8. ^1^H NMR spectrum of 2 (500 MHz, CD_3_OD).

## Figure S9. ^13^C and DEPT NMR spectra of 2 (125 MHz, CD_3_OD).

## Figure S10. HSQC spectrum of 2.

## Figure S11. ^1^H-^1^H COSY spectrum of 2.

## Figure S12. HMBC spectrum of 2.

## Figure S13. ROESY spectrum of 2.

## Figure S14. HRESIMS report of 2.


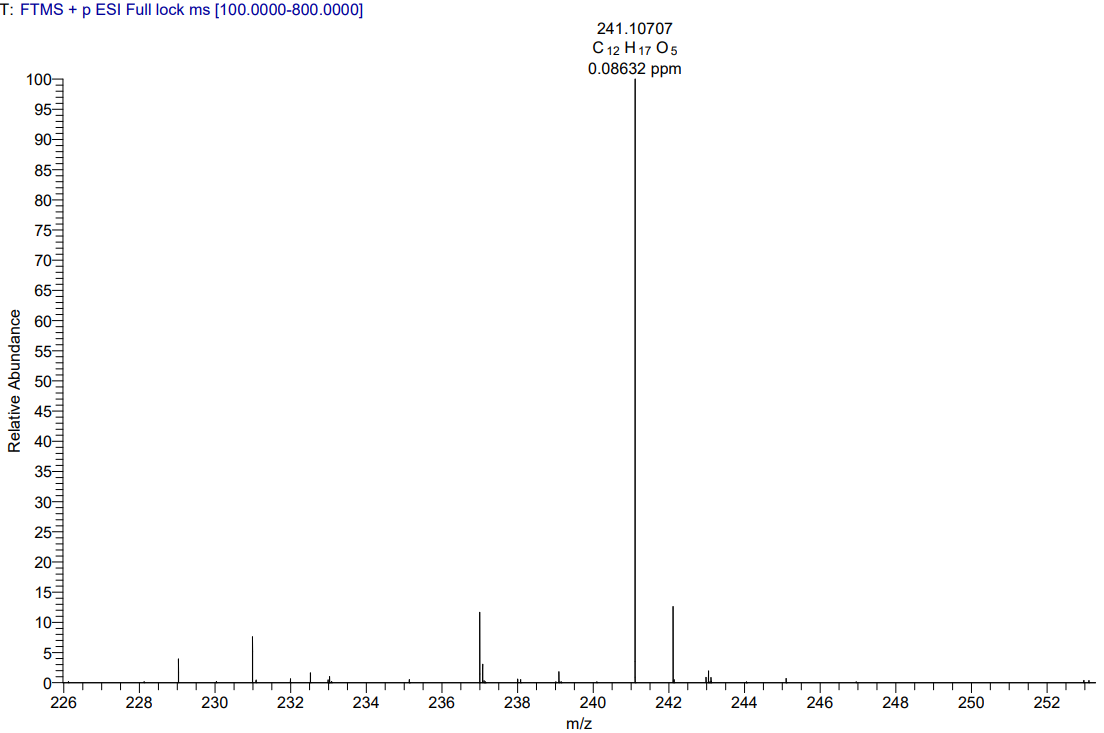


## Figure S15. ^1^H NMR spectrum of 3 (600 MHz, CD_3_OD).

## Figure S16. ^13^C and DEPT NMR spectra of 3 (150 MHz, CD_3_OD).

## Figure S17. HSQC spectrum of 3.

**Figure S18.** ^1^H-^1^H COSY spectrum of **3**.

## Figure S19. HMBC spectrum of 3.

**Figure S20.** ROESY spectrum of **3**.

## Figure S21. HRESIMS report of 3.


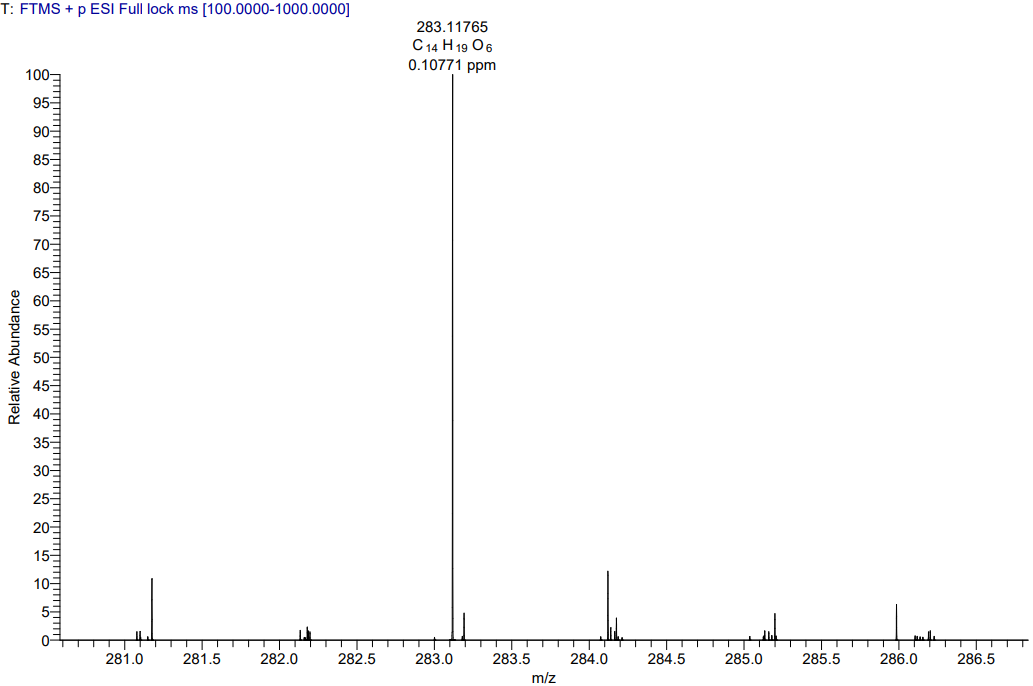


## Figure S22. ^1^H NMR spectrum of 4 (600 MHz, CD_3_OD).

## Figure S23. ^13^C and DEPT NMR spectra of 4 (150 MHz, CDCl_3_).

## Figure S24. HSQC spectrum of 4.

**Figure S25.** ^1^H-^1^H COSY spectrum of **4**.

## Figure S26. HMBC spectrum of 4.

**Figure S27.** ROESY spectrum of **4**.

## Figure S28. HRESIMS report of 4.


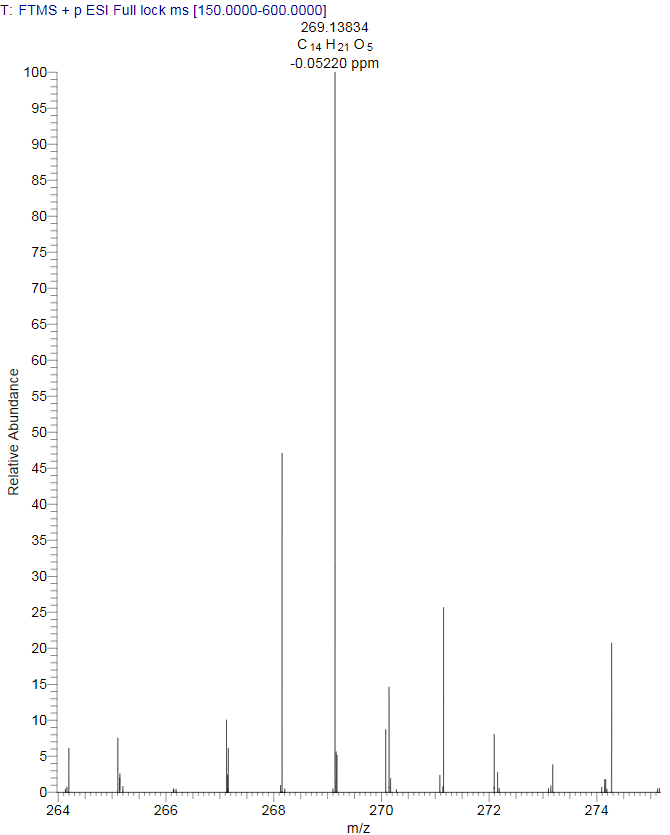


## Figure S29. ^1^H NMR spectrum of 5 (600 MHz, CDCl_3_).

## Figure S30. ^13^C and DEPT NMR spectra of 5 (150 MHz, CDCl_3_).

## Figure S31. HSQC spectrum of 5.

## Figure S32. ^1^H-^1^H COSY spectrum of 5.

## Figure S33. HMBC spectrum of 5.

**Figure S34.** ROESY spectrum of **5**.

## Figure S35. HRESIMS report of 5.


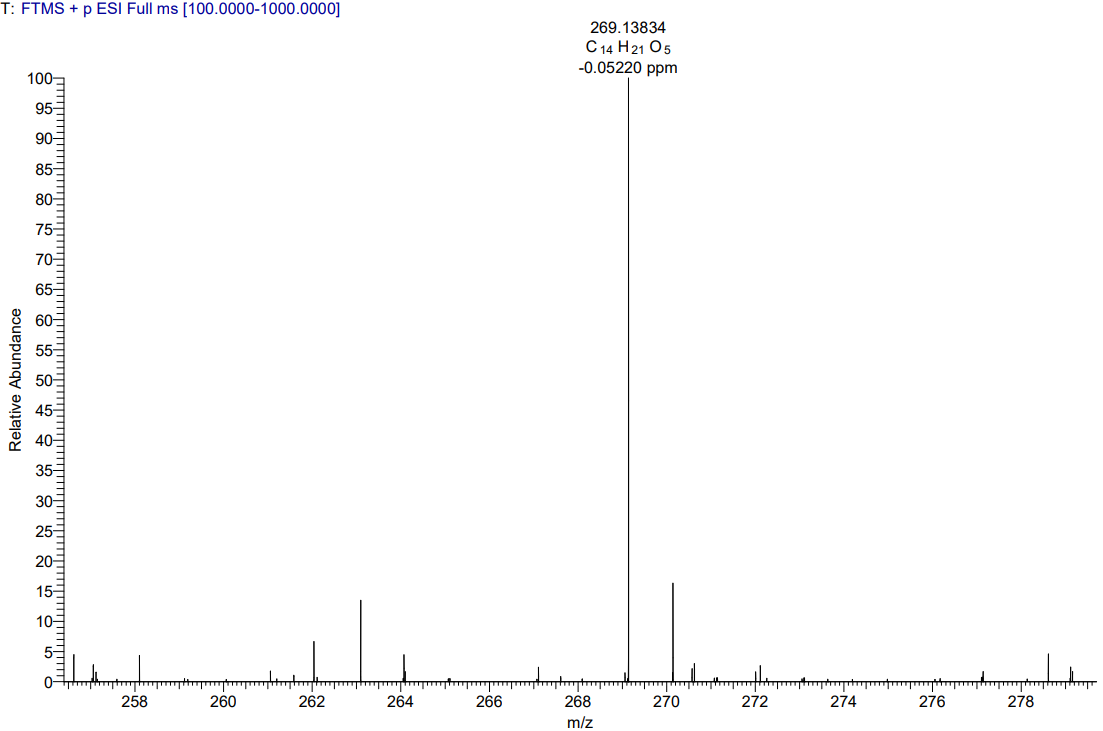


## Figure S36. ^1^H NMR spectrum of 6 (600 MHz, CD_3_OD).

## Figure S37. ^13^C and DEPT NMR spectra of 6 (150 MHz, CD_3_OD).

## Figure S38. HSQC spectrum of 6.

**Figure S39.** ^1^H-^1^H COSY spectrum of **6**.

## Figure S40. HMBC spectrum of 6.

**Figure S41.** ROESY spectrum of **6**.

## Figure S42. HRESIMS report of 6.


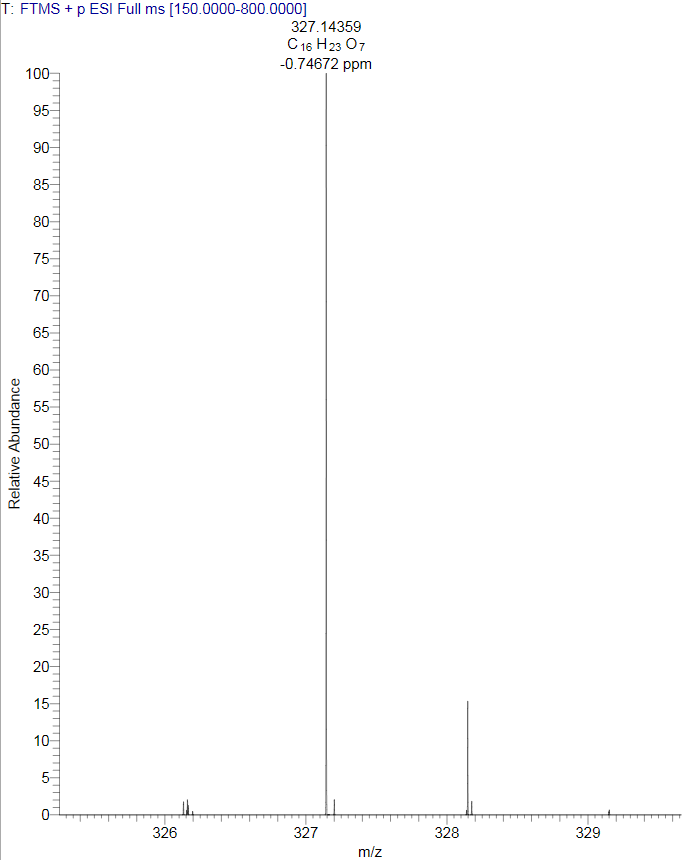


## Figure S43. ^1^H NMR spectrum of 7 (600 MHz, CD_3_OD).

## Figure S44. ^13^C and DEPT NMR spectra of 7 (150 MHz, CD_3_OD).

## Figure S45. HSQC spectrum of 7.

**Figure S46.** ^1^H-^1^H COSY spectrum of **7**.

## Figure S47. HMBC spectrum of 7.

**Figure S48.** ROESY spectrum of **7**.

## Figure S49. HRESIMS report of 7.


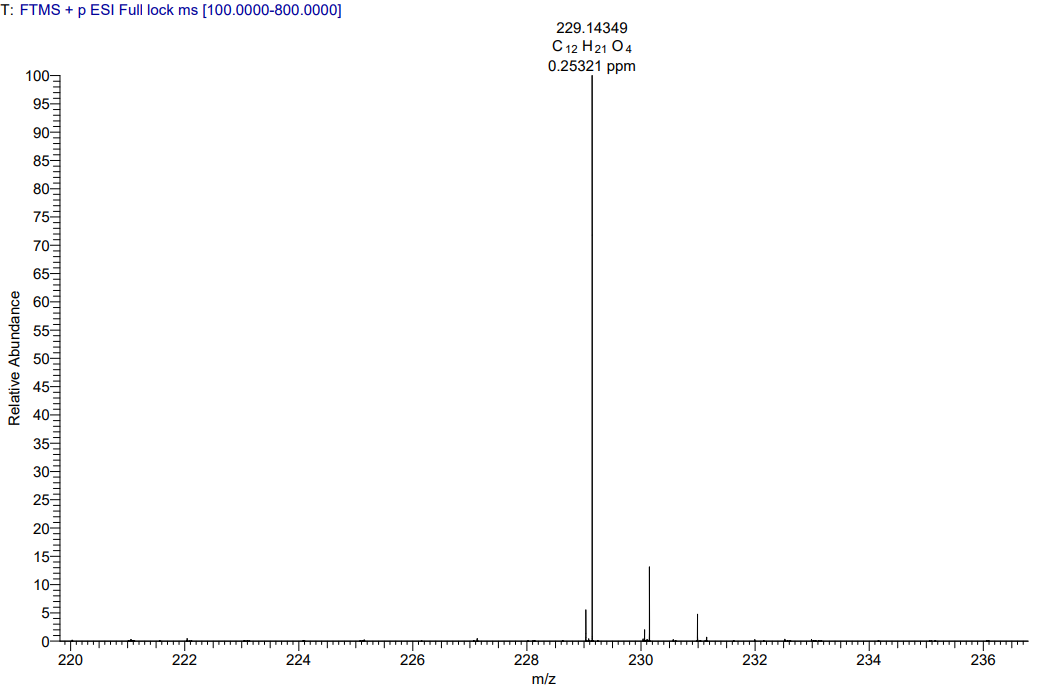


## Figure S50. ^1^H NMR spectrum of 8 (600 MHz, CD_3_OD).

## Figure S51. ^13^C and DEPT NMR spectra of 8 (150 MHz, CD_3_OD).

## Figure S52. HSQC spectrum of 8.

**Figure S53.** ^1^H-^1^H COSY spectrum of **8**.

## Figure S54. HMBC spectrum of 8.

**Figure S55.** ROESY spectrum of **8**.

## Figure S56. HRESIMS report of 8.


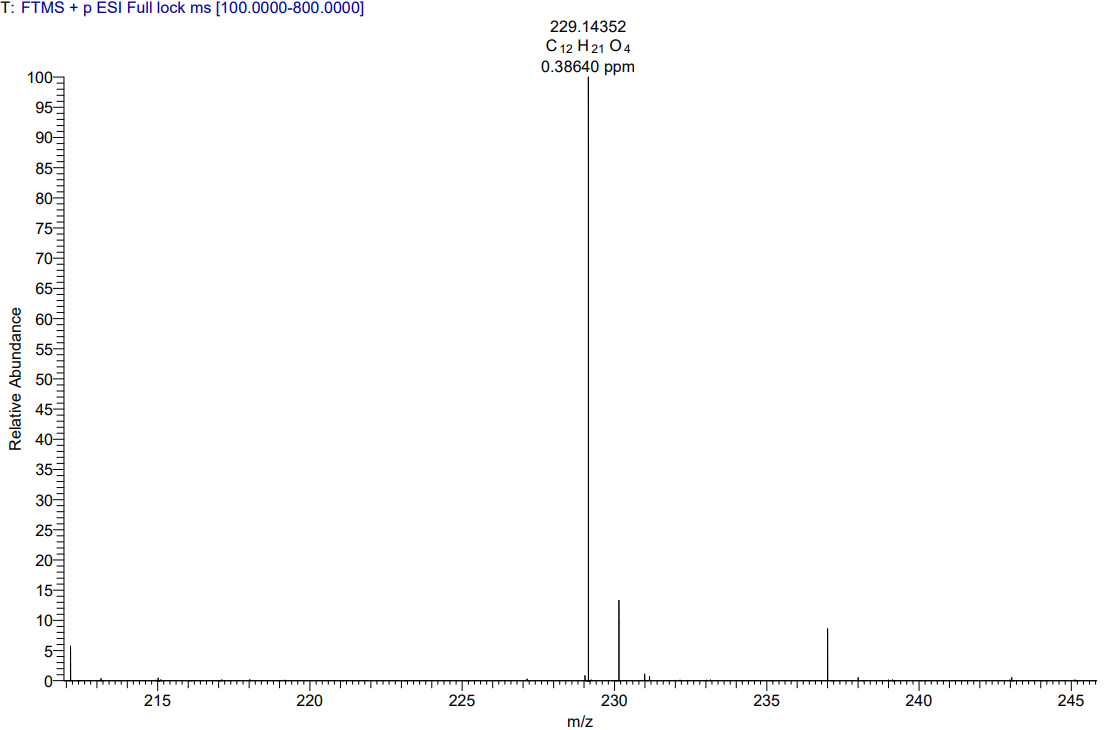


**Figure S64.** ^1^H NMR spectrum of **9** (500 MHz, DMSO-*d*_6_).

**Figure S65.** ^13^C and DEPT NMR spectra of **9** (125 MHz, DMSO-*d*_6_).

**Figure S66.** HSQC spectrum of **9**.

**Figure S67.** ^1^H-^1^H COSY spectrum of **9**.

**Figure S68.** HMBC spectrum of **9**.

**Figure S69.** ROESY spectrum of **9**.

**Figure S70.** HRESIMS report of **9**.


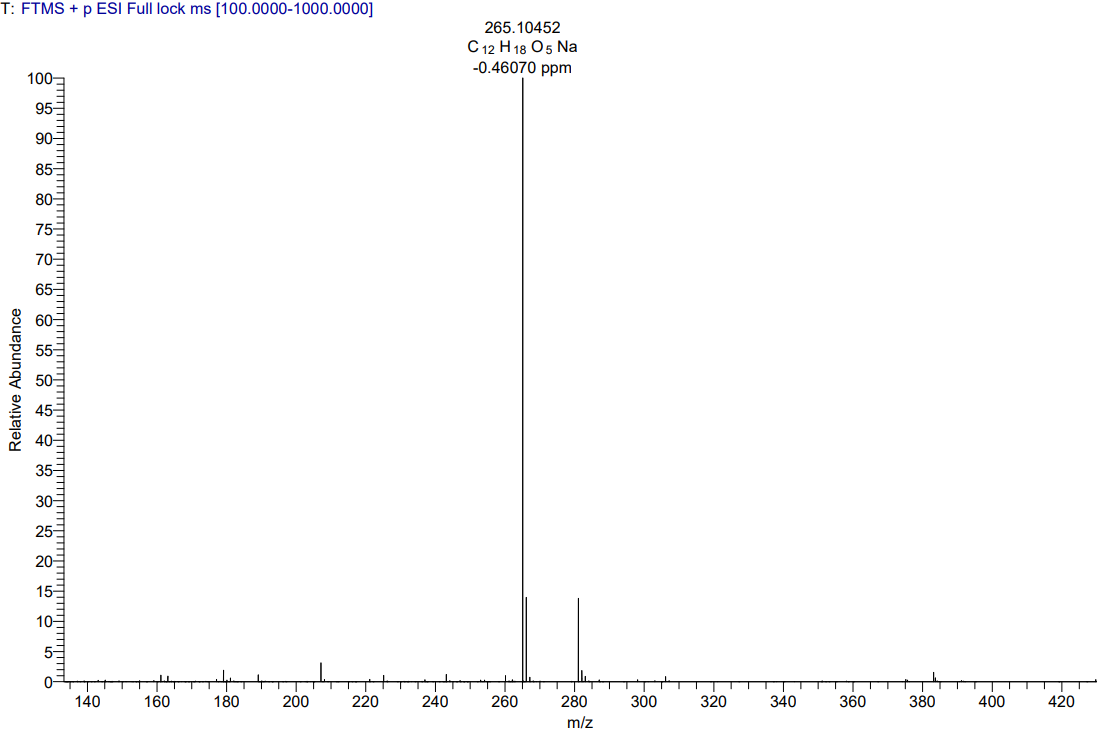


**Figure S71.** ^1^H NMR spectrum of **10** (600 MHz, DMSO-*d*_6_).

**Figure S72.** ^13^C and DEPT NMR spectra of **10** (150 MHz, DMSO-*d*_6_).

**Figure S73.** HSQC spectrum of **10**.

**Figure S74.** ^1^H-^1^H COSY spectrum of **10**.

**Figure S75.** HMBC spectrum of **10**.

**Figure S76.** ROESY spectrum of **10**.

**Figure S77.** HRESIMS report of **10**.


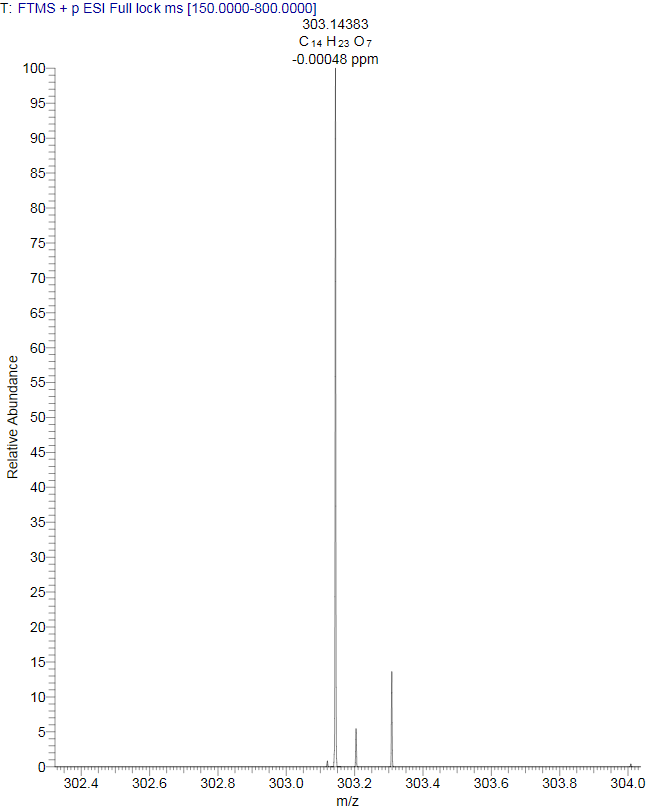


**Figure S78.** ^1^H NMR spectrum of **11** (500 MHz, CD_3_OD).

**Figure S79.** ^13^C and DEPT NMR spectra of **11** (125 MHz, CD_3_OD).

**Figure S80.** HSQC spectrum of **11**.

**Figure S81.** ^1^H-^1^H COSY spectrum of **11**.

**Figure S82.** HMBC spectrum of **11**.

**Figure S83.** ROESY spectrum of **11**.

**Figure S84.** HRESIMS report of **11**.


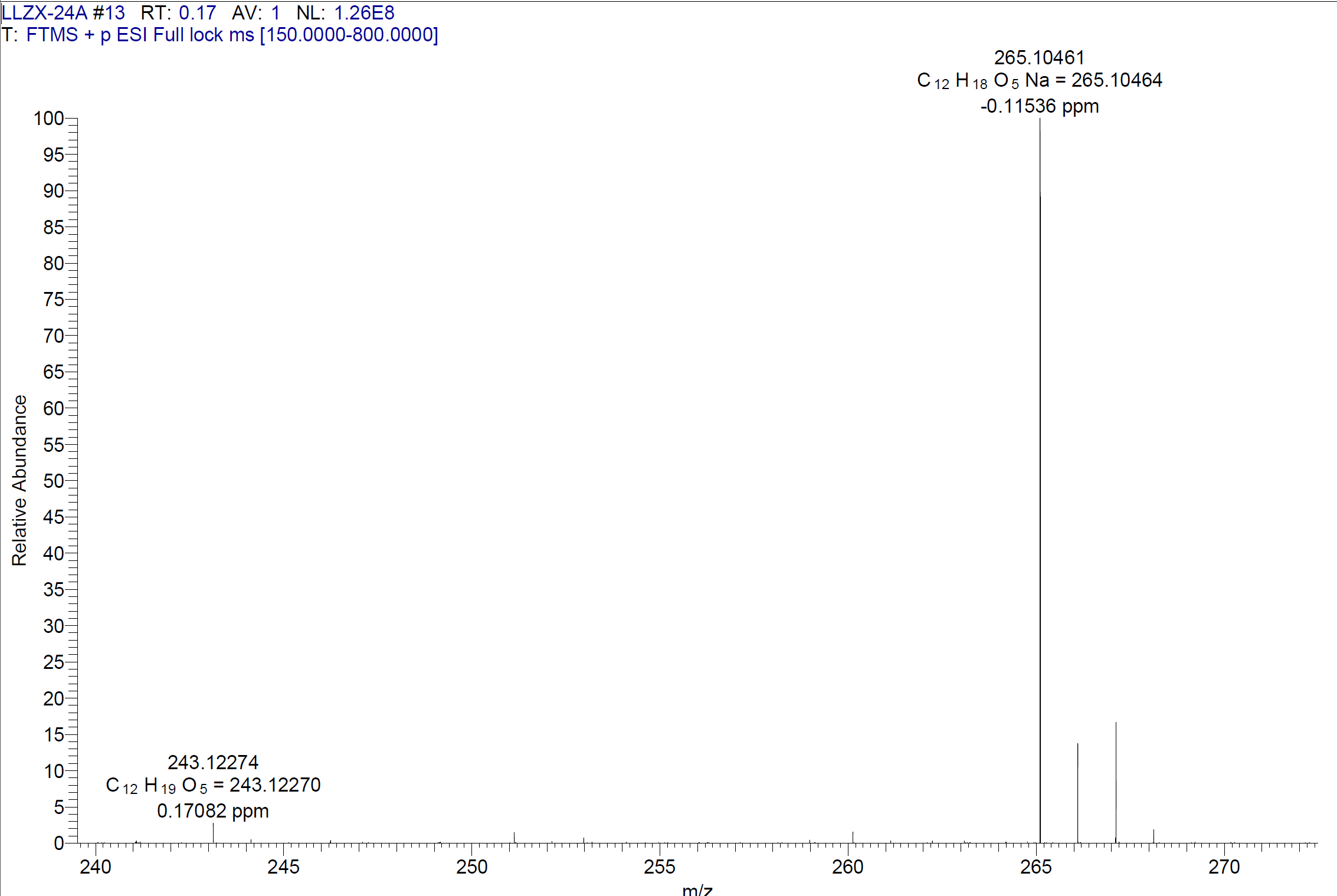


## Figure S85. CD spectrum of 1.


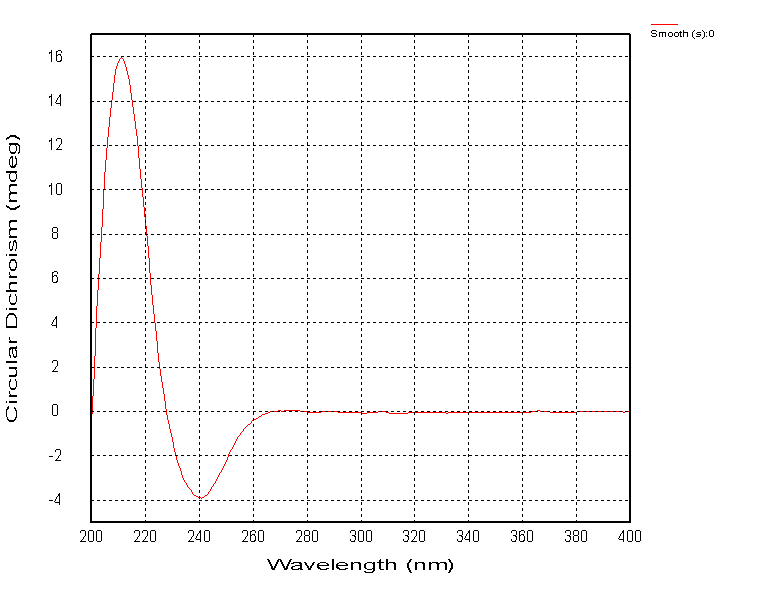


## Figure S86. CD spectrum of 2.


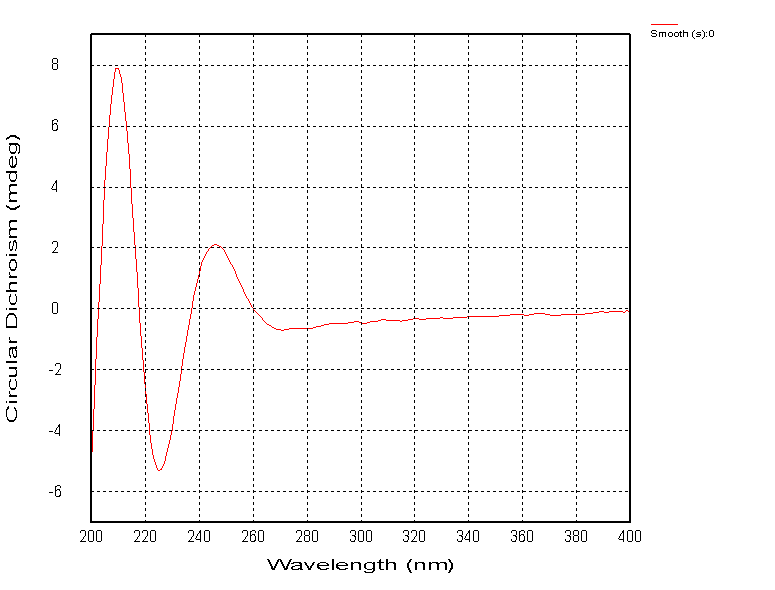


## Figure S87. CD spectrum of 3.


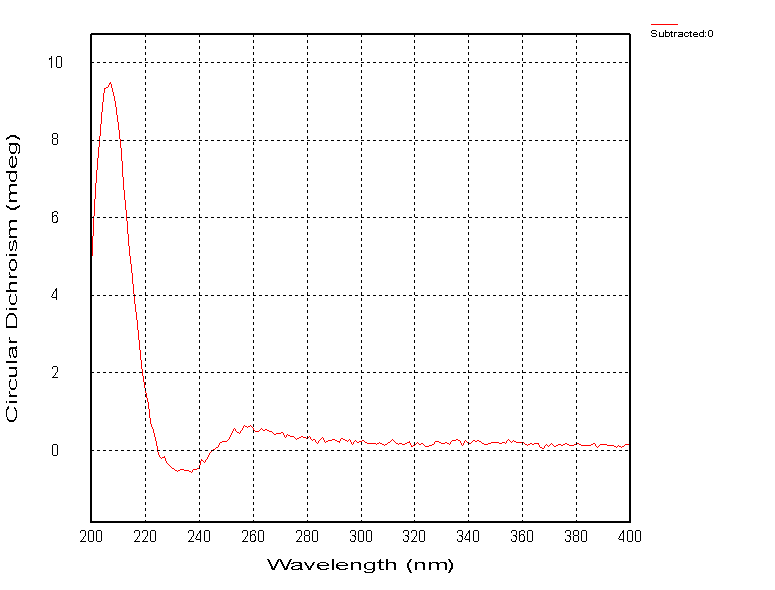


## Figure S89. CD spectrum of 5.


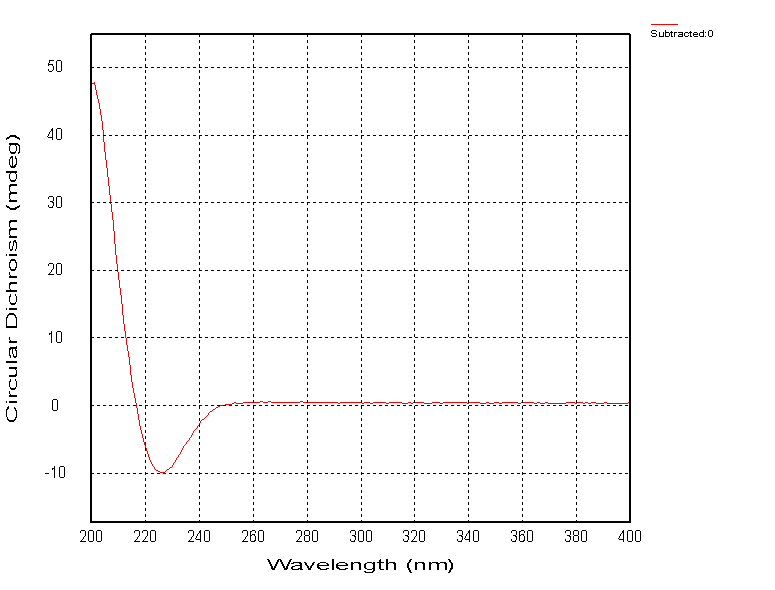


## Figure S90. CD spectrum of 6.


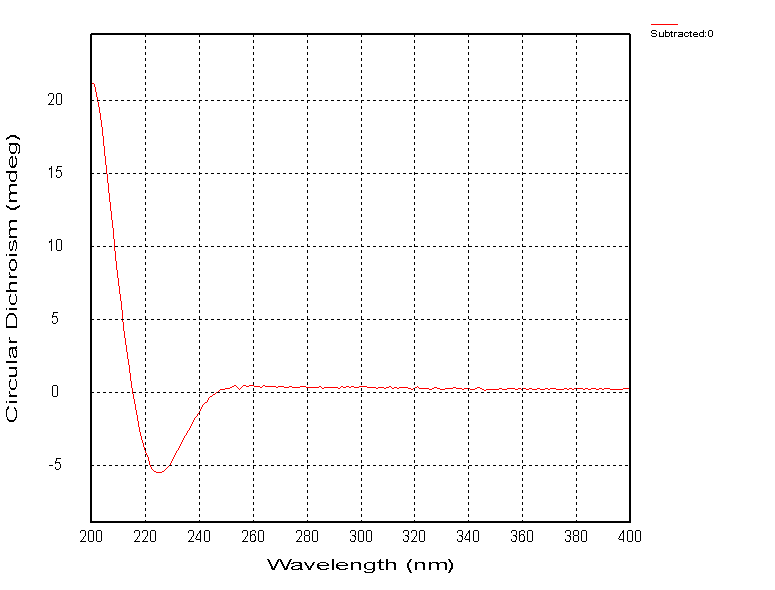


## Figure S91. CD spectrum of 7.


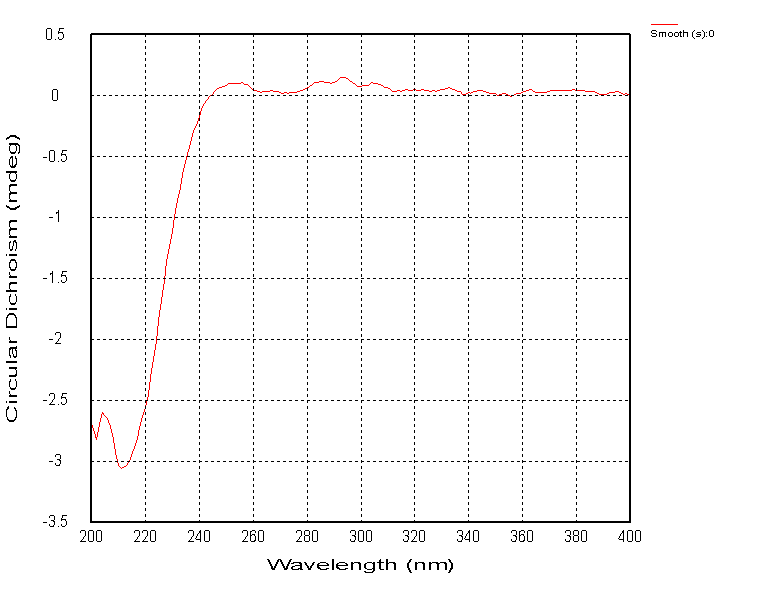


# TLC analysis of the coculture condition.

The crude extracts were fractionated by medium pressure liquid chromatography. The fractions were analysed by TLC and visualized by 10% H_2_SO_4_-ethanol-vallinin. Figure is the TLC analysis results of the culture condition.


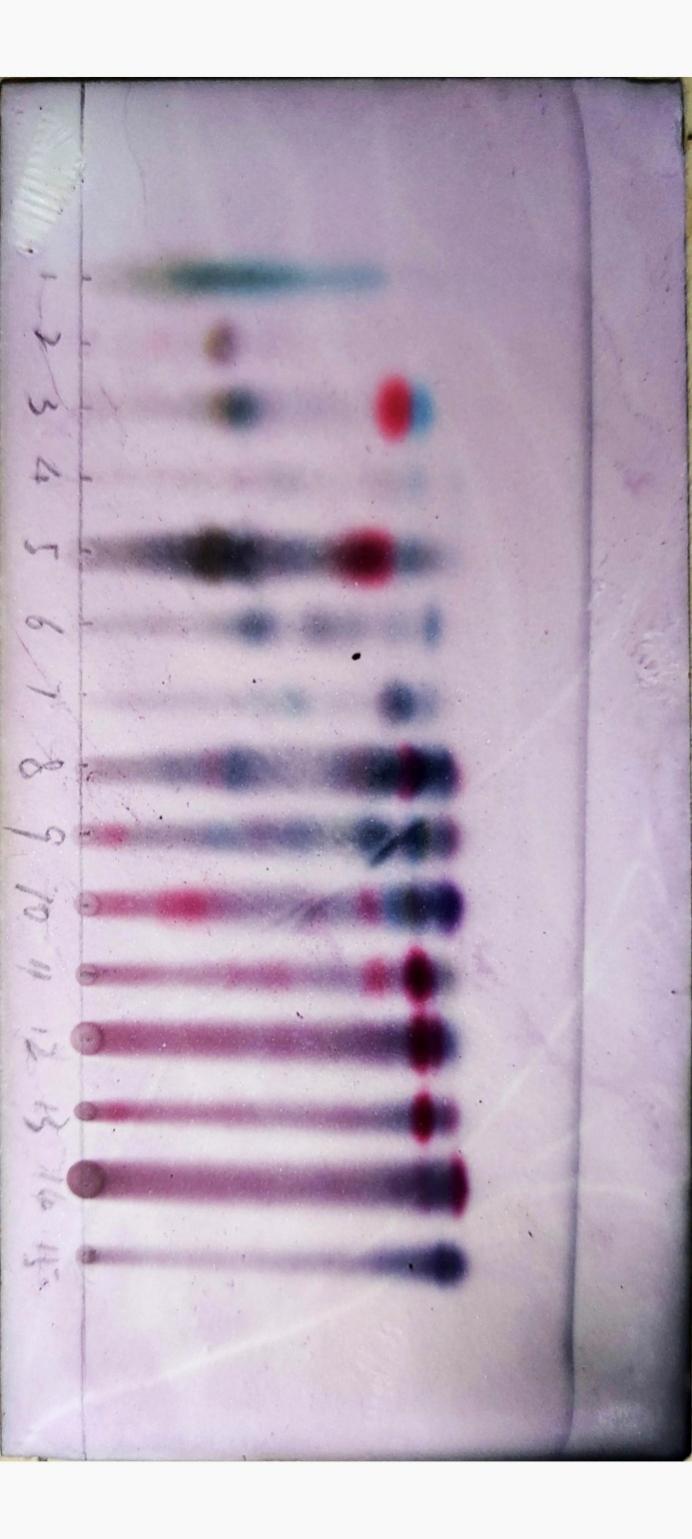


# Calculation details.

All the calculation jobs were performed using Gaussian 16 package [1]. Conformational analyses were conducted by MMFF94s force field. The conformers with distribution higher than 1% were further optimized by the DFT method at the B3LYP/6-31G(d) level. The theoretical calculation of ECD was performed using time-dependent DFT (TD-DFT) at B3LYP/6-31G(d,p) level in MeOH with IEFPCM model (Supplementary materials). The calculated ECD curves were generated using SpecDis 1.71 [2-3] and plotted in the Microsoft Office Excel 2016 program.

## 3.1 ECD calculation of 1.

Conformation search of **1** at MMFF94s force field gave eleven conformers **1a**-**1k**. These conformers were optimized at B3LYP/6-31G(d) level, and then calculated the ECD at B3LYP/6-31G(d,p) level.

| Conformers | Gibbs free energies | Population (%) |
| --- | --- | --- |
| **1a** | -842.793072 | 1.18 |
| **1b** | -842.794679 | 6.47 |
| **1c** | -842.795503 | 15.48 |
| **1d** | -842.794285 | 4.26 |
| **1e** | -842.794315 | 4.40 |
| **1f** | -842.793730 | 2.37 |
| **1j** | -842.794327 | 4.46 |
| **1h** | -842.793578 | 2.02 |
| **1i** | -842.794319 | 4.42 |
| **1j** | -842.796327 | 37.06 |
| **1k** | -842.795586 | 16.90 |

Standard orientation of optimized **1** at B3LYP/6-31G(d) leve.

**1a**

| C | -0.02702 | 0.99302 | -0.25999 |
| --- | --- | --- | --- |
| C | -0.03059 | 0.65628 | 1.28153 |
| C | 0.83072 | -0.61771 | 1.40813 |
| C | 1.63435 | -0.63910 | 0.12994 |
| C | 1.18264 | 0.24532 | -0.76938 |
| C | -1.35095 | 0.52860 | -0.97646 |
| C | -1.66871 | -0.93327 | -0.81653 |
| C | -2.76752 | -1.48667 | -0.27683 |
| C | -2.92599 | -2.98871 | -0.23668 |
| C | -3.92955 | -0.72521 | 0.31347 |
| O | 0.61005 | 1.66200 | 2.05591 |
| C | 0.10380 | 2.48386 | -0.54244 |
| O | 0.83213 | 2.99520 | -1.35918 |
| O | -0.77044 | 3.22787 | 0.20380 |
| C | 2.76325 | -1.57089 | -0.04744 |
| O | 3.11808 | -2.38175 | 0.78627 |
| O | 3.38331 | -1.44601 | -1.25044 |
| H | -1.05708 | 0.49445 | 1.63505 |
| H | 0.21250 | -1.51857 | 1.49860 |
| H | 1.45797 | -0.55936 | 2.30269 |
| H | 1.60229 | 0.41832 | -1.75347 |
| H | -2.16549 | 1.15744 | -0.60661 |
| H | -1.23748 | 0.75654 | -2.04658 |
| H | -0.91222 | -1.60945 | -1.21383 |
| H | -2.06512 | -3.50379 | -0.67370 |
| H | -3.04910 | -3.34493 | 0.79558 |
| H | -3.82574 | -3.30465 | -0.78295 |
| H | -3.79728 | 0.35898 | 0.29869 |
| H | -4.85614 | -0.95654 | -0.22990 |
| H | -4.09924 | -1.02915 | 1.35543 |
| H | 0.05404 | 2.45651 | 2.00580 |
| H | -0.63896 | 4.15390 | -0.07711 |
| H | 4.10539 | -2.10122 | -1.24249 |

**1b**

| C | 0.00684 | 1.01376 | -0.19489 |
| --- | --- | --- | --- |
| C | -0.01944 | 0.62075 | 1.33464 |
| C | 0.77065 | -0.70264 | 1.41010 |
| C | 1.58867 | -0.70699 | 0.14089 |
| C | 1.18555 | 0.23103 | -0.72754 |
| C | -1.33460 | 0.62732 | -0.93148 |
| C | -1.71497 | -0.82390 | -0.82425 |
| C | -2.83596 | -1.34777 | -0.30092 |
| C | -3.06094 | -2.84168 | -0.31733 |
| C | -3.96031 | -0.55844 | 0.32436 |
| O | 0.67100 | 1.55165 | 2.14940 |
| C | 0.12630 | 2.51840 | -0.38476 |
| O | -0.36425 | 3.35511 | 0.35104 |
| O | 0.77491 | 2.86006 | -1.51849 |
| C | 2.67129 | -1.68531 | -0.07129 |
| O | 2.96952 | -2.55880 | 0.71985 |
| O | 3.31970 | -1.52608 | -1.25623 |
| H | -1.05666 | 0.50024 | 1.67538 |
| H | 0.10673 | -1.57415 | 1.44837 |
| H | 1.38719 | -0.71904 | 2.31356 |
| H | 1.61597 | 0.40625 | -1.70669 |
| H | -2.12079 | 1.27715 | -0.53531 |
| H | -1.21058 | 0.88991 | -1.99191 |
| H | -0.99009 | -1.51696 | -1.25037 |
| H | -2.22639 | -3.37739 | -0.77989 |
| H | -3.19389 | -3.23114 | 0.70158 |
| H | -3.97708 | -3.09609 | -0.86843 |
| H | -3.77933 | 0.51835 | 0.35217 |
| H | -4.89817 | -0.72646 | -0.22291 |
| H | -4.13964 | -0.89537 | 1.35441 |
| H | 0.21987 | 2.40715 | 2.03905 |
| H | 0.75364 | 3.83551 | -1.56354 |
| H | 4.00373 | -2.22073 | -1.27509 |

**1c**

| C | 0.00024 | 0.64504 | -0.14359 |
| --- | --- | --- | --- |
| C | 0.09026 | 0.00421 | 1.29131 |
| C | 1.27221 | -0.98203 | 1.18807 |
| C | 2.08867 | -0.43609 | 0.04003 |
| C | 1.40855 | 0.47127 | -0.67466 |
| C | -0.99580 | -0.15361 | -1.07267 |
| C | -2.43616 | -0.11367 | -0.63769 |
| C | -3.23883 | -1.14855 | -0.33889 |
| C | -4.67790 | -0.91125 | 0.05456 |
| C | -2.83534 | -2.60261 | -0.36739 |
| O | 0.43887 | 0.94607 | 2.28991 |
| C | -0.46673 | 2.09045 | -0.11332 |
| O | -1.12832 | 2.60736 | 0.76790 |
| O | -0.12387 | 2.76787 | -1.23192 |
| C | 3.45370 | -0.92029 | -0.23321 |
| O | 4.00803 | -1.79805 | 0.39941 |
| O | 4.05154 | -0.28975 | -1.27936 |
| H | -0.85413 | -0.49649 | 1.54078 |
| H | 0.93998 | -2.00784 | 0.98065 |
| H | 1.82475 | -1.00753 | 2.13142 |
| H | 1.77729 | 0.98946 | -1.55219 |
| H | -0.90183 | 0.26663 | -2.08337 |
| H | -0.62132 | -1.17930 | -1.12938 |
| H | -2.87130 | 0.88384 | -0.57446 |
| H | -4.92928 | 0.15364 | 0.06661 |
| H | -5.36484 | -1.41682 | -0.63852 |
| H | -4.88580 | -1.32193 | 1.05234 |
| H | -1.78727 | -2.76333 | -0.63001 |
| H | -3.01179 | -3.06955 | 0.61113 |
| H | -3.44911 | -3.15798 | -1.08995 |
| H | -0.25672 | 1.62738 | 2.28051 |
| H | -0.49227 | 3.66675 | -1.13212 |
| H | 4.93638 | -0.69222 | -1.35613 |

**1d**

| C | 0.03793 | 0.97249 | -0.20241 |
| --- | --- | --- | --- |
| C | -1.18275 | 1.42221 | -1.10425 |
| C | -2.03273 | 0.13759 | -1.26012 |
| C | -1.65522 | -0.67413 | -0.04236 |
| C | -0.54583 | -0.20520 | 0.54941 |
| C | 1.24666 | 0.53223 | -1.08766 |
| C | 2.42407 | 0.01577 | -0.30298 |
| C | 2.89003 | -1.24153 | -0.23207 |
| C | 4.10252 | -1.56346 | 0.60959 |
| C | 2.29847 | -2.42960 | -0.95064 |
| O | -1.92276 | 2.48357 | -0.52212 |
| C | 0.38951 | 2.10623 | 0.75106 |
| O | -0.07850 | 2.23332 | 1.86325 |
| O | 1.25498 | 2.99954 | 0.22181 |
| C | -2.44386 | -1.84889 | 0.37306 |
| O | -3.43426 | -2.24726 | -0.20902 |
| O | -1.95712 | -2.46022 | 1.48453 |
| H | -0.83114 | 1.81704 | -2.06036 |
| H | -1.79324 | -0.40552 | -2.18444 |
| H | -3.09860 | 0.38214 | -1.30691 |
| H | -0.08113 | -0.62578 | 1.43270 |
| H | 0.88110 | -0.21657 | -1.79704 |
| H | 1.55841 | 1.40436 | -1.67535 |
| H | 2.95169 | 0.77940 | 0.26928 |
| H | 4.49879 | -0.67648 | 1.11345 |
| H | 3.86323 | -2.31347 | 1.37635 |
| H | 4.90585 | -1.99379 | -0.00475 |
| H | 1.41904 | -2.18442 | -1.55006 |
| H | 3.04331 | -2.88940 | -1.61479 |
| H | 2.00417 | -3.20569 | -0.23099 |
| H | -2.18346 | 2.21238 | 0.37504 |
| H | 1.35046 | 3.70811 | 0.88645 |
| H | -2.56013 | -3.20625 | 1.65990 |

**1e**

| C | 1.15108 | -0.36806 | 0.00566 |
| --- | --- | --- | --- |
| C | 1.22773 | 0.29909 | -1.42278 |
| C | -0.02452 | 1.19773 | -1.49602 |
| C | -0.39674 | 1.41053 | -0.04855 |
| C | 0.25733 | 0.57749 | 0.77235 |
| C | 0.53805 | -1.82718 | -0.04189 |
| C | -0.79263 | -1.94047 | -0.73563 |
| C | -2.00734 | -2.04051 | -0.17135 |
| C | -3.24486 | -2.16278 | -1.02813 |
| C | -2.27270 | -2.04042 | 1.31387 |
| O | 2.34754 | 1.15426 | -1.56381 |
| C | 2.53159 | -0.55269 | 0.61523 |
| O | 3.55198 | -0.76612 | -0.01341 |
| O | 2.51803 | -0.53989 | 1.96590 |
| C | -1.40297 | 2.41238 | 0.34565 |
| O | -2.00515 | 3.12373 | -0.43494 |
| O | -1.61166 | 2.47047 | 1.68913 |
| H | 1.23257 | -0.47269 | -2.20557 |
| H | -0.84594 | 0.71752 | -2.04089 |
| H | 0.21414 | 2.12980 | -2.01643 |
| H | 0.14543 | 0.54094 | 1.84952 |
| H | 1.26985 | -2.45771 | -0.56444 |
| H | 0.48038 | -2.19590 | 0.98697 |
| H | -0.74612 | -1.94813 | -1.82461 |
| H | -3.00743 | -2.15121 | -2.09636 |
| H | -3.78540 | -3.09404 | -0.80861 |
| H | -3.94608 | -1.34136 | -0.82604 |
| H | -1.36360 | -2.03116 | 1.91937 |
| H | -2.86710 | -1.16088 | 1.59637 |
| H | -2.86226 | -2.92158 | 1.60101 |
| H | 3.13800 | 0.60111 | -1.43287 |
| H | 3.43652 | -0.71047 | 2.25081 |
| H | -2.28308 | 3.16568 | 1.81827 |

**1f**

| C | 0.01036 | 0.55111 | -0.10577 |
| --- | --- | --- | --- |
| C | -0.92935 | 1.59042 | -0.83313 |
| C | -2.22859 | 0.80759 | -1.11583 |
| C | -2.17143 | -0.34678 | -0.14407 |
| C | -0.96574 | -0.47110 | 0.42735 |
| C | 1.00741 | -0.08303 | -1.14211 |
| C | 1.86523 | -1.18978 | -0.59203 |
| C | 3.19219 | -1.18206 | -0.38696 |
| C | 3.89036 | -2.40384 | 0.16130 |
| C | 4.10842 | -0.01618 | -0.67118 |
| O | -1.27553 | 2.68655 | 0.00060 |
| C | 0.82013 | 1.18059 | 1.01622 |
| O | 0.93381 | 0.76562 | 2.14364 |
| O | 1.47408 | 2.30944 | 0.59581 |
| C | -3.34555 | -1.20778 | 0.08593 |
| O | -4.41279 | -1.07342 | -0.48124 |
| O | -3.12647 | -2.18762 | 1.00163 |
| H | -0.45486 | 1.94915 | -1.75692 |
| H | -2.27617 | 0.45293 | -2.15345 |
| H | -3.09837 | 1.45223 | -0.95816 |
| H | -0.69207 | -1.21174 | 1.16856 |
| H | 0.39205 | -0.47647 | -1.96304 |
| H | 1.61286 | 0.72545 | -1.56167 |
| H | 1.32540 | -2.10191 | -0.33674 |
| H | 3.19377 | -3.22892 | 0.33833 |
| H | 4.66870 | -2.75655 | -0.52991 |
| H | 4.39629 | -2.17548 | 1.10955 |
| H | 3.58233 | 0.88793 | -0.98514 |
| H | 4.69465 | 0.23305 | 0.22381 |
| H | 4.83559 | -0.27637 | -1.45297 |
| H | -0.46489 | 3.20277 | 0.14080 |
| H | 1.98671 | 2.62080 | 1.36640 |
| H | -3.96745 | -2.67650 | 1.06895 |

**1g**

| C | -0.02547 | 1.02806 | -0.19986 |
| --- | --- | --- | --- |
| C | -0.00548 | 0.60930 | 1.32286 |
| C | 0.82894 | -0.68970 | 1.35983 |
| C | 1.61512 | -0.64992 | 0.06903 |
| C | 1.16081 | 0.28888 | -0.77205 |
| C | -1.37235 | 0.61504 | -0.91188 |
| C | -1.70587 | -0.84903 | -0.82486 |
| C | -2.79630 | -1.41729 | -0.28355 |
| C | -2.97794 | -2.91658 | -0.32751 |
| C | -3.92620 | -0.67547 | 0.38819 |
| O | 0.67224 | 1.54712 | 2.14017 |
| C | 0.04628 | 2.53871 | -0.36702 |
| O | -0.44783 | 3.34859 | 0.39624 |
| O | 0.65332 | 2.91645 | -1.51133 |
| C | 2.73936 | -1.54557 | -0.27509 |
| O | 3.37916 | -1.51967 | -1.30807 |
| O | 2.99623 | -2.43965 | 0.71657 |
| H | -1.03057 | 0.44886 | 1.68367 |
| H | 0.19206 | -1.58116 | 1.40320 |
| H | 1.46350 | -0.69361 | 2.25095 |
| H | 1.56940 | 0.48267 | -1.75744 |
| H | -2.16791 | 1.23299 | -0.48457 |
| H | -1.28173 | 0.90089 | -1.96953 |
| H | -0.97277 | -1.51053 | -1.28565 |
| H | -2.14059 | -3.41697 | -0.82343 |
| H | -3.07335 | -3.33199 | 0.68542 |
| H | -3.90018 | -3.18610 | -0.86095 |
| H | -3.77549 | 0.40526 | 0.43584 |
| H | -4.87287 | -0.85877 | -0.13862 |
| H | -4.06879 | -1.04005 | 1.41459 |
| H | 0.19661 | 2.39162 | 2.04839 |
| H | 0.60469 | 3.89152 | -1.54034 |
| H | 3.74772 | -2.97585 | 0.40133 |

**1h**

| C | 0.03368 | 0.58403 | -0.12888 |
| --- | --- | --- | --- |
| C | -0.93487 | 1.55322 | -0.91499 |
| C | -2.15787 | 0.66831 | -1.25629 |
| C | -2.12512 | -0.38394 | -0.17077 |
| C | -0.94255 | -0.41104 | 0.46393 |
| C | 1.03651 | -0.10852 | -1.10730 |
| C | 1.87116 | -1.18606 | -0.46666 |
| C | 3.20249 | -1.21466 | -0.29358 |
| C | 3.86297 | -2.40561 | 0.36048 |
| C | 4.15935 | -0.12822 | -0.71955 |
| O | -1.30008 | 2.68488 | -0.13921 |
| C | 0.75315 | 1.36583 | 0.96132 |
| O | 0.42002 | 1.38797 | 2.12710 |
| O | 1.79727 | 2.08347 | 0.49023 |
| C | -3.28545 | -1.25532 | 0.09269 |
| O | -4.33492 | -1.19846 | -0.51849 |
| O | -3.07414 | -2.14482 | 1.09748 |
| H | -0.44389 | 1.96475 | -1.79981 |
| H | -2.07104 | 0.21103 | -2.25112 |
| H | -3.07872 | 1.26003 | -1.26094 |
| H | -0.68748 | -1.07301 | 1.28247 |
| H | 0.43864 | -0.55217 | -1.91607 |
| H | 1.65810 | 0.66675 | -1.56111 |
| H | 1.30538 | -2.04952 | -0.11443 |
| H | 3.13704 | -3.17427 | 0.64311 |
| H | 4.60136 | -2.86495 | -0.31160 |
| H | 4.41121 | -2.10513 | 1.26425 |
| H | 3.66368 | 0.75545 | -1.12437 |
| H | 4.77177 | 0.19564 | 0.13303 |
| H | 4.86010 | -0.50832 | -1.47604 |
| H | -1.71029 | 2.36439 | 0.68246 |
| H | 2.14135 | 2.58870 | 1.25094 |
| H | -3.90312 | -2.65170 | 1.17993 |

**1i**

| C | -0.04458 | 0.97572 | -0.15295 |
| --- | --- | --- | --- |
| C | -1.26479 | 1.35734 | -1.07705 |
| C | -2.08334 | 0.05434 | -1.19062 |
| C | -1.63866 | -0.75409 | 0.00531 |
| C | -0.54705 | -0.24047 | 0.58843 |
| C | 1.20731 | 0.61928 | -1.03581 |
| C | 2.39049 | 0.10530 | -0.26164 |
| C | 2.94847 | -1.11495 | -0.31303 |
| C | 4.15010 | -1.44351 | 0.54126 |
| C | 2.47710 | -2.25220 | -1.18581 |
| O | -2.11422 | 2.32882 | -0.48482 |
| C | 0.34541 | 2.09270 | 0.80131 |
| O | 0.59637 | 1.97828 | 1.97731 |
| O | 0.44266 | 3.29923 | 0.16080 |
| C | -2.35952 | -1.97426 | 0.40971 |
| O | -3.32312 | -2.42548 | -0.17935 |
| O | -1.84195 | -2.56691 | 1.51770 |
| H | -0.90654 | 1.70807 | -2.05485 |
| H | -1.88034 | -0.47842 | -2.12866 |
| H | -3.15350 | 0.28073 | -1.17712 |
| H | -0.04517 | -0.64003 | 1.46046 |
| H | 0.87493 | -0.10601 | -1.78434 |
| H | 1.48894 | 1.53096 | -1.58096 |
| H | 2.83648 | 0.82687 | 0.42380 |
| H | 4.45755 | -0.59494 | 1.15995 |
| H | 3.93941 | -2.29198 | 1.20696 |
| H | 5.00621 | -1.74160 | -0.08018 |
| H | 1.59776 | -2.00934 | -1.78631 |
| H | 3.27577 | -2.57000 | -1.87024 |
| H | 2.22868 | -3.12814 | -0.57139 |
| H | -1.61175 | 3.15912 | -0.44864 |
| H | 0.73120 | 3.93888 | 0.84001 |
| H | -2.40322 | -3.34678 | 1.68371 |

**1j**

| C | -0.06113 | 0.78341 | -0.32004 |
| --- | --- | --- | --- |
| C | -0.09786 | 0.42782 | 1.20816 |
| C | 1.33653 | -0.06390 | 1.52706 |
| C | 1.76280 | -0.63495 | 0.19437 |
| C | 0.99690 | -0.18783 | -0.81554 |
| C | -1.45636 | 0.68067 | -1.01044 |
| C | -2.06282 | -0.69734 | -0.98260 |
| C | -3.09005 | -1.12453 | -0.22992 |
| C | -3.61433 | -2.53359 | -0.37319 |
| C | -3.81814 | -0.28023 | 0.78967 |
| O | -0.63833 | 1.41168 | 2.05364 |
| C | 0.49376 | 2.19332 | -0.53872 |
| O | 0.78262 | 2.99691 | 0.33044 |
| O | 0.66429 | 2.48129 | -1.84742 |
| C | 2.90319 | -1.56426 | 0.07207 |
| O | 3.58833 | -1.93512 | 1.00452 |
| O | 3.12451 | -1.97822 | -1.20413 |
| H | -0.76646 | -0.43445 | 1.29149 |
| H | 1.36430 | -0.79784 | 2.33767 |
| H | 1.98510 | 0.77114 | 1.82524 |
| H | 1.13040 | -0.43839 | -1.86236 |
| H | -2.11310 | 1.41029 | -0.52587 |
| H | -1.34625 | 1.00075 | -2.05356 |
| H | -1.61467 | -1.41184 | -1.67431 |
| H | -3.07075 | -3.09887 | -1.13668 |
| H | -3.54021 | -3.08109 | 0.57650 |
| H | -4.67900 | -2.52999 | -0.64616 |
| H | -3.30139 | 0.65065 | 1.03314 |
| H | -4.83158 | -0.03524 | 0.44119 |
| H | -3.94020 | -0.83984 | 1.72621 |
| H | -0.12368 | 2.22344 | 1.88761 |
| H | 1.02988 | 3.38615 | -1.88562 |
| H | 3.89128 | -2.57873 | -1.15616 |

**1k**

| C | 0.00885 | 0.56200 | -0.11985 |
| --- | --- | --- | --- |
| C | -0.93717 | 1.60298 | -0.84459 |
| C | -2.24204 | 0.82398 | -1.11118 |
| C | -2.17251 | -0.34029 | -0.15272 |
| C | -0.95979 | -0.47209 | 0.40319 |
| C | 1.01456 | -0.05129 | -1.16030 |
| C | 1.88926 | -1.15177 | -0.62452 |
| C | 3.21621 | -1.12288 | -0.41924 |
| C | 3.93724 | -2.34379 | 0.10133 |
| C | 4.11023 | 0.06544 | -0.68091 |
| O | -1.26787 | 2.70591 | -0.01913 |
| C | 0.84078 | 1.25798 | 0.94428 |
| O | 1.42078 | 2.31405 | 0.76515 |
| O | 0.91162 | 0.58971 | 2.11079 |
| C | -3.33914 | -1.21197 | 0.07499 |
| O | -4.40931 | -1.08301 | -0.48751 |
| O | -3.10996 | -2.19652 | 0.98439 |
| H | -0.46718 | 1.94948 | -1.77540 |
| H | -2.30873 | 0.47688 | -2.15023 |
| H | -3.10752 | 1.46924 | -0.93384 |
| H | -0.68179 | -1.23661 | 1.11821 |
| H | 0.40632 | -0.44190 | -1.98775 |
| H | 1.60790 | 0.77427 | -1.56385 |
| H | 1.36617 | -2.07890 | -0.38843 |
| H | 3.25595 | -3.18448 | 0.26469 |
| H | 4.71741 | -2.66902 | -0.60114 |
| H | 4.44646 | -2.12689 | 1.05070 |
| H | 3.56678 | 0.97146 | -0.95641 |
| H | 4.70600 | 0.29761 | 0.21210 |
| H | 4.82923 | -0.16017 | -1.48091 |
| H | -0.43800 | 3.18230 | 0.15657 |
| H | 1.48615 | 1.12010 | 2.69627 |
| H | -3.94611 | -2.69426 | 1.04696 |

3.2 ECD calculation of 2.

Conformation search of **2** at MMFF94s force field gave nine conformers **2a**-**2d**. These conformers were optimized at B3LYP/6-31G(d) level, and then calculated the ECD at B3LYP/6-31G(d,p) level.

| Conformers | Gibbs free energies | Population (%) |
| --- | --- | --- |
| **2a** | -842.792522 | 1.74 |
| **2b** | -842.793052 | 3.06 |
| **2c** | -842.795151 | 28.22 |
| **2d** | -842.794846 | 20.43 |
| **2e** | -842.793372 | 4.29 |
| **2f** | -842.793115 | 3.27 |
| **2g** | -842.795355 | 35.02 |
| **2h** | -842.792642 | 1.98 |
| **2i** | -842.792651 | 2.00 |

Standard orientation of optimized **2** at B3LYP/6-31G(d) level.

**2a**

| C | -0.01127 | 0.00893 | -0.01714 |
| --- | --- | --- | --- |
| C | 0.46335 | 1.38650 | 0.54739 |
| C | 1.97212 | 1.19691 | 0.84044 |
| C | 2.35920 | 0.05741 | -0.07302 |
| C | 1.28385 | -0.59100 | -0.54258 |
| C | -1.12118 | 0.09466 | -1.10658 |
| C | -2.40895 | 0.69854 | -0.61073 |
| C | -3.60729 | 0.10744 | -0.47796 |
| C | -4.79737 | 0.89462 | 0.01918 |
| C | -3.90960 | -1.33487 | -0.80614 |
| O | 0.25968 | 2.35689 | -0.48003 |
| C | -0.48229 | -0.87678 | 1.14314 |
| O | -0.52013 | -0.56654 | 2.31298 |
| O | -0.83029 | -2.11638 | 0.71447 |
| C | 3.77041 | -0.26230 | -0.35257 |
| O | 4.71467 | 0.34456 | 0.11519 |
| O | 3.92872 | -1.31570 | -1.19692 |
| H | -0.10133 | 1.65128 | 1.44644 |
| H | 2.14746 | 0.93082 | 1.89079 |
| H | 2.54476 | 2.10970 | 0.64076 |
| H | 1.30367 | -1.46159 | -1.18861 |
| H | -0.71898 | 0.71496 | -1.91429 |
| H | -1.27428 | -0.90949 | -1.50980 |
| H | -2.32983 | 1.75175 | -0.34490 |
| H | -4.53789 | 1.93574 | 0.23453 |
| H | -5.60987 | 0.89228 | -0.72129 |
| H | -5.21143 | 0.44969 | 0.93499 |
| H | -3.02803 | -1.90969 | -1.09577 |
| H | -4.36531 | -1.83673 | 0.05876 |
| H | -4.64443 | -1.39957 | -1.62099 |
| H | 0.54208 | 3.21390 | -0.12600 |
| H | -1.09560 | -2.61165 | 1.51243 |
| H | 4.89190 | -1.42436 | -1.30330 |

**2b**

| C | -0.01765 | 0.00065 | -0.03240 |
| --- | --- | --- | --- |
| C | 0.45376 | 1.37952 | 0.55955 |
| C | 1.94895 | 1.16386 | 0.89225 |
| C | 2.35310 | 0.06337 | -0.06503 |
| C | 1.28395 | -0.57388 | -0.57003 |
| C | -1.13270 | 0.10264 | -1.11206 |
| C | -2.41133 | 0.71253 | -0.59958 |
| C | -3.61234 | 0.12774 | -0.46372 |
| C | -4.79153 | 0.91709 | 0.05515 |
| C | -3.92775 | -1.30813 | -0.80722 |
| O | 0.27524 | 2.44646 | -0.36382 |
| C | -0.47653 | -0.90946 | 1.11282 |
| O | -0.49793 | -0.62063 | 2.28826 |
| O | -0.83543 | -2.13948 | 0.66459 |
| C | 3.76916 | -0.24665 | -0.34046 |
| O | 4.70757 | 0.33741 | 0.16469 |
| O | 3.93469 | -1.26107 | -1.22993 |
| H | -0.13620 | 1.64930 | 1.43442 |
| H | 2.08534 | 0.83962 | 1.93144 |
| H | 2.52445 | 2.08652 | 0.76600 |
| H | 1.31818 | -1.41833 | -1.24992 |
| H | -0.73984 | 0.72884 | -1.92229 |
| H | -1.29566 | -0.89366 | -1.53062 |
| H | -2.32077 | 1.76124 | -0.32047 |
| H | -4.52258 | 1.95358 | 0.28016 |
| H | -5.61155 | 0.92849 | -0.67683 |
| H | -5.19858 | 0.46367 | 0.96983 |
| H | -3.05355 | -1.88572 | -1.11335 |
| H | -4.37779 | -1.81762 | 0.05606 |
| H | -4.67172 | -1.35712 | -1.61477 |
| H | 0.88814 | 2.30493 | -1.10430 |
| H | -1.09438 | -2.64910 | 1.45583 |
| H | 4.89871 | -1.37182 | -1.32712 |

**2c**

| C | -0.03816 | 0.51913 | -0.09564 |
| --- | --- | --- | --- |
| C | -0.13555 | -0.31080 | 1.24252 |
| C | -1.64209 | -0.57344 | 1.44483 |
| C | -2.23591 | -0.34691 | 0.07531 |
| C | -1.37215 | 0.24445 | -0.76187 |
| C | 1.15933 | 0.10217 | -1.00445 |
| C | 2.50635 | 0.25138 | -0.34077 |
| C | 3.52653 | -0.62593 | -0.33436 |
| C | 4.82574 | -0.28638 | 0.35840 |
| C | 3.51308 | -1.97457 | -1.01424 |
| O | 0.49405 | -1.58058 | 1.12811 |
| C | 0.07669 | 2.01613 | 0.21102 |
| O | 0.37382 | 2.51061 | 1.27650 |
| O | -0.17725 | 2.77318 | -0.88679 |
| C | -3.62737 | -0.72662 | -0.23142 |
| O | -4.39912 | -1.20843 | 0.57477 |
| O | -3.98196 | -0.48440 | -1.52189 |
| H | 0.29712 | 0.26236 | 2.06820 |
| H | -2.08221 | 0.11147 | 2.18075 |
| H | -1.80298 | -1.59032 | 1.81472 |
| H | -1.58295 | 0.52245 | -1.78848 |
| H | 0.98009 | -0.92821 | -1.31850 |
| H | 1.11970 | 0.72571 | -1.90812 |
| H | 2.66416 | 1.19815 | 0.17779 |
| H | 4.79134 | 0.69784 | 0.83535 |
| H | 5.07112 | -1.03154 | 1.12757 |
| H | 5.66196 | -0.29066 | -0.35455 |
| H | 2.53874 | -2.24670 | -1.42566 |
| H | 4.24080 | -1.99263 | -1.83737 |
| H | 3.81834 | -2.76482 | -0.31550 |
| H | 1.45128 | -1.41704 | 1.08406 |
| H | -0.07122 | 3.70206 | -0.60579 |
| H | -4.91048 | -0.77280 | -1.59611 |

**2d**

| C | -0.02376 | -0.52435 | 0.17937 |
| --- | --- | --- | --- |
| C | -0.93204 | -1.74311 | -0.25034 |
| C | -2.18928 | -1.08500 | -0.84268 |
| C | -2.24195 | 0.22388 | -0.08703 |
| C | -1.06975 | 0.51777 | 0.49888 |
| C | 0.90447 | -0.06522 | -0.99890 |
| C | 1.76206 | 1.12508 | -0.66445 |
| C | 3.10003 | 1.17731 | -0.56212 |
| C | 3.79434 | 2.47752 | -0.23256 |
| C | 4.03205 | 0.00690 | -0.76331 |
| O | -0.34391 | -2.64580 | -1.15901 |
| C | 0.83269 | -0.88362 | 1.37731 |
| O | 0.89123 | -0.30594 | 2.43681 |
| O | 1.56659 | -2.01134 | 1.12609 |
| C | -3.47004 | 1.03896 | -0.04634 |
| O | -4.51209 | 0.73434 | -0.59369 |
| O | -3.33245 | 2.18393 | 0.67223 |
| H | -1.22598 | -2.26729 | 0.67293 |
| H | -3.08966 | -1.69167 | -0.71083 |
| H | -2.06430 | -0.92722 | -1.92234 |
| H | -0.86796 | 1.39488 | 1.10190 |
| H | 1.49883 | -0.92395 | -1.31525 |
| H | 0.24616 | 0.18048 | -1.84194 |
| H | 1.21323 | 2.05142 | -0.49066 |
| H | 3.08442 | 3.30074 | -0.10693 |
| H | 4.37988 | 2.38830 | 0.69306 |
| H | 4.50420 | 2.75699 | -1.02380 |
| H | 3.51342 | -0.93951 | -0.92887 |
| H | 4.69727 | 0.18495 | -1.61971 |
| H | 4.68390 | -0.11700 | 0.11238 |
| H | 0.39606 | -3.06983 | -0.69325 |
| H | 2.08321 | -2.18567 | 1.93568 |
| H | -4.20045 | 2.62626 | 0.63109 |

**2e**

| C | -0.00454 | 0.54954 | -0.19007 |
| --- | --- | --- | --- |
| C | -0.11335 | -0.16855 | 1.22321 |
| C | -1.61653 | -0.45029 | 1.42805 |
| C | -2.19151 | -0.35827 | 0.03704 |
| C | -1.33203 | 0.18662 | -0.83429 |
| C | 1.19149 | 0.07425 | -1.05614 |
| C | 2.54223 | 0.25850 | -0.40914 |
| C | 3.51619 | -0.65636 | -0.24688 |
| C | 4.83592 | -0.26689 | 0.37834 |
| C | 3.43541 | -2.10046 | -0.68564 |
| O | 0.54730 | -1.42318 | 1.22282 |
| C | 0.04299 | 2.07475 | -0.04317 |
| O | 0.70570 | 2.83330 | -0.71718 |
| O | -0.78185 | 2.52158 | 0.93723 |
| C | -3.56534 | -0.80612 | -0.25876 |
| O | -4.33441 | -1.24931 | 0.57154 |
| O | -3.90525 | -0.67556 | -1.56891 |
| H | 0.29009 | 0.47402 | 2.01512 |
| H | -2.08056 | 0.29084 | 2.08902 |
| H | -1.75946 | -1.43450 | 1.88378 |
| H | -1.53260 | 0.36553 | -1.88589 |
| H | 1.01392 | -0.96944 | -1.32200 |
| H | 1.16612 | 0.65696 | -1.98579 |
| H | 2.74992 | 1.27549 | -0.07807 |
| H | 4.85485 | 0.78708 | 0.67201 |
| H | 5.05018 | -0.87570 | 1.26762 |
| H | 5.66556 | -0.43903 | -0.32148 |
| H | 2.43905 | -2.40211 | -1.01562 |
| H | 4.13463 | -2.28664 | -1.51263 |
| H | 3.73718 | -2.77139 | 0.12976 |
| H | 1.49958 | -1.24104 | 1.14582 |
| H | -0.70343 | 3.49451 | 0.93420 |
| H | -4.82259 | -0.99951 | -1.63516 |

**2f**

| C | -1.20913 | -0.03831 | -0.16504 |
| --- | --- | --- | --- |
| C | -1.23461 | -0.59698 | 1.30757 |
| C | 0.20002 | -1.09171 | 1.56700 |
| C | 0.69515 | -1.38661 | 0.17017 |
| C | -0.08261 | -0.83470 | -0.77399 |
| C | -0.94306 | 1.51633 | -0.24879 |
| C | 0.30740 | 2.00153 | 0.43276 |
| C | 1.46904 | 2.35662 | -0.13971 |
| C | 2.61997 | 2.85010 | 0.70491 |
| C | 1.75759 | 2.30672 | -1.62003 |
| O | -1.65321 | 0.32310 | 2.29474 |
| C | -2.52880 | -0.27360 | -0.87918 |
| O | -2.68104 | -0.66048 | -2.01497 |
| O | -3.58624 | 0.05099 | -0.07717 |
| C | 1.91339 | -2.18454 | -0.05932 |
| O | 2.58889 | -2.67429 | 0.82510 |
| O | 2.22374 | -2.32853 | -1.37507 |
| H | -1.89951 | -1.47375 | 1.30830 |
| H | 0.23259 | -1.96631 | 2.22362 |
| H | 0.79788 | -0.30601 | 2.04368 |
| H | 0.07138 | -0.91007 | -1.84412 |
| H | -0.95011 | 1.78618 | -1.31023 |
| H | -1.80776 | 2.00907 | 0.21034 |
| H | 0.22776 | 2.07249 | 1.51568 |
| H | 2.36927 | 2.86352 | 1.77002 |
| H | 3.50776 | 2.21617 | 0.57143 |
| H | 2.91769 | 3.86661 | 0.41136 |
| H | 0.89773 | 2.00477 | -2.22256 |
| H | 2.09617 | 3.28721 | -1.98198 |
| H | 2.57238 | 1.59973 | -1.82848 |
| H | -2.59684 | 0.49103 | 2.13759 |
| H | -4.39092 | -0.10139 | -0.60833 |
| H | 3.03122 | -2.87471 | -1.39548 |

**2g**

| C | -0.10207 | 0.98595 | 0.07908 |
| --- | --- | --- | --- |
| C | 1.05846 | 1.72845 | -0.69097 |
| C | 2.06270 | 0.61328 | -1.02719 |
| C | 1.81676 | -0.37962 | 0.08658 |
| C | 0.64846 | -0.16171 | 0.71177 |
| C | -1.19843 | 0.45814 | -0.91303 |
| C | -2.28679 | -0.33747 | -0.24468 |
| C | -2.58068 | -1.63812 | -0.40226 |
| C | -3.73189 | -2.26062 | 0.35217 |
| C | -1.84332 | -2.59250 | -1.30944 |
| O | 0.66406 | 2.43173 | -1.84716 |
| C | -0.75179 | 1.90049 | 1.09891 |
| O | -0.96036 | 1.66577 | 2.26641 |
| O | -1.10534 | 3.09165 | 0.52651 |
| C | 2.78450 | -1.45055 | 0.38705 |
| O | 3.82815 | -1.61831 | -0.21403 |
| O | 2.40130 | -2.25016 | 1.41667 |
| H | 1.53773 | 2.41257 | 0.02696 |
| H | 3.09617 | 0.96939 | -1.06317 |
| H | 1.83900 | 0.18276 | -2.01252 |
| H | 0.25184 | -0.74157 | 1.53589 |
| H | -1.62817 | 1.32962 | -1.42096 |
| H | -0.68637 | -0.12290 | -1.68360 |
| H | -2.90725 | 0.22923 | 0.45128 |
| H | -4.23697 | -1.53700 | 0.99937 |
| H | -4.47689 | -2.67864 | -0.33941 |
| H | -3.38727 | -3.09535 | 0.97835 |
| H | -1.00392 | -2.13450 | -1.83691 |
| H | -1.45224 | -3.44294 | -0.73446 |
| H | -2.52444 | -3.01406 | -2.06162 |
| H | 0.09735 | 3.16293 | -1.55006 |
| H | -1.51305 | 3.62552 | 1.23472 |
| H | 3.11646 | -2.90470 | 1.52095 |

**2h**

| C | -0.02726 | -0.01428 | -0.05219 |
| --- | --- | --- | --- |
| C | 0.46461 | 1.33911 | 0.57928 |
| C | 1.96146 | 1.09679 | 0.89169 |
| C | 2.34430 | 0.02393 | -0.10801 |
| C | 1.26188 | -0.58190 | -0.62220 |
| C | -1.15435 | 0.13325 | -1.11368 |
| C | -2.41949 | 0.74107 | -0.56633 |
| C | -3.62566 | 0.16600 | -0.43463 |
| C | -4.78932 | 0.95197 | 0.12303 |
| C | -3.96191 | -1.25446 | -0.81975 |
| O | 0.29202 | 2.43711 | -0.30791 |
| C | -0.47892 | -0.95755 | 1.06919 |
| O | -0.46961 | -0.71015 | 2.25445 |
| O | -0.86799 | -2.16399 | 0.58570 |
| C | 3.72325 | -0.36832 | -0.47128 |
| O | 4.02726 | -1.21485 | -1.28805 |
| O | 4.65033 | 0.34139 | 0.22388 |
| H | -0.11369 | 1.58868 | 1.46789 |
| H | 2.10173 | 0.73722 | 1.91871 |
| H | 2.53796 | 2.02214 | 0.79218 |
| H | 1.29227 | -1.40360 | -1.33012 |
| H | -0.76503 | 0.77895 | -1.91026 |
| H | -1.33333 | -0.84794 | -1.56020 |
| H | -2.31363 | 1.77940 | -0.25546 |
| H | -4.50539 | 1.97748 | 0.37807 |
| H | -5.61724 | 0.99660 | -0.59869 |
| H | -5.19164 | 0.47404 | 1.02727 |
| H | -3.09796 | -1.83208 | -1.15345 |
| H | -4.40890 | -1.78577 | 0.03188 |
| H | -4.71485 | -1.26907 | -1.62028 |
| H | 0.88038 | 2.29969 | -1.06891 |
| H | -1.11665 | -2.69899 | 1.36337 |
| H | 5.51841 | 0.01348 | -0.07744 |

**2i**

| C | -0.06402 | 1.06682 | -0.17181 |
| --- | --- | --- | --- |
| C | -0.07143 | 0.67291 | 1.37160 |
| C | -0.99623 | -0.57122 | 1.46500 |
| C | -1.79067 | -0.52065 | 0.18488 |
| C | -1.29498 | 0.36405 | -0.69247 |
| C | 1.24496 | 0.61884 | -0.90368 |
| C | 1.54221 | -0.85698 | -0.82100 |
| C | 2.70318 | -1.44701 | -0.48002 |
| C | 2.83600 | -2.95196 | -0.51294 |
| C | 3.96291 | -0.71702 | -0.07644 |
| O | 1.19944 | 0.50948 | 1.95475 |
| C | -0.21925 | 2.57351 | -0.34231 |
| O | -1.17593 | 3.13132 | -0.83605 |
| O | 0.84528 | 3.25782 | 0.14232 |
| C | -2.96435 | -1.39047 | -0.02123 |
| O | -3.37667 | -2.18724 | 0.79958 |
| O | -3.55492 | -1.22246 | -1.23312 |
| H | -0.53066 | 1.49560 | 1.92805 |
| H | -1.62983 | -0.55501 | 2.35766 |
| H | -0.40096 | -1.49286 | 1.52513 |
| H | -1.70852 | 0.56676 | -1.67384 |
| H | 1.13869 | 0.89948 | -1.96278 |
| H | 2.06563 | 1.21581 | -0.50329 |
| H | 0.71984 | -1.50922 | -1.11521 |
| H | 1.90481 | -3.44000 | -0.81645 |
| H | 3.62491 | -3.25900 | -1.21353 |
| H | 3.12540 | -3.34637 | 0.47081 |
| H | 3.82009 | 0.35638 | 0.06471 |
| H | 4.36681 | -1.12936 | 0.85774 |
| H | 4.74353 | -0.85528 | -0.83732 |
| H | 1.60486 | -0.26872 | 1.53520 |
| H | 0.63950 | 4.20282 | 0.01055 |
| H | -4.31161 | -1.83736 | -1.24482 |

## 3.3 ECD calculation of 3

Conformation search of **3** at MMFF94s force field gave thirteen conformers **3a**-**3m**. These conformers were optimized at B3LYP/6-31G(d) level, and then calculated the ECD at B3LYP/6-31G(d,p) level.

| Conformers | Gibbs free energies | Population (%) |
| --- | --- | --- |
| **3a** | -995.429982 | 7.1 |
| **3b** | -995.429066 | 2.7 |
| **3c** | -995.431455 | 33.6 |
| **3d** | -995.431413 | 32.2 |
| **3e** | -995.429343 | 3.6 |
| **3f** | -995.430429 | 11.3 |
| **3g** | -995.428845 | 6.7 |
| **3h** | -995.429930 | 2.9 |
| **3i** | -995.429131 | 2.1 |
| **3j** | -995.430772 | 16.3 |
| **3k** | -995.430334 | 10.3 |
| **3l** | -995.428884 | 2.2 |
| **3m** | -995.430661 | 14.5 |

Standard orientation of optimized **3** at B3LYP/6-31G(d) level.

**3a**

| C | -0.18158 | -0.00831 | 0.98855 |
| --- | --- | --- | --- |
| C | -0.57810 | -0.10112 | -0.52309 |
| C | 0.42710 | 0.80162 | -1.26865 |
| C | 0.97408 | 1.69318 | -0.17809 |
| C | 0.63030 | 1.26557 | 1.04436 |
| C | 0.65987 | -1.25890 | 1.44435 |
| C | 1.89986 | -1.51493 | 0.63136 |
| C | 2.18774 | -2.59589 | -0.11328 |
| C | 3.50889 | -2.69316 | -0.83985 |
| C | 1.28699 | -3.79238 | -0.30163 |
| C | 1.80139 | 2.87009 | -0.49947 |
| O | 2.09432 | 3.21024 | -1.62960 |
| O | 2.21771 | 3.55346 | 0.59826 |
| O | -1.90266 | 0.46281 | -0.67612 |
| O | -2.57196 | -1.29650 | -1.95132 |
| C | -2.81140 | -0.24836 | -1.39708 |
| C | -4.14409 | 0.46132 | -1.39881 |
| C | -1.39443 | 0.08088 | 1.91305 |
| O | -1.60928 | 0.97071 | 2.70430 |
| O | -2.20510 | -1.00143 | 1.79988 |
| H | -0.59967 | -1.12498 | -0.89272 |
| H | 1.22524 | 0.20856 | -1.73030 |
| H | -0.06356 | 1.36284 | -2.06975 |
| H | 0.90353 | 1.73937 | 1.97954 |
| H | -0.00864 | -2.12247 | 1.43867 |
| H | 0.94681 | -1.08888 | 2.49260 |
| H | 2.65105 | -0.72692 | 0.68038 |
| H | 4.13248 | -1.80838 | -0.67892 |
| H | 4.07559 | -3.57459 | -0.50876 |
| H | 3.35593 | -2.81172 | -1.92149 |
| H | 0.32826 | -3.70744 | 0.21438 |
| H | 1.07845 | -3.95193 | -1.36809 |
| H | 1.78233 | -4.70532 | 0.05680 |
| H | 2.74123 | 4.30300 | 0.25920 |
| H | -4.84371 | -0.07974 | -2.03662 |
| H | -4.53662 | 0.51262 | -0.37763 |
| H | -4.02683 | 1.48967 | -1.75450 |
| H | -2.92578 | -0.86555 | 2.44377 |

**3b**

| C | -0.29408 | -0.68218 | 0.58134 |
| --- | --- | --- | --- |
| C | -0.95757 | -0.04125 | -0.68125 |
| C | -0.02994 | 1.12571 | -1.08002 |
| C | 0.74411 | 1.40167 | 0.18790 |
| C | 0.57836 | 0.43517 | 1.10078 |
| C | 0.56570 | -1.95626 | 0.21564 |
| C | 1.57705 | -1.75850 | -0.88181 |
| C | 2.90103 | -1.56868 | -0.75905 |
| C | 3.77078 | -1.40362 | -1.98251 |
| C | 3.64518 | -1.49815 | 0.55159 |
| C | 1.58198 | 2.60583 | 0.32277 |
| O | 1.69260 | 3.46156 | -0.53414 |
| O | 2.23897 | 2.67647 | 1.51065 |
| O | -2.23610 | 0.50444 | -0.27690 |
| O | -3.23431 | -0.29812 | -2.15409 |
| C | -3.29987 | 0.28462 | -1.09610 |
| C | -4.55332 | 0.86957 | -0.49104 |
| C | -1.31383 | -1.12228 | 1.62844 |
| O | -1.31976 | -0.77862 | 2.78883 |
| O | -2.20392 | -2.01673 | 1.13051 |
| H | -1.13519 | -0.75497 | -1.48471 |
| H | 0.64342 | 0.83977 | -1.89684 |
| H | -0.60592 | 1.98982 | -1.42473 |
| H | 1.02763 | 0.41174 | 2.08601 |
| H | -0.14027 | -2.73961 | -0.08273 |
| H | 1.04662 | -2.29970 | 1.13781 |
| H | 1.16936 | -1.78610 | -1.89282 |
| H | 3.18987 | -1.45127 | -2.90877 |
| H | 4.30122 | -0.44156 | -1.96162 |
| H | 4.54375 | -2.18357 | -2.02412 |
| H | 3.01773 | -1.70616 | 1.42125 |
| H | 4.48153 | -2.21002 | 0.55956 |
| H | 4.08238 | -0.50004 | 0.69087 |
| H | 2.74099 | 3.51200 | 1.48363 |
| H | -5.38490 | 0.74439 | -1.18514 |
| H | -4.77891 | 0.36355 | 0.45369 |
| H | -4.40686 | 1.93044 | -0.26534 |
| H | -2.78585 | -2.26048 | 1.87507 |

**3c**

| C | 0.23363 | -0.02272 | -0.04827 |
| --- | --- | --- | --- |
| C | -1.05214 | -0.55695 | -0.75728 |
| C | -1.86275 | 0.70547 | -1.11675 |
| C | -1.33795 | 1.73641 | -0.14301 |
| C | -0.21321 | 1.32937 | 0.46229 |
| C | 1.39175 | 0.11891 | -1.10119 |
| C | 2.63686 | 0.76941 | -0.56120 |
| C | 3.84786 | 0.21661 | -0.38472 |
| C | 4.99393 | 1.04043 | 0.15299 |
| C | 4.20346 | -1.21683 | -0.69715 |
| C | -2.02966 | 3.02288 | 0.05150 |
| O | -3.04539 | 3.34642 | -0.53375 |
| O | -1.42104 | 3.83129 | 0.95748 |
| O | -1.79551 | -1.33373 | 0.21013 |
| O | -2.38469 | -2.84007 | -1.38440 |
| C | -2.40299 | -2.46757 | -0.23367 |
| C | -3.09184 | -3.17303 | 0.90954 |
| C | 0.69975 | -0.93071 | 1.08380 |
| O | 0.82338 | -0.60799 | 2.24223 |
| O | 1.00527 | -2.17605 | 0.63664 |
| H | -0.83465 | -1.19570 | -1.61284 |
| H | -1.68773 | 1.01112 | -2.15666 |
| H | -2.93830 | 0.53213 | -1.01761 |
| H | 0.34292 | 1.88835 | 1.20406 |
| H | 0.99673 | 0.72630 | -1.92732 |
| H | 1.59847 | -0.87424 | -1.50708 |
| H | 2.52372 | 1.82022 | -0.29302 |
| H | 4.69822 | 2.07522 | 0.35098 |
| H | 5.38219 | 0.61176 | 1.08722 |
| H | 5.83423 | 1.05521 | -0.55521 |
| H | 3.34723 | -1.82518 | -0.99437 |
| H | 4.95224 | -1.26234 | -1.50022 |
| H | 4.66292 | -1.69373 | 0.17931 |
| H | -1.96982 | 4.63602 | 1.00335 |
| H | -3.59070 | -4.06797 | 0.53652 |
| H | -2.35923 | -3.44540 | 1.67635 |
| H | -3.82128 | -2.50559 | 1.37955 |
| H | 1.30644 | -2.67531 | 1.41907 |

**3d**

| C | 0.03457 | -0.60008 | 0.00534 |
| --- | --- | --- | --- |
| C | -1.23990 | -0.13293 | -0.76562 |
| C | -1.03818 | 1.37966 | -0.99162 |
| C | -0.05017 | 1.75649 | 0.08956 |
| C | 0.51438 | 0.67917 | 0.65344 |
| C | 1.10023 | -1.14081 | -1.01510 |
| C | 2.39865 | -1.55740 | -0.37825 |
| C | 3.60286 | -0.97374 | -0.48725 |
| C | 4.80255 | -1.55324 | 0.22435 |
| C | 3.89760 | 0.26569 | -1.29613 |
| C | 0.22417 | 3.16778 | 0.41174 |
| O | -0.30640 | 4.11020 | -0.14463 |
| O | 1.13993 | 3.32801 | 1.40214 |
| O | -2.37180 | -0.34277 | 0.11110 |
| O | -3.74019 | -0.57855 | -1.68168 |
| C | -3.57304 | -0.57100 | -0.48320 |
| C | -4.64026 | -0.80616 | 0.55834 |
| C | -0.27466 | -1.68035 | 1.03619 |
| O | -0.08704 | -1.59265 | 2.22766 |
| O | -0.76063 | -2.80600 | 0.45441 |
| H | -1.41205 | -0.69072 | -1.68605 |
| H | -0.63425 | 1.58384 | -1.99202 |
| H | -1.98288 | 1.92621 | -0.92095 |
| H | 1.25441 | 0.69032 | 1.44303 |
| H | 1.25591 | -0.36523 | -1.77079 |
| H | 0.65134 | -2.00202 | -1.52556 |
| H | 2.33168 | -2.45061 | 0.24442 |
| H | 4.54553 | -2.44801 | 0.79945 |
| H | 5.59275 | -1.82193 | -0.49063 |
| H | 5.24369 | -0.81988 | 0.91352 |
| H | 3.01996 | 0.68165 | -1.79559 |
| H | 4.31722 | 1.05123 | -0.65316 |
| H | 4.65552 | 0.05482 | -2.06310 |
| H | 1.22628 | 4.29123 | 1.52703 |
| H | -5.59671 | -0.98149 | 0.06500 |
| H | -4.37537 | -1.66862 | 1.17862 |
| H | -4.71520 | 0.06116 | 1.22229 |
| H | -0.92160 | -3.43639 | 1.18170 |

**3e**

| C | -0.19778 | -0.04310 | 1.00931 |
| --- | --- | --- | --- |
| C | -0.57119 | -0.09104 | -0.50997 |
| C | 0.42457 | 0.85903 | -1.21063 |
| C | 0.94310 | 1.71998 | -0.07955 |
| C | 0.58928 | 1.24099 | 1.12034 |
| C | 0.66137 | -1.29280 | 1.43362 |
| C | 1.91891 | -1.49501 | 0.63250 |
| C | 2.24017 | -2.54286 | -0.14510 |
| C | 3.57554 | -2.58942 | -0.85055 |
| C | 1.36646 | -3.74882 | -0.39166 |
| C | 1.76696 | 2.93519 | -0.24592 |
| O | 2.20737 | 3.62495 | 0.65150 |
| O | 1.99408 | 3.21479 | -1.55796 |
| O | -1.90589 | 0.44861 | -0.66158 |
| O | -2.50114 | -1.25107 | -2.04876 |
| C | -2.78100 | -0.24422 | -1.43942 |
| C | -4.13432 | 0.42497 | -1.42346 |
| C | -1.42649 | -0.00984 | 1.91677 |
| O | -1.67476 | 0.84991 | 2.73063 |
| O | -2.20989 | -1.10680 | 1.75690 |
| H | -0.56465 | -1.10110 | -0.91648 |
| H | 1.23712 | 0.29668 | -1.68572 |
| H | -0.07420 | 1.43485 | -1.99634 |
| H | 0.85065 | 1.69264 | 2.07018 |
| H | 0.01051 | -2.16842 | 1.38632 |
| H | 0.92885 | -1.15483 | 2.49157 |
| H | 2.65393 | -0.69560 | 0.72362 |
| H | 4.17883 | -1.69934 | -0.64701 |
| H | 4.15354 | -3.47093 | -0.53988 |
| H | 3.44424 | -2.67310 | -1.93835 |
| H | 0.39724 | -3.70017 | 0.10923 |
| H | 1.18012 | -3.87505 | -1.46669 |
| H | 1.87298 | -4.66423 | -0.05586 |
| H | 2.53740 | 4.02499 | -1.56589 |
| H | -4.80145 | -0.08610 | -2.11828 |
| H | -4.55260 | 0.38499 | -0.41196 |
| H | -4.04115 | 1.48082 | -1.69558 |
| H | -2.94298 | -1.00741 | 2.39340 |

**3f**

| C | 0.11833 | 0.25480 | 0.81303 |
| --- | --- | --- | --- |
| C | -0.10374 | 0.51888 | -0.73027 |
| C | 1.29376 | 0.60871 | -1.37302 |
| C | 2.19873 | 0.02070 | -0.31966 |
| C | 1.57637 | -0.14227 | 0.85562 |
| C | -0.83294 | -0.84204 | 1.38184 |
| C | -0.65014 | -2.19434 | 0.74636 |
| C | -1.57734 | -2.96431 | 0.15267 |
| C | -1.20620 | -4.32881 | -0.38025 |
| C | -3.03110 | -2.59964 | -0.02259 |
| C | 3.61483 | -0.26843 | -0.60850 |
| O | 4.14970 | -0.06024 | -1.68030 |
| O | 4.28267 | -0.80212 | 0.44801 |
| O | -0.81743 | 1.74514 | -0.98041 |
| O | -2.80587 | 0.65066 | -1.08321 |
| C | -2.17201 | 1.68131 | -1.09919 |
| C | -2.75364 | 3.06627 | -1.23079 |
| C | -0.14695 | 1.55855 | 1.58200 |
| O | -1.21940 | 1.88711 | 2.03931 |
| O | 0.95168 | 2.34136 | 1.67992 |
| H | -0.68429 | -0.31058 | -1.13621 |
| H | 1.35151 | 0.07548 | -2.32734 |
| H | 1.55562 | 1.65481 | -1.57479 |
| H | 2.04972 | -0.51379 | 1.75883 |
| H | -1.85672 | -0.47750 | 1.28960 |
| H | -0.62981 | -0.92132 | 2.46055 |
| H | 0.36111 | -2.59883 | 0.81198 |
| H | -0.14886 | -4.56136 | -0.21844 |
| H | -1.80508 | -5.11467 | 0.10129 |
| H | -1.41193 | -4.40023 | -1.45731 |
| H | -3.25548 | -1.56359 | 0.23191 |
| H | -3.33909 | -2.75306 | -1.06518 |
| H | -3.66581 | -3.25639 | 0.58943 |
| H | 5.19606 | -0.94386 | 0.13741 |
| H | -3.78612 | 2.99695 | -1.57522 |
| H | -2.73386 | 3.54640 | -0.24570 |
| H | -2.15981 | 3.67873 | -1.91469 |
| H | 0.66422 | 3.15125 | 2.14373 |

**3g**

| C | -0.29777 | -0.74712 | 0.54532 |
| --- | --- | --- | --- |
| C | -0.95386 | -0.00886 | -0.66576 |
| C | -0.02357 | 1.18629 | -0.96772 |
| C | 0.74662 | 1.35922 | 0.32306 |
| C | 0.57117 | 0.32425 | 1.15494 |
| C | 0.56779 | -1.98651 | 0.08387 |
| C | 1.58952 | -1.69871 | -0.98388 |
| C | 2.90830 | -1.49631 | -0.82815 |
| C | 3.79199 | -1.23352 | -2.02426 |
| C | 3.63182 | -1.50195 | 0.49579 |
| C | 1.62447 | 2.50271 | 0.64243 |
| O | 2.29693 | 2.62505 | 1.64704 |
| O | 1.61047 | 3.44174 | -0.34196 |
| O | -2.23441 | 0.50187 | -0.22383 |
| O | -3.19598 | -0.05001 | -2.20653 |
| C | -3.28153 | 0.39630 | -1.08529 |
| C | -4.54494 | 0.90899 | -0.43702 |
| C | -1.32456 | -1.27178 | 1.54555 |
| O | -1.34207 | -1.02147 | 2.72913 |
| O | -2.20705 | -2.12660 | 0.96953 |
| H | -1.12709 | -0.65558 | -1.52509 |
| H | 0.65099 | 0.96266 | -1.80298 |
| H | -0.60349 | 2.07087 | -1.24808 |
| H | 1.02429 | 0.23084 | 2.13461 |
| H | -0.13391 | -2.74377 | -0.28347 |
| H | 1.04123 | -2.40264 | 0.97963 |
| H | 1.19566 | -1.66305 | -2.00019 |
| H | 3.22441 | -1.22193 | -2.96001 |
| H | 4.31019 | -0.26949 | -1.92652 |
| H | 4.57560 | -1.99907 | -2.11013 |
| H | 3.00018 | -1.79941 | 1.33589 |
| H | 4.49320 | -2.18240 | 0.46220 |
| H | 4.02903 | -0.50280 | 0.72062 |
| H | 2.21237 | 4.14855 | -0.04232 |
| H | -5.35745 | 0.89783 | -1.16414 |
| H | -4.80413 | 0.27449 | 0.41732 |
| H | -4.39303 | 1.92321 | -0.05506 |
| H | -2.79391 | -2.43082 | 1.68748 |

**3h**

| C | 0.28513 | -0.05063 | -0.02834 |
| --- | --- | --- | --- |
| C | -1.00562 | -0.57592 | -0.73098 |
| C | -1.82543 | 0.68995 | -1.06120 |
| C | -1.29434 | 1.70446 | -0.07049 |
| C | -0.16318 | 1.28696 | 0.51475 |
| C | 1.42774 | 0.12307 | -1.09352 |
| C | 2.67392 | 0.77239 | -0.55451 |
| C | 3.89241 | 0.22739 | -0.40793 |
| C | 5.03739 | 1.04916 | 0.13518 |
| C | 4.25770 | -1.19424 | -0.76047 |
| C | -1.91304 | 3.01365 | 0.22095 |
| O | -1.49237 | 3.84093 | 1.00430 |
| O | -3.04616 | 3.20859 | -0.50659 |
| O | -1.72185 | -1.37684 | 0.23795 |
| O | -2.72921 | -2.52552 | -1.43639 |
| C | -2.54586 | -2.33851 | -0.25463 |
| C | -3.17975 | -3.11872 | 0.87162 |
| C | 0.77228 | -0.98206 | 1.07619 |
| O | 0.89619 | -0.68959 | 2.24226 |
| O | 1.09602 | -2.20910 | 0.59250 |
| H | -0.79814 | -1.19688 | -1.60260 |
| H | -1.65764 | 1.01212 | -2.09741 |
| H | -2.89859 | 0.50356 | -0.96305 |
| H | 0.39189 | 1.84318 | 1.25988 |
| H | 1.01744 | 0.74524 | -1.90104 |
| H | 1.63742 | -0.85926 | -1.52346 |
| H | 2.55410 | 1.81495 | -0.25884 |
| H | 4.73433 | 2.07576 | 0.36223 |
| H | 5.44150 | 0.60171 | 1.05375 |
| H | 5.86840 | 1.08933 | -0.58294 |
| H | 3.40350 | -1.80351 | -1.06162 |
| H | 4.99646 | -1.21236 | -1.57386 |
| H | 4.73301 | -1.68814 | 0.09794 |
| H | -3.37948 | 4.08820 | -0.24800 |
| H | -3.86054 | -3.86396 | 0.45921 |
| H | -2.40404 | -3.61133 | 1.46714 |
| H | -3.72271 | -2.44308 | 1.54027 |
| H | 1.40611 | -2.72511 | 1.36042 |

**3i**

| C | 0.03107 | -0.25733 | 0.89622 |
| --- | --- | --- | --- |
| C | 0.04811 | 0.44180 | -0.49335 |
| C | 1.15962 | -0.28148 | -1.28102 |
| C | 2.05193 | -0.81996 | -0.18406 |
| C | 1.44900 | -0.77928 | 1.01266 |
| C | -0.95073 | -1.49875 | 0.90443 |
| C | -2.39576 | -1.20270 | 0.60701 |
| C | -3.12266 | -1.63300 | -0.43794 |
| C | -4.57903 | -1.25166 | -0.56261 |
| C | -2.61562 | -2.50936 | -1.55743 |
| C | 3.40006 | -1.33642 | -0.47713 |
| O | 3.88798 | -1.37773 | -1.59038 |
| O | 4.06270 | -1.77177 | 0.62583 |
| O | 0.45500 | 1.81427 | -0.27610 |
| O | -0.81587 | 2.50154 | -2.02603 |
| C | -0.07454 | 2.75263 | -1.10303 |
| C | 0.39141 | 4.13101 | -0.69888 |
| C | -0.31914 | 0.65633 | 2.06569 |
| O | 0.26034 | 0.66122 | 3.12943 |
| O | -1.40342 | 1.43386 | 1.83090 |
| H | -0.92070 | 0.44013 | -0.98976 |
| H | 0.75091 | -1.09355 | -1.89726 |
| H | 1.67713 | 0.39852 | -1.96341 |
| H | 1.87156 | -1.10991 | 1.95342 |
| H | -0.86200 | -1.95898 | 1.89842 |
| H | -0.54351 | -2.22610 | 0.19700 |
| H | -2.89766 | -0.57036 | 1.33640 |
| H | -4.90836 | -0.61723 | 0.26580 |
| H | -4.76411 | -0.71058 | -1.50074 |
| H | -5.21991 | -2.14416 | -0.58587 |
| H | -1.56193 | -2.78188 | -1.46223 |
| H | -3.19685 | -3.44016 | -1.61051 |
| H | -2.74584 | -2.00764 | -2.52589 |
| H | 4.93138 | -2.07941 | 0.30711 |
| H | 0.01414 | 4.86593 | -1.41063 |
| H | 0.02359 | 4.36482 | 0.30591 |
| H | 1.48471 | 4.16786 | -0.66305 |
| H | -1.54878 | 1.94070 | 2.65280 |

**3j**

| C | 0.26881 | -0.07841 | -0.07309 |
| --- | --- | --- | --- |
| C | -1.05154 | -0.55929 | -0.76068 |
| C | -1.82960 | 0.73060 | -1.09062 |
| C | -1.24162 | 1.73889 | -0.13041 |
| C | -0.11470 | 1.29109 | 0.44243 |
| C | 1.41325 | 0.00883 | -1.14112 |
| C | 2.67631 | 0.65591 | -0.63800 |
| C | 3.86908 | 0.07779 | -0.42046 |
| C | 5.04087 | 0.90213 | 0.05862 |
| C | 4.17822 | -1.38435 | -0.63295 |
| C | -1.87410 | 3.05284 | 0.07939 |
| O | -2.88767 | 3.42045 | -0.48285 |
| O | -1.21235 | 3.83446 | 0.97285 |
| O | -1.78549 | -1.33455 | 0.21512 |
| O | -2.79454 | -2.49796 | -1.44888 |
| C | -2.60953 | -2.30248 | -0.26935 |
| C | -3.23110 | -3.08214 | 0.86364 |
| C | 0.71134 | -1.08165 | 0.99123 |
| O | 1.09673 | -2.20432 | 0.74899 |
| O | 0.64407 | -0.58918 | 2.24729 |
| H | -0.86876 | -1.19023 | -1.63037 |
| H | -1.67424 | 1.03550 | -2.13368 |
| H | -2.90714 | 0.59274 | -0.96416 |
| H | 0.48563 | 1.84471 | 1.15329 |
| H | 1.02276 | 0.59067 | -1.98728 |
| H | 1.59559 | -1.00586 | -1.50300 |
| H | 2.59685 | 1.72648 | -0.44544 |
| H | 4.77861 | 1.95740 | 0.18318 |
| H | 5.41974 | 0.52750 | 1.01968 |
| H | 5.87924 | 0.84090 | -0.64934 |
| H | 3.29735 | -1.98961 | -0.85190 |
| H | 4.90143 | -1.50967 | -1.45111 |
| H | 4.65036 | -1.80615 | 0.26431 |
| H | -1.72418 | 4.66271 | 1.02678 |
| H | -3.97036 | -3.77684 | 0.46378 |
| H | -2.45178 | -3.63929 | 1.39503 |
| H | -3.69939 | -2.40368 | 1.58329 |
| H | 0.94819 | -1.30713 | 2.83513 |

**3k**

| C | 0.06800 | -0.61904 | -0.00067 |
| --- | --- | --- | --- |
| C | -1.22196 | -0.16817 | -0.75477 |
| C | -1.06959 | 1.35762 | -0.93523 |
| C | -0.08289 | 1.73203 | 0.15062 |
| C | 0.51331 | 0.65441 | 0.67935 |
| C | 1.14598 | -1.09972 | -1.03841 |
| C | 2.45574 | -1.50086 | -0.41479 |
| C | 3.63846 | -0.86952 | -0.48757 |
| C | 4.85373 | -1.43667 | 0.20720 |
| C | 3.89201 | 0.41709 | -1.23419 |
| C | 0.22901 | 3.11166 | 0.57553 |
| O | 1.03168 | 3.42982 | 1.42977 |
| O | -0.50107 | 4.02898 | -0.11549 |
| O | -2.34391 | -0.44505 | 0.11628 |
| O | -3.73046 | -0.51518 | -1.67494 |
| C | -3.55178 | -0.61645 | -0.48203 |
| C | -4.60848 | -0.94594 | 0.54451 |
| C | -0.20765 | -1.73611 | 1.00057 |
| O | -0.03475 | -1.67096 | 2.19536 |
| O | -0.64520 | -2.86472 | 0.38622 |
| H | -1.37794 | -0.70102 | -1.69300 |
| H | -0.68254 | 1.60234 | -1.93330 |
| H | -2.03413 | 1.86482 | -0.84372 |
| H | 1.25926 | 0.67681 | 1.46358 |
| H | 1.28026 | -0.29705 | -1.76965 |
| H | 0.71940 | -1.95638 | -1.57475 |
| H | 2.41690 | -2.42361 | 0.16567 |
| H | 4.62602 | -2.36583 | 0.73846 |
| H | 5.65857 | -1.64253 | -0.51227 |
| H | 5.26161 | -0.71987 | 0.93316 |
| H | 3.00683 | 0.81334 | -1.73645 |
| H | 4.25801 | 1.19205 | -0.54727 |
| H | 4.67626 | 0.27603 | -1.99060 |
| H | -0.23058 | 4.89937 | 0.23220 |
| H | -5.57556 | -1.04634 | 0.05096 |
| H | -4.35294 | -1.87937 | 1.05685 |
| H | -4.65443 | -0.15989 | 1.30500 |
| H | -0.78868 | -3.51809 | 1.09668 |

**3l**

| C | -0.31002 | -0.56689 | 0.62683 |
| --- | --- | --- | --- |
| C | -0.93554 | -0.05176 | -0.71764 |
| C | 0.00180 | 1.07150 | -1.20589 |
| C | 0.78254 | 1.44444 | 0.03153 |
| C | 0.59791 | 0.56942 | 1.02960 |
| C | 0.49501 | -1.90642 | 0.42091 |
| C | 1.51197 | -1.87806 | -0.68816 |
| C | 2.84526 | -1.75440 | -0.58384 |
| C | 3.71761 | -1.76590 | -1.81658 |
| C | 3.59974 | -1.59740 | 0.71342 |
| C | 1.65473 | 2.63120 | 0.05379 |
| O | 1.80143 | 3.39019 | -0.88487 |
| O | 2.29894 | 2.80716 | 1.23838 |
| O | -2.23100 | 0.51780 | -0.42345 |
| O | -3.20504 | -0.69654 | -2.08122 |
| C | -3.29486 | 0.06374 | -1.14574 |
| C | -4.57915 | 0.63264 | -0.59580 |
| C | -1.41183 | -0.89257 | 1.63618 |
| O | -2.25955 | -1.74223 | 1.47378 |
| O | -1.32746 | -0.15857 | 2.76997 |
| H | -1.08041 | -0.84770 | -1.44655 |
| H | 0.67195 | 0.71598 | -1.99787 |
| H | -0.56903 | 1.90874 | -1.61891 |
| H | 1.06919 | 0.62704 | 2.00337 |
| H | -0.25162 | -2.68229 | 0.21539 |
| H | 0.96196 | -2.16348 | 1.37796 |
| H | 1.09989 | -1.98384 | -1.69219 |
| H | 3.13044 | -1.87331 | -2.73380 |
| H | 4.30398 | -0.83974 | -1.89320 |
| H | 4.44308 | -2.59040 | -1.77784 |
| H | 2.96299 | -1.66041 | 1.59881 |
| H | 4.37915 | -2.36625 | 0.80236 |
| H | 4.11382 | -0.62697 | 0.74306 |
| H | 2.83035 | 3.61745 | 1.12929 |
| H | -5.38813 | 0.46091 | -1.30677 |
| H | -4.81303 | 0.12566 | 0.34733 |
| H | -4.47450 | 1.70035 | -0.38390 |
| H | -2.05956 | -0.46155 | 3.34096 |

**3m**

| C | 0.04379 | -0.61982 | -0.00622 |
| --- | --- | --- | --- |
| C | -1.23554 | -0.15047 | -0.77701 |
| C | -1.06258 | 1.37011 | -0.96867 |
| C | -0.05683 | 1.73991 | 0.09678 |
| C | 0.52802 | 0.66034 | 0.63596 |
| C | 1.09634 | -1.17471 | -1.02512 |
| C | 2.40496 | -1.57066 | -0.39515 |
| C | 3.60162 | -0.97403 | -0.51748 |
| C | 4.81472 | -1.53739 | 0.18462 |
| C | 3.87482 | 0.26429 | -1.33576 |
| C | 0.22133 | 3.14864 | 0.42575 |
| O | -0.31881 | 4.09541 | -0.11348 |
| O | 1.15364 | 3.30222 | 1.40275 |
| O | -2.37407 | -0.41441 | 0.07571 |
| O | -3.72680 | -0.47541 | -1.74181 |
| C | -3.57168 | -0.58402 | -0.54679 |
| C | -4.64504 | -0.92998 | 0.45631 |
| C | -0.30902 | -1.74447 | 0.96860 |
| O | -0.56355 | -2.88073 | 0.63582 |
| O | -0.29622 | -1.34747 | 2.26032 |
| H | -1.38051 | -0.68954 | -1.71337 |
| H | -0.68530 | 1.60604 | -1.97223 |
| H | -2.01472 | 1.89795 | -0.86462 |
| H | 1.29632 | 0.68065 | 1.39830 |
| H | 1.23671 | -0.42166 | -1.80618 |
| H | 0.64111 | -2.05527 | -1.49467 |
| H | 2.35057 | -2.46434 | 0.22764 |
| H | 4.57278 | -2.43225 | 0.76623 |
| H | 5.59967 | -1.80258 | -0.53741 |
| H | 5.25710 | -0.79630 | 0.86494 |
| H | 2.98787 | 0.66538 | -1.83096 |
| H | 4.28926 | 1.05957 | -0.70125 |
| H | 4.62954 | 0.05856 | -2.10730 |
| H | 1.24356 | 4.26481 | 1.53007 |
| H | -5.61264 | -0.96496 | -0.04517 |
| H | -4.42745 | -1.90483 | 0.90611 |
| H | -4.66436 | -0.19205 | 1.26424 |
| H | -0.54241 | -2.13249 | 2.78632 |

## 3.5 ECD calculation of 5

Conformation search of **5** at MMFF94s force field gave twelve conformers **5a**-**5l**. These conformers were optimized at B3LYP/6-31G(d) level, and then calculated the ECD at B3LYP/6-31G(d,p) level.

| Conformers | Gibbs free energies | Population (%) |
| --- | --- | --- |
| **5a** | -921.362785 | 1.29 |
| **5b** | -921.363564 | 2.93 |
| **5c** | -921.363509 | 2.77 |
| **5d** | -921.365134 | 15.48 |
| **5e** | -921.362900 | 1.45 |
| **5f** | -921.364615 | 8.93 |
| **5g** | -921.363087 | 1.77 |
| **5h** | -921.364637 | 9.14 |
| **5i** | -921.363537 | 2.85 |
| **5j** | -921.363570 | 2.95 |
| **5k** | -921.362660 | 1.13 |
| **5l** | -921.366228 | 49.31 |

Standard orientation of optimized **5** at B3LYP/6-31G(d) level.

**5a**

| C | 4.73735 | 2.49221 | -0.15150 |
| --- | --- | --- | --- |
| C | 4.07889 | 1.18226 | -0.51584 |
| C | 5.03154 | 0.01705 | -0.63009 |
| C | 2.75351 | 1.11977 | -0.72317 |
| C | 1.92438 | -0.07655 | -1.11144 |
| C | 0.88799 | -0.49653 | -0.02856 |
| C | 0.03330 | -1.75664 | -0.46382 |
| C | -1.31057 | -1.55627 | 0.26999 |
| C | -1.40462 | -0.04733 | 0.39433 |
| C | -0.20396 | 0.52255 | 0.23797 |
| C | -2.67419 | 0.65750 | 0.73904 |
| O | -3.65723 | 0.32402 | -0.27642 |
| C | -4.92049 | 0.74891 | -0.02903 |
| C | -5.85176 | 0.34262 | -1.14770 |
| O | -5.23473 | 1.37171 | 0.96054 |
| O | -0.15497 | -1.81882 | -1.86904 |
| C | 1.62156 | -0.81236 | 1.27781 |
| O | 1.40771 | -0.31350 | 2.35973 |
| O | 2.57167 | -1.77074 | 1.10421 |
| H | 5.25172 | 2.41946 | 0.81695 |
| H | 5.50377 | 2.76747 | -0.88979 |
| H | 4.01482 | 3.31205 | -0.09082 |
| H | 5.61954 | -0.08713 | 0.29221 |
| H | 4.53259 | -0.93569 | -0.81471 |
| H | 5.75562 | 0.18887 | -1.43892 |
| H | 2.19299 | 2.04895 | -0.60993 |
| H | 1.36215 | 0.14980 | -2.02499 |
| H | 2.54766 | -0.94202 | -1.34608 |
| H | 0.53818 | -2.68890 | -0.20448 |
| H | -1.30183 | -2.02159 | 1.26704 |
| H | -2.13449 | -2.00880 | -0.29197 |
| H | -0.00494 | 1.58538 | 0.33315 |
| H | -3.06400 | 0.34053 | 1.71338 |
| H | -2.54349 | 1.74343 | 0.77139 |
| H | -5.52055 | 0.78632 | -2.09251 |
| H | -5.83887 | -0.74469 | -1.27528 |
| H | -6.86324 | 0.67806 | -0.91669 |
| H | -0.70788 | -1.05880 | -2.11799 |
| H | 2.98309 | -1.89721 | 1.97994 |

**5b**

| C | 5.43219 | -1.40370 | 0.31025 |
| --- | --- | --- | --- |
| C | 4.34207 | -0.68363 | -0.44940 |
| C | 4.83157 | 0.44878 | -1.31896 |
| C | 3.06295 | -1.07504 | -0.33080 |
| C | 1.84878 | -0.50728 | -1.02037 |
| C | 0.85911 | 0.20530 | -0.05971 |
| C | 0.29001 | -0.70625 | 1.09067 |
| C | -1.13859 | -0.16952 | 1.34302 |
| C | -1.51187 | 0.44962 | 0.01065 |
| C | -0.42746 | 0.64945 | -0.74955 |
| C | -2.91140 | 0.83441 | -0.34100 |
| O | -3.73578 | -0.35588 | -0.25232 |
| C | -5.07237 | -0.14930 | -0.35339 |
| C | -5.82620 | -1.45410 | -0.24466 |
| O | -5.56881 | 0.94341 | -0.51234 |
| O | 0.25210 | -2.07608 | 0.70559 |
| C | 1.52671 | 1.48661 | 0.45167 |
| O | 2.17341 | 2.24843 | -0.23389 |
| O | 1.27735 | 1.74282 | 1.76175 |
| H | 6.18369 | -1.81830 | -0.37670 |
| H | 5.97038 | -0.71531 | 0.97718 |
| H | 5.03631 | -2.22522 | 0.91549 |
| H | 5.48462 | 0.06778 | -2.11719 |
| H | 4.02562 | 1.02658 | -1.77323 |
| H | 5.44000 | 1.14672 | -0.72803 |
| H | 2.84583 | -1.91397 | 0.32887 |
| H | 2.12344 | 0.20637 | -1.80087 |
| H | 1.30300 | -1.32324 | -1.50955 |
| H | 0.91766 | -0.67907 | 1.98101 |
| H | -1.15041 | 0.58817 | 2.13789 |
| H | -1.80859 | -0.97948 | 1.65124 |
| H | -0.41923 | 1.11225 | -1.73275 |
| H | -3.31243 | 1.58364 | 0.35177 |
| H | -2.98104 | 1.24661 | -1.35246 |
| H | -5.52282 | -2.13159 | -1.04972 |
| H | -5.59242 | -1.94856 | 0.70376 |
| H | -6.89723 | -1.26000 | -0.30907 |
| H | -0.37800 | -2.15327 | -0.03031 |
| H | 1.71253 | 2.59617 | 1.95183 |

**5c**

| C | -4.31105 | 2.61056 | -0.92734 |
| --- | --- | --- | --- |
| C | -3.31295 | 1.99810 | 0.02731 |
| C | -2.55898 | 3.00156 | 0.86619 |
| C | -3.15833 | 0.66564 | 0.09274 |
| C | -2.23611 | -0.12583 | 0.98263 |
| C | -1.12250 | -0.87966 | 0.20405 |
| C | -0.17539 | -1.72882 | 1.15316 |
| C | 1.17450 | -1.73377 | 0.40282 |
| C | 1.13661 | -0.42511 | -0.36428 |
| C | -0.11735 | 0.02841 | -0.47513 |
| C | 2.35186 | 0.19023 | -0.97360 |
| O | 3.31037 | 0.42974 | 0.09123 |
| C | 4.53730 | 0.84382 | -0.30888 |
| C | 5.44702 | 1.05282 | 0.87973 |
| O | 4.83926 | 1.01828 | -1.46834 |
| O | -0.04599 | -1.14745 | 2.44026 |
| C | -1.72709 | -1.84092 | -0.82447 |
| O | -1.34375 | -2.00726 | -1.96113 |
| O | -2.75592 | -2.56264 | -0.30354 |
| H | -5.06439 | 3.20044 | -0.38628 |
| H | -3.81882 | 3.30248 | -1.62518 |
| H | -4.83408 | 1.85038 | -1.51578 |
| H | -3.25449 | 3.59750 | 1.47337 |
| H | -1.83306 | 2.54462 | 1.54235 |
| H | -2.02050 | 3.71412 | 0.22603 |
| H | -3.78045 | 0.06114 | -0.56893 |
| H | -1.74995 | 0.50852 | 1.72575 |
| H | -2.81688 | -0.86684 | 1.54449 |
| H | -0.58013 | -2.72725 | 1.33312 |
| H | 1.24558 | -2.58135 | -0.29423 |
| H | 2.01187 | -1.81545 | 1.10399 |
| H | -0.42004 | 0.92116 | -1.01361 |
| H | 2.81668 | -0.47248 | -1.71284 |
| H | 2.12420 | 1.13675 | -1.47311 |
| H | 5.02265 | 1.80674 | 1.55100 |
| H | 5.54246 | 0.12359 | 1.45093 |
| H | 6.42825 | 1.37739 | 0.53213 |
| H | 0.43120 | -0.30790 | 2.32678 |
| H | -3.04715 | -3.16114 | -1.01733 |

**5d**

| C | 5.45887 | -1.34645 | 0.22929 |
| --- | --- | --- | --- |
| C | 4.34937 | -0.61855 | -0.49330 |
| C | 4.81271 | 0.55186 | -1.32684 |
| C | 3.07728 | -1.03221 | -0.37699 |
| C | 1.84647 | -0.45523 | -1.02665 |
| C | 0.84490 | 0.17104 | -0.01751 |
| C | 0.26729 | -0.83532 | 1.04118 |
| C | -1.14966 | -0.29605 | 1.34378 |
| C | -1.51801 | 0.43584 | 0.06707 |
| C | -0.43101 | 0.68556 | -0.67508 |
| C | -2.91260 | 0.86614 | -0.24904 |
| O | -3.75242 | -0.31681 | -0.25503 |
| C | -5.08463 | -0.08581 | -0.35830 |
| C | -5.85661 | -1.38451 | -0.34599 |
| O | -5.56497 | 1.02184 | -0.44910 |
| O | 0.21185 | -2.16486 | 0.53770 |
| C | 1.49015 | 1.35116 | 0.71198 |
| O | 1.53324 | 1.51211 | 1.91243 |
| O | 1.99129 | 2.27112 | -0.15299 |
| H | 6.20919 | -1.72577 | -0.47899 |
| H | 5.99246 | -0.67317 | 0.91491 |
| H | 5.08145 | -2.19360 | 0.81009 |
| H | 5.48313 | 0.21335 | -2.12947 |
| H | 3.99432 | 1.11465 | -1.77943 |
| H | 5.39741 | 1.25087 | -0.71284 |
| H | 2.87992 | -1.89782 | 0.25393 |
| H | 2.09815 | 0.30156 | -1.77400 |
| H | 1.31396 | -1.25701 | -1.55268 |
| H | 0.89913 | -0.88302 | 1.92735 |
| H | -1.13882 | 0.39648 | 2.19638 |
| H | -1.83486 | -1.11355 | 1.59286 |
| H | -0.41931 | 1.22675 | -1.61719 |
| H | -3.30287 | 1.56169 | 0.50336 |
| H | -2.97839 | 1.35997 | -1.22351 |
| H | -5.54089 | -2.01835 | -1.18119 |
| H | -5.65315 | -1.93609 | 0.57769 |
| H | -6.92329 | -1.17252 | -0.42465 |
| H | -0.43280 | -2.17318 | -0.18968 |
| H | 2.35632 | 2.98674 | 0.40132 |

**5e**

| C | -5.62766 | 0.51601 | 0.66778 |
| --- | --- | --- | --- |
| C | -4.47587 | 0.09039 | -0.21316 |
| C | -4.75647 | -1.12514 | -1.06289 |
| C | -3.32701 | 0.78580 | -0.20764 |
| C | -2.08647 | 0.53781 | -1.02725 |
| C | -0.86099 | 0.08453 | -0.18853 |
| C | -0.41650 | 1.10034 | 0.92825 |
| C | 1.11907 | 0.93119 | 1.02101 |
| C | 1.49391 | 0.42301 | -0.35641 |
| C | 0.41945 | -0.03754 | -1.00993 |
| C | 2.89254 | 0.45779 | -0.88062 |
| O | 3.73516 | -0.25922 | 0.05549 |
| C | 5.06765 | -0.17976 | -0.18232 |
| C | 5.84390 | -0.96166 | 0.85126 |
| O | 5.54488 | 0.43829 | -1.10777 |
| O | -0.75814 | 2.43982 | 0.58701 |
| C | -1.14443 | -1.32399 | 0.34676 |
| O | -1.63908 | -2.22006 | -0.30226 |
| O | -0.72990 | -1.51306 | 1.62519 |
| H | -6.52320 | 0.73315 | 0.06829 |
| H | -5.90988 | -0.28590 | 1.36462 |
| H | -5.38759 | 1.40855 | 1.25400 |
| H | -5.55479 | -0.91298 | -1.78848 |
| H | -3.88113 | -1.48833 | -1.60295 |
| H | -5.11911 | -1.95149 | -0.43669 |
| H | -3.25725 | 1.64972 | 0.45174 |
| H | -2.25561 | -0.21757 | -1.79837 |
| H | -1.80955 | 1.46490 | -1.54423 |
| H | -0.92186 | 0.91393 | 1.87536 |
| H | 1.40159 | 0.20006 | 1.78803 |
| H | 1.59700 | 1.88224 | 1.28730 |
| H | 0.42516 | -0.49581 | -1.99515 |
| H | 2.97205 | -0.00630 | -1.86868 |
| H | 3.26947 | 1.48438 | -0.96346 |
| H | 5.61434 | -0.59262 | 1.85608 |
| H | 5.55536 | -2.01747 | 0.81728 |
| H | 6.91165 | -0.86358 | 0.65312 |
| H | -0.27686 | 2.66529 | -0.22643 |
| H | -0.91979 | -2.44891 | 1.82950 |

**5f**

| C | -5.62500 | 0.48529 | 0.65703 |
| --- | --- | --- | --- |
| C | -4.47062 | 0.04804 | -0.21431 |
| C | -4.74332 | -1.19151 | -1.03194 |
| C | -3.32656 | 0.75072 | -0.22879 |
| C | -2.08149 | 0.48703 | -1.03502 |
| C | -0.84659 | 0.11009 | -0.17024 |
| C | -0.39489 | 1.21847 | 0.84656 |
| C | 1.13718 | 1.03377 | 0.95519 |
| C | 1.50583 | 0.40701 | -0.37529 |
| C | 0.42509 | -0.09623 | -0.98620 |
| C | 2.90435 | 0.37999 | -0.90023 |
| O | 3.73824 | -0.26570 | 0.09336 |
| C | 5.07080 | -0.23125 | -0.15350 |
| C | 5.83733 | -0.92752 | 0.94634 |
| O | 5.55488 | 0.29155 | -1.13271 |
| O | -0.72422 | 2.52622 | 0.39188 |
| C | -1.11031 | -1.19122 | 0.59159 |
| O | -0.98013 | -1.36068 | 1.78426 |
| O | -1.47701 | -2.20041 | -0.24057 |
| H | -6.52270 | 0.68221 | 0.05382 |
| H | -5.89960 | -0.30166 | 1.37354 |
| H | -5.38994 | 1.39240 | 1.22211 |
| H | -5.56888 | -1.01579 | -1.73607 |
| H | -3.87782 | -1.53800 | -1.59961 |
| H | -5.06553 | -2.01585 | -0.38066 |
| H | -3.26334 | 1.63263 | 0.40719 |
| H | -2.23559 | -0.30390 | -1.77343 |
| H | -1.82072 | 1.39475 | -1.59325 |
| H | -0.90181 | 1.10746 | 1.80430 |
| H | 1.40317 | 0.36191 | 1.78051 |
| H | 1.63279 | 1.99390 | 1.14578 |
| H | 0.42572 | -0.63432 | -1.93002 |
| H | 2.97748 | -0.16658 | -1.84575 |
| H | 3.29399 | 1.39123 | -1.06930 |
| H | 5.62871 | -0.45299 | 1.91070 |
| H | 5.51987 | -1.97272 | 1.02202 |
| H | 6.90511 | -0.87887 | 0.73080 |
| H | -0.22698 | 2.68407 | -0.42800 |
| H | -1.59884 | -2.98385 | 0.32860 |

**5g**

| C | -5.56393 | 0.05275 | -0.64092 |
| --- | --- | --- | --- |
| C | -4.21003 | -0.61864 | -0.63047 |
| C | -4.24141 | -2.08013 | -0.25482 |
| C | -3.10727 | 0.07688 | -0.95234 |
| C | -1.68469 | -0.41621 | -1.02510 |
| C | -0.75212 | 0.19359 | 0.05565 |
| C | -0.64789 | 1.76211 | 0.02260 |
| C | 0.79346 | 2.06663 | 0.49848 |
| C | 1.54894 | 0.80587 | 0.12640 |
| C | 0.71096 | -0.20921 | -0.11695 |
| C | 3.04214 | 0.78855 | 0.10008 |
| O | 3.50210 | -0.52423 | -0.26385 |
| C | 4.84866 | -0.68247 | -0.30607 |
| C | 5.20970 | -2.09667 | -0.69341 |
| O | 5.63139 | 0.20798 | -0.06122 |
| O | -0.86560 | 2.27759 | -1.28621 |
| C | -1.17871 | -0.35736 | 1.42063 |
| O | -1.49429 | -1.50780 | 1.63399 |
| O | -1.11263 | 0.55990 | 2.42083 |
| H | -6.24871 | -0.45108 | -1.33794 |
| H | -6.03862 | 0.00112 | 0.34921 |
| H | -5.49735 | 1.10554 | -0.93273 |
| H | -4.80066 | -2.65924 | -1.00365 |
| H | -3.24969 | -2.51995 | -0.14212 |
| H | -4.76874 | -2.21655 | 0.69904 |
| H | -3.22565 | 1.12840 | -1.21023 |
| H | -1.62822 | -1.50344 | -0.93301 |
| H | -1.26741 | -0.15818 | -2.00635 |
| H | -1.40608 | 2.23862 | 0.64348 |
| H | 0.83122 | 2.23715 | 1.58242 |
| H | 1.17315 | 2.97422 | 0.01234 |
| H | 1.00532 | -1.22398 | -0.36045 |
| H | 3.43720 | 1.52322 | -0.61418 |
| H | 3.45750 | 1.06143 | 1.07930 |
| H | 4.78727 | -2.80432 | 0.02748 |
| H | 4.78748 | -2.33866 | -1.67429 |
| H | 6.29490 | -2.19867 | -0.72133 |
| H | -0.17935 | 1.90256 | -1.86309 |
| H | -1.35813 | 0.08020 | 3.23520 |

**5h**

| C | -5.56889 | 0.04405 | -0.55678 |
| --- | --- | --- | --- |
| C | -4.20758 | -0.60800 | -0.62633 |
| C | -4.21706 | -2.09601 | -0.37173 |
| C | -3.11773 | 0.12372 | -0.90860 |
| C | -1.69015 | -0.34028 | -1.03715 |
| C | -0.74633 | 0.23680 | 0.05327 |
| C | -0.62918 | 1.80133 | 0.05587 |
| C | 0.80626 | 2.07291 | 0.56563 |
| C | 1.55609 | 0.81759 | 0.16054 |
| C | 0.71099 | -0.18137 | -0.12341 |
| C | 3.04908 | 0.78745 | 0.15053 |
| O | 3.50228 | -0.51761 | -0.24976 |
| C | 4.84778 | -0.68048 | -0.30061 |
| C | 5.20139 | -2.08532 | -0.72763 |
| O | 5.63582 | 0.19904 | -0.03446 |
| O | -0.82564 | 2.35416 | -1.24026 |
| C | -1.18370 | -0.23275 | 1.44216 |
| O | -1.36106 | 0.47339 | 2.41163 |
| O | -1.31054 | -1.58340 | 1.50810 |
| H | -6.26056 | -0.40689 | -1.28260 |
| H | -6.02441 | -0.09629 | 0.43366 |
| H | -5.51830 | 1.11843 | -0.75832 |
| H | -4.81447 | -2.61438 | -1.13507 |
| H | -3.22070 | -2.54129 | -0.35681 |
| H | -4.69427 | -2.31603 | 0.59337 |
| H | -3.25296 | 1.19103 | -1.07895 |
| H | -1.61305 | -1.43017 | -1.01342 |
| H | -1.29741 | -0.01726 | -2.00932 |
| H | -1.39073 | 2.25832 | 0.68666 |
| H | 0.82257 | 2.20071 | 1.65619 |
| H | 1.20544 | 2.99434 | 0.12309 |
| H | 0.99971 | -1.18977 | -0.39742 |
| H | 3.45892 | 1.54060 | -0.53546 |
| H | 3.45526 | 1.02523 | 1.14267 |
| H | 4.77859 | -2.81058 | -0.02467 |
| H | 4.77495 | -2.29874 | -1.71331 |
| H | 6.28604 | -2.19108 | -0.76207 |
| H | -0.12450 | 2.00305 | -1.81449 |
| H | -1.56284 | -1.78164 | 2.42983 |

**5i**

| C | 4.75268 | 2.41960 | -0.42013 |
| --- | --- | --- | --- |
| C | 4.07654 | 1.08309 | -0.61745 |
| C | 4.99885 | -0.10660 | -0.50224 |
| C | 2.76197 | 1.01730 | -0.88532 |
| C | 1.92781 | -0.21054 | -1.14137 |
| C | 0.89356 | -0.52188 | -0.01809 |
| C | 0.03337 | -1.80843 | -0.33689 |
| C | -1.30346 | -1.54178 | 0.38945 |
| C | -1.39113 | -0.02655 | 0.38814 |
| C | -0.18904 | 0.52291 | 0.17095 |
| C | -2.65475 | 0.71121 | 0.68180 |
| O | -3.64730 | 0.30134 | -0.29543 |
| C | -4.90780 | 0.74645 | -0.06997 |
| C | -5.85022 | 0.25577 | -1.14439 |
| O | -5.21149 | 1.44503 | 0.87124 |
| O | -0.17273 | -1.99065 | -1.72918 |
| C | 1.69941 | -0.78484 | 1.25840 |
| O | 2.45783 | -1.72062 | 1.40308 |
| O | 1.50465 | 0.13958 | 2.22728 |
| H | 5.21657 | 2.48513 | 0.57406 |
| H | 5.56295 | 2.56227 | -1.14903 |
| H | 4.05229 | 3.25429 | -0.52454 |
| H | 5.55416 | -0.06750 | 0.44452 |
| H | 4.47919 | -1.06550 | -0.53601 |
| H | 5.75137 | -0.09085 | -1.30332 |
| H | 2.21957 | 1.96253 | -0.93985 |
| H | 1.36680 | -0.08572 | -2.07433 |
| H | 2.54861 | -1.09983 | -1.27401 |
| H | 0.54736 | -2.71022 | -0.00110 |
| H | -1.28962 | -1.92563 | 1.42103 |
| H | -2.13449 | -2.03465 | -0.12612 |
| H | 0.01300 | 1.58952 | 0.16571 |
| H | -3.04055 | 0.47832 | 1.68132 |
| H | -2.51831 | 1.79547 | 0.62333 |
| H | -5.52748 | 0.62343 | -2.12412 |
| H | -5.83980 | -0.83821 | -1.18654 |
| H | -6.85903 | 0.61023 | -0.93081 |
| H | -0.72974 | -1.25530 | -2.03631 |
| H | 2.09270 | -0.11951 | 2.96281 |

**5j**

| C | -5.63823 | 0.50994 | 0.58074 |
| --- | --- | --- | --- |
| C | -4.46810 | 0.04181 | -0.25290 |
| C | -4.72974 | -1.22203 | -1.03625 |
| C | -3.32128 | 0.74011 | -0.26814 |
| C | -2.06178 | 0.45184 | -1.04254 |
| C | -0.84036 | 0.09704 | -0.14622 |
| C | -0.38254 | 1.23768 | 0.81698 |
| C | 1.15795 | 1.09452 | 0.89367 |
| C | 1.51132 | 0.36914 | -0.38698 |
| C | 0.42694 | -0.17503 | -0.94796 |
| C | 2.90216 | 0.31271 | -0.92635 |
| O | 3.75854 | -0.24355 | 0.10424 |
| C | 5.08484 | -0.23276 | -0.17060 |
| C | 5.87310 | -0.83520 | 0.96932 |
| O | 5.55171 | 0.20245 | -1.19997 |
| O | -0.74887 | 2.48052 | 0.21488 |
| C | -1.13203 | -1.16290 | 0.67444 |
| O | -1.02469 | -1.28038 | 1.87594 |
| O | -1.49332 | -2.20540 | -0.11726 |
| H | -6.52288 | 0.69195 | -0.04610 |
| H | -5.93127 | -0.25310 | 1.31575 |
| H | -5.41089 | 1.43344 | 1.12228 |
| H | -5.54155 | -1.06636 | -1.76089 |
| H | -3.85481 | -1.58721 | -1.57695 |
| H | -5.06619 | -2.02535 | -0.36604 |
| H | -3.26949 | 1.64233 | 0.34000 |
| H | -2.20444 | -0.36535 | -1.75421 |
| H | -1.78011 | 1.34044 | -1.61725 |
| H | -0.85633 | 1.13167 | 1.79761 |
| H | 1.46467 | 0.50631 | 1.76806 |
| H | 1.64392 | 2.07618 | 0.97785 |
| H | 0.41538 | -0.78075 | -1.84930 |
| H | 2.96934 | -0.30696 | -1.82585 |
| H | 3.27741 | 1.31131 | -1.18100 |
| H | 5.67139 | -0.29182 | 1.89809 |
| H | 5.56849 | -1.87509 | 1.12745 |
| H | 6.93752 | -0.79294 | 0.73633 |
| H | -0.51958 | 3.18175 | 0.84337 |
| H | -1.63104 | -2.96086 | 0.48481 |

**5k**

| C | -4.73273 | -2.32573 | -0.76209 |
| --- | --- | --- | --- |
| C | -4.13489 | -0.96462 | -0.49328 |
| C | -4.98914 | -0.06608 | 0.36819 |
| C | -2.94338 | -0.62868 | -1.01431 |
| C | -2.21019 | 0.68057 | -0.88331 |
| C | -0.90519 | 0.59770 | -0.03390 |
| C | -0.17261 | 1.99324 | 0.07360 |
| C | 1.31944 | 1.61650 | 0.21646 |
| C | 1.40288 | 0.27517 | -0.48666 |
| C | 0.18989 | -0.27923 | -0.61016 |
| C | 2.69348 | -0.31688 | -0.94956 |
| O | 3.57932 | -0.40310 | 0.19527 |
| C | 4.84992 | -0.78591 | -0.07952 |
| C | 5.67853 | -0.83952 | 1.18276 |
| O | 5.24256 | -1.04776 | -1.19486 |
| O | -0.38677 | 2.80808 | -1.06944 |
| C | -1.33080 | 0.16304 | 1.37297 |
| O | -1.99789 | 0.84455 | 2.12294 |
| O | -0.91242 | -1.07861 | 1.70894 |
| H | -4.90726 | -2.87297 | 0.17491 |
| H | -5.71150 | -2.23562 | -1.25409 |
| H | -4.08832 | -2.93932 | -1.39956 |
| H | -5.26407 | -0.58014 | 1.29900 |
| H | -4.49763 | 0.86818 | 0.64450 |
| H | -5.93226 | 0.17570 | -0.14202 |
| H | -2.43686 | -1.38193 | -1.61974 |
| H | -1.92969 | 1.04307 | -1.87881 |
| H | -2.84222 | 1.45529 | -0.44282 |
| H | -0.56359 | 2.57107 | 0.91208 |
| H | 1.61783 | 1.51387 | 1.26917 |
| H | 1.95982 | 2.39288 | -0.21998 |
| H | -0.00275 | -1.25953 | -1.03460 |
| H | 2.55586 | -1.31507 | -1.37675 |
| H | 3.17982 | 0.30666 | -1.70970 |
| H | 5.67827 | 0.13722 | 1.67734 |
| H | 5.24688 | -1.56084 | 1.88452 |
| H | 6.69950 | -1.13063 | 0.93401 |
| H | 0.02406 | 2.35843 | -1.82694 |
| H | -1.27547 | -1.24910 | 2.59940 |

**5l**

| C | 4.53494 | 2.66142 | 0.12631 |
| --- | --- | --- | --- |
| C | 3.92087 | 1.36142 | -0.33675 |
| C | 4.91597 | 0.24936 | -0.56566 |
| C | 2.59470 | 1.26014 | -0.52174 |
| C | 1.81137 | 0.06359 | -0.98984 |
| C | 0.83584 | -0.51070 | 0.09338 |
| C | 0.00012 | -1.72206 | -0.47383 |
| C | -1.24846 | -1.06350 | -1.08178 |
| C | -1.39986 | 0.18250 | -0.23213 |
| C | -0.27225 | 0.46160 | 0.43401 |
| C | -2.64701 | 1.00328 | -0.21708 |
| O | -3.74874 | 0.14350 | 0.17171 |
| C | -4.98025 | 0.70245 | 0.09431 |
| C | -6.04339 | -0.28118 | 0.52537 |
| O | -5.17704 | 1.84059 | -0.27011 |
| O | 0.66666 | -2.53568 | -1.41398 |
| C | 1.63888 | -0.93846 | 1.30590 |
| O | 1.62003 | -0.45053 | 2.41116 |
| O | 2.43102 | -2.01637 | 1.00801 |
| H | 5.07128 | 2.52842 | 1.07624 |
| H | 5.27433 | 3.02920 | -0.59918 |
| H | 3.78171 | 3.44244 | 0.26865 |
| H | 5.52364 | 0.08910 | 0.33561 |
| H | 4.45086 | -0.70322 | -0.82711 |
| H | 5.61865 | 0.51689 | -1.36724 |
| H | 1.99444 | 2.14663 | -0.31339 |
| H | 1.19320 | 0.34559 | -1.85194 |
| H | 2.46270 | -0.74284 | -1.33132 |
| H | -0.32317 | -2.32375 | 0.39028 |
| H | -1.07177 | -0.81809 | -2.13921 |
| H | -2.12495 | -1.71816 | -1.04363 |
| H | -0.12859 | 1.29505 | 1.11376 |
| H | -2.57480 | 1.83801 | 0.48696 |
| H | -2.87198 | 1.42103 | -1.20588 |
| H | -5.99707 | -1.18396 | -0.09226 |
| H | -5.87304 | -0.58649 | 1.56312 |
| H | -7.02566 | 0.18320 | 0.43299 |
| H | 1.40704 | -2.95402 | -0.94359 |
| H | 2.90889 | -2.23639 | 1.83007 |

## 3.6 ECD calculation of 6

Conformation search of **6** at MMFF94s force field gave eight conformers **6a**-**6h**. These conformers were optimized at B3LYP/6-31G(d) level, and then calculated the ECD at B3LYP/6-31G(d,p) level.

| Conformers | Gibbs free energies | Population (%) |
| --- | --- | --- |
| **6a** | -1149.212786 | 16.64 |
| **6b** | -1149.211643 | 4.96 |
| **6c** | -1149.214008 | 60.73 |
| **6d** | -1149.212055 | 7.67 |
| **6e** | -1149.210679 | 1.79 |
| **6f** | -1149.210186 | 1.06 |
| **6g** | -1149.211470 | 4.13 |
| **6h** | -1149.211174 | 3.02 |

Standard orientation of optimized **6** at B3LYP/6-31G(d) level.

**6a**

| C | 1.20534 | 1.82104 | -0.97449 |
| --- | --- | --- | --- |
| C | 1.21220 | 0.79482 | 0.21012 |
| C | -0.28184 | 2.23255 | -1.11227 |
| C | -0.83853 | 1.99809 | 0.27941 |
| C | -0.00323 | 1.25138 | 1.01604 |
| C | 2.53250 | 0.71695 | 1.01591 |
| C | 3.71887 | 0.30766 | 0.18169 |
| C | 4.42428 | -0.83295 | 0.24749 |
| C | 5.59984 | -1.05828 | -0.67456 |
| C | 4.15702 | -1.96913 | 1.20485 |
| C | -2.21874 | 2.41943 | 0.69324 |
| O | -3.20130 | 1.56027 | 0.03796 |
| C | -3.44578 | 0.37637 | 0.64456 |
| C | -4.33681 | -0.52069 | -0.20055 |
| O | -2.99826 | 0.06020 | 1.72662 |
| C | -3.87670 | -1.98971 | -0.11347 |
| O | 2.03194 | 2.94718 | -0.70205 |
| C | 0.78497 | -0.58128 | -0.30719 |
| O | 0.46141 | -0.80204 | -1.47011 |
| O | 0.68078 | -1.49083 | 0.66217 |
| C | -2.43037 | -2.16867 | -0.53711 |
| O | -1.62201 | -2.85963 | 0.06552 |
| O | -2.13207 | -1.46375 | -1.63195 |
| H | 1.60502 | 1.38086 | -1.88748 |
| H | -0.80557 | 1.60857 | -1.84703 |
| H | -0.36549 | 3.27332 | -1.45039 |
| H | -0.20383 | 0.88973 | 2.01924 |
| H | 2.37711 | 0.04210 | 1.86174 |
| H | 2.71802 | 1.71359 | 1.43525 |
| H | 4.01984 | 1.05036 | -0.55631 |
| H | 5.76458 | -0.20771 | -1.34315 |
| H | 6.52337 | -1.22641 | -0.10251 |
| H | 5.45068 | -1.95485 | -1.29252 |
| H | 3.26735 | -1.82183 | 1.81967 |
| H | 4.02493 | -2.91001 | 0.65332 |
| H | 5.01661 | -2.12093 | 1.87287 |
| H | -2.35352 | 2.35575 | 1.77440 |
| H | -2.47025 | 3.42310 | 0.34177 |
| H | -5.36097 | -0.44284 | 0.18559 |
| H | -4.33981 | -0.16665 | -1.23256 |
| H | -3.96368 | -2.36762 | 0.90630 |
| H | -4.51112 | -2.60421 | -0.76419 |
| H | 1.66605 | 3.39360 | 0.07976 |
| H | 0.10613 | -2.23215 | 0.34159 |
| H | -1.14749 | -1.47253 | -1.76265 |

**6b**

| C | 1.20128 | 1.81669 | -0.96744 |
| --- | --- | --- | --- |
| C | 1.21087 | 0.79327 | 0.20773 |
| C | -0.27806 | 2.27112 | -1.07673 |
| C | -0.83799 | 1.99547 | 0.30380 |
| C | -0.00598 | 1.23066 | 1.02021 |
| C | 2.53001 | 0.71597 | 1.01709 |
| C | 3.71954 | 0.30192 | 0.18985 |
| C | 4.42332 | -0.83957 | 0.25853 |
| C | 5.60598 | -1.06536 | -0.65448 |
| C | 4.14787 | -1.97658 | 1.21265 |
| C | -2.21486 | 2.41208 | 0.72877 |
| O | -3.20442 | 1.56497 | 0.06364 |
| C | -3.45216 | 0.37523 | 0.65511 |
| C | -4.34313 | -0.51101 | -0.20207 |
| O | -3.00812 | 0.04340 | 1.73408 |
| C | -3.88465 | -1.98148 | -0.13044 |
| O | 2.04854 | 2.90202 | -0.58897 |
| C | 0.78466 | -0.57926 | -0.32228 |
| O | 0.45166 | -0.79029 | -1.48493 |
| O | 0.68656 | -1.49724 | 0.63902 |
| C | -2.43826 | -2.15685 | -0.55534 |
| O | -1.62868 | -2.84953 | 0.04379 |
| O | -2.14122 | -1.44705 | -1.64736 |
| H | 1.55816 | 1.35446 | -1.89330 |
| H | -0.81905 | 1.69642 | -1.83952 |
| H | -0.34573 | 3.33099 | -1.35920 |
| H | -0.20499 | 0.84198 | 2.01315 |
| H | 2.36854 | 0.03911 | 1.86021 |
| H | 2.70661 | 1.71491 | 1.43001 |
| H | 4.02890 | 1.04502 | -0.54453 |
| H | 5.77704 | -0.21412 | -1.32095 |
| H | 6.52522 | -1.23460 | -0.07577 |
| H | 5.46143 | -1.96119 | -1.27476 |
| H | 3.25221 | -1.82933 | 1.81856 |
| H | 4.02153 | -2.91752 | 0.65974 |
| H | 5.00089 | -2.12807 | 1.88923 |
| H | -2.34577 | 2.33066 | 1.80896 |
| H | -2.46677 | 3.42192 | 0.39577 |
| H | -5.36803 | -0.43666 | 0.18277 |
| H | -4.34360 | -0.14569 | -1.23018 |
| H | -3.97227 | -2.36960 | 0.88541 |
| H | -4.51929 | -2.58848 | -0.78799 |
| H | 2.09111 | 3.51011 | -1.34239 |
| H | 0.10488 | -2.23217 | 0.31683 |
| H | -1.15617 | -1.45213 | -1.77597 |

**6c**

| C | -1.43367 | 0.95380 | 1.12638 |
| --- | --- | --- | --- |
| C | -1.16793 | 0.14496 | -0.19319 |
| C | -0.15300 | 1.78874 | 1.33962 |
| C | 0.48564 | 1.84215 | -0.03203 |
| C | -0.09700 | 0.98595 | -0.87951 |
| C | -2.42014 | -0.10148 | -1.06833 |
| C | -3.51671 | -0.84354 | -0.34642 |
| C | -4.82085 | -0.52614 | -0.25855 |
| C | -5.78073 | -1.41898 | 0.49307 |
| C | -5.46413 | 0.68038 | -0.90200 |
| C | 1.71068 | 2.65422 | -0.33560 |
| O | 2.88520 | 1.98635 | 0.22522 |
| C | 3.42663 | 1.01039 | -0.53595 |
| C | 4.51867 | 0.25241 | 0.20377 |
| O | 3.07850 | 0.75510 | -1.66987 |
| C | 4.45046 | -1.25534 | -0.11243 |
| O | -2.51508 | 1.86595 | 0.97400 |
| C | -0.41487 | -1.14513 | 0.14010 |
| O | -0.02751 | -1.43316 | 1.26909 |
| O | -0.11222 | -1.86925 | -0.93811 |
| C | 3.10225 | -1.85812 | 0.23717 |
| O | 2.48702 | -2.62577 | -0.48873 |
| O | 2.65035 | -1.43489 | 1.42063 |
| H | -1.61834 | 0.27400 | 1.96442 |
| H | 0.51842 | 1.31212 | 2.06499 |
| H | -0.41335 | 2.77961 | 1.73085 |
| H | 0.21409 | 0.80134 | -1.90236 |
| H | -2.10388 | -0.67754 | -1.94797 |
| H | -2.76297 | 0.87018 | -1.43091 |
| H | -3.19725 | -1.76332 | 0.14779 |
| H | -5.27562 | -2.28237 | 0.93684 |
| H | -6.28808 | -0.86845 | 1.29739 |
| H | -6.57124 | -1.79260 | -0.17265 |
| H | -4.74798 | 1.36350 | -1.36411 |
| H | -6.17268 | 0.36375 | -1.67991 |
| H | -6.04943 | 1.24889 | -0.16657 |
| H | 1.85506 | 2.78209 | -1.40956 |
| H | 1.69858 | 3.62757 | 0.16018 |
| H | 5.48852 | 0.64377 | -0.12831 |
| H | 4.43160 | 0.43668 | 1.27563 |
| H | 4.62250 | -1.44066 | -1.17372 |
| H | 5.22677 | -1.77807 | 0.46001 |
| H | -3.32545 | 1.33105 | 0.93954 |
| H | 0.64828 | -2.46448 | -0.71370 |
| H | 1.70064 | -1.70730 | 1.51791 |

**6d**

| C | 1.45491 | 1.20638 | -0.97408 |
| --- | --- | --- | --- |
| C | 1.21353 | 0.33715 | 0.30695 |
| C | 0.07043 | 1.83699 | -1.26596 |
| C | -0.57469 | 1.89290 | 0.10719 |
| C | 0.07798 | 1.11044 | 0.97947 |
| C | 2.45922 | 0.09410 | 1.19540 |
| C | 3.43854 | -0.89716 | 0.61813 |
| C | 4.68760 | -0.67904 | 0.17796 |
| C | 5.53205 | -1.82675 | -0.32411 |
| C | 5.37397 | 0.66530 | 0.15196 |
| C | -1.86394 | 2.61121 | 0.38148 |
| O | -2.96640 | 1.87627 | -0.23238 |
| C | -3.46275 | 0.84642 | 0.49052 |
| C | -4.47834 | 0.03645 | -0.30028 |
| O | -3.12927 | 0.58956 | 1.62795 |
| C | -4.31349 | -1.47139 | -0.02266 |
| O | 2.42859 | 2.22066 | -0.74607 |
| C | 0.53400 | -0.97721 | -0.08687 |
| O | 0.21358 | -1.26256 | -1.23665 |
| O | 0.22548 | -1.73511 | 0.96561 |
| C | -2.91327 | -1.96525 | -0.33630 |
| O | -2.27220 | -2.71019 | 0.39050 |
| O | -2.44756 | -1.47108 | -1.48673 |
| H | 1.83878 | 0.61078 | -1.80159 |
| H | -0.52273 | 1.21412 | -1.94630 |
| H | 0.18490 | 2.82068 | -1.73870 |
| H | -0.22435 | 0.92382 | 2.00478 |
| H | 2.10277 | -0.28673 | 2.16149 |
| H | 2.93142 | 1.05967 | 1.38766 |
| H | 3.06985 | -1.92372 | 0.58583 |
| H | 5.00513 | -2.78404 | -0.26101 |
| H | 5.83041 | -1.66843 | -1.36987 |
| H | 6.46325 | -1.91238 | 0.25386 |
| H | 4.68452 | 1.49857 | 0.29294 |
| H | 6.15936 | 0.71710 | 0.91981 |
| H | 5.87417 | 0.81606 | -0.81381 |
| H | -2.05205 | 2.70841 | 1.45213 |
| H | -1.90285 | 3.59274 | -0.09747 |
| H | -5.48171 | 0.35054 | 0.01368 |
| H | -4.37498 | 0.25711 | -1.36377 |
| H | -4.50980 | -1.70050 | 1.02581 |
| H | -5.02921 | -2.03037 | -0.63808 |
| H | 2.07220 | 2.80983 | -0.05989 |
| H | -0.47387 | -2.38442 | 0.69640 |
| H | -1.47741 | -1.67232 | -1.55440 |

**6e**

| C | -1.29938 | 1.90589 | 0.87661 |
| --- | --- | --- | --- |
| C | -1.21111 | 0.77938 | -0.21455 |
| C | 0.15597 | 2.41317 | 1.03561 |
| C | 0.79920 | 2.05690 | -0.28931 |
| C | 0.03141 | 1.21298 | -0.99417 |
| C | -2.48090 | 0.58719 | -1.07640 |
| C | -3.70775 | 0.24677 | -0.26923 |
| C | -4.38592 | -0.91187 | -0.24165 |
| C | -5.61519 | -1.05562 | 0.62534 |
| C | -4.03644 | -2.14551 | -1.03812 |
| C | 2.19752 | 2.46603 | -0.65386 |
| O | 3.13634 | 1.60932 | 0.06883 |
| C | 3.44406 | 0.43653 | -0.53134 |
| C | 4.25080 | -0.47059 | 0.38684 |
| O | 3.10906 | 0.13901 | -1.65797 |
| C | 3.91791 | -1.95610 | 0.13707 |
| O | -2.16916 | 2.95793 | 0.47062 |
| C | -0.75388 | -0.56645 | 0.36061 |
| O | -0.71012 | -1.59242 | -0.31022 |
| O | -0.28338 | -0.49102 | 1.60580 |
| C | 2.45726 | -2.21131 | 0.45725 |
| O | 1.97037 | -1.93318 | 1.54937 |
| O | 1.76354 | -2.69974 | -0.56240 |
| H | -1.71964 | 1.53591 | 1.81164 |
| H | 0.67087 | 1.91169 | 1.86342 |
| H | 0.16537 | 3.48994 | 1.24937 |
| H | 0.30047 | 0.76393 | -1.94525 |
| H | -2.26234 | -0.17842 | -1.82495 |
| H | -2.65989 | 1.52279 | -1.62042 |
| H | -4.06932 | 1.06353 | 0.35428 |
| H | -5.84008 | -0.13537 | 1.17344 |
| H | -6.49582 | -1.31605 | 0.02101 |
| H | -5.49070 | -1.86734 | 1.35592 |
| H | -3.09286 | -2.06006 | -1.57905 |
| H | -3.95549 | -3.01685 | -0.37417 |
| H | -4.83322 | -2.37844 | -1.75890 |
| H | 2.38473 | 2.37424 | -1.72475 |
| H | 2.43975 | 3.47557 | -0.31405 |
| H | 5.31622 | -0.29695 | 0.19057 |
| H | 4.05666 | -0.20182 | 1.42744 |
| H | 4.12002 | -2.22829 | -0.89971 |
| H | 4.53423 | -2.57424 | 0.79888 |
| H | -1.79730 | 3.34680 | -0.33845 |
| H | 0.31945 | -1.26482 | 1.76167 |
| H | 0.79451 | -2.60930 | -0.36437 |

**6f**

| C | -1.24967 | -0.35379 | 2.17361 |
| --- | --- | --- | --- |
| C | -1.25654 | -0.75915 | 0.66083 |
| C | -0.16803 | 0.75229 | 2.26534 |
| C | -0.13955 | 1.32815 | 0.86258 |
| C | -0.77724 | 0.52946 | -0.00524 |
| C | -2.59079 | -1.35902 | 0.12291 |
| C | -3.71570 | -0.37546 | -0.09216 |
| C | -4.09085 | 0.19025 | -1.25165 |
| C | -5.26897 | 1.13455 | -1.30050 |
| C | -3.42127 | -0.04263 | -2.58455 |
| C | 0.64041 | 2.55695 | 0.49714 |
| O | 2.06893 | 2.25187 | 0.53018 |
| C | 2.57333 | 1.67209 | -0.58198 |
| C | 4.01444 | 1.22696 | -0.38518 |
| O | 1.95002 | 1.51168 | -1.61012 |
| C | 4.27002 | -0.12632 | -1.07865 |
| O | -2.51685 | 0.12248 | 2.60836 |
| C | -0.09062 | -1.72131 | 0.40014 |
| O | 0.69797 | -2.08972 | 1.26587 |
| O | 0.04733 | -2.03455 | -0.88859 |
| C | 3.32531 | -1.20960 | -0.59237 |
| O | 2.74827 | -1.99542 | -1.32999 |
| O | 3.17265 | -1.19112 | 0.73458 |
| H | -1.02770 | -1.20994 | 2.81134 |
| H | 0.81118 | 0.33638 | 2.53325 |
| H | -0.43183 | 1.48711 | 3.03665 |
| H | -0.83795 | 0.69011 | -1.07550 |
| H | -2.91841 | -2.12251 | 0.83977 |
| H | -2.36625 | -1.88185 | -0.80998 |
| H | -4.29209 | -0.13112 | 0.79791 |
| H | -5.72543 | 1.27124 | -0.31506 |
| H | -4.97089 | 2.12232 | -1.67963 |
| H | -6.04401 | 0.76222 | -1.98511 |
| H | -2.57633 | -0.73318 | -2.53484 |
| H | -4.14111 | -0.44021 | -3.31348 |
| H | -3.05363 | 0.90494 | -3.00263 |
| H | 0.37674 | 2.92222 | -0.49691 |
| H | 0.52586 | 3.35632 | 1.23348 |
| H | 4.66920 | 1.98674 | -0.83019 |
| H | 4.23881 | 1.17440 | 0.68129 |
| H | 4.13867 | -0.04434 | -2.15859 |
| H | 5.30100 | -0.44359 | -0.87904 |
| H | -2.73782 | 0.89384 | 2.06034 |
| H | 0.96821 | -2.37153 | -1.03866 |
| H | 2.42149 | -1.79176 | 0.98170 |

**6g**

| C | -1.40140 | -0.11396 | 1.65643 |
| --- | --- | --- | --- |
| C | -1.15795 | -0.62037 | 0.19079 |
| C | -0.35796 | 1.01924 | 1.85324 |
| C | -0.00945 | 1.44846 | 0.44441 |
| C | -0.48438 | 0.58534 | -0.46336 |
| C | -2.41853 | -1.16155 | -0.54223 |
| C | -3.34527 | -0.09275 | -1.06971 |
| C | -4.64346 | 0.10764 | -0.78075 |
| C | -5.43081 | 1.18216 | -1.49408 |
| C | -5.43745 | -0.69734 | 0.21980 |
| C | 0.88770 | 2.61280 | 0.13795 |
| O | 2.27111 | 2.25096 | 0.44039 |
| C | 2.92900 | 1.57820 | -0.52976 |
| C | 4.29043 | 1.08994 | -0.05907 |
| O | 2.48487 | 1.37395 | -1.64013 |
| C | 4.58921 | -0.31830 | -0.61172 |
| O | -2.73025 | 0.33462 | 1.87047 |
| C | -0.02615 | -1.65514 | 0.20431 |
| O | 0.59408 | -1.97132 | 1.21507 |
| O | 0.29963 | -2.08876 | -1.01337 |
| C | 3.52569 | -1.32773 | -0.22083 |
| O | 3.03817 | -2.14105 | -0.99256 |
| O | 3.16156 | -1.20951 | 1.05867 |
| H | -1.25134 | -0.92252 | 2.37270 |
| H | 0.53594 | 0.65391 | 2.37551 |
| H | -0.78406 | 1.82620 | 2.46248 |
| H | -0.30260 | 0.63950 | -1.53148 |
| H | -2.94016 | -1.85154 | 0.12683 |
| H | -2.07234 | -1.75358 | -1.39876 |
| H | -2.89962 | 0.55696 | -1.82410 |
| H | -4.81770 | 1.73167 | -2.21537 |
| H | -6.28257 | 0.74730 | -2.03561 |
| H | -5.85419 | 1.90369 | -0.78170 |
| H | -4.81117 | -1.31421 | 0.86575 |
| H | -6.02309 | -0.03168 | 0.86695 |
| H | -6.15915 | -1.34796 | -0.29386 |
| H | 0.81998 | 2.91147 | -0.90948 |
| H | 0.69133 | 3.46997 | 0.78633 |
| H | 5.04967 | 1.78915 | -0.43148 |
| H | 4.32780 | 1.10389 | 1.03127 |
| H | 4.64108 | -0.30856 | -1.70143 |
| H | 5.55663 | -0.65918 | -0.22266 |
| H | -3.00038 | 0.86762 | 1.10343 |
| H | 1.21736 | -2.46351 | -0.98108 |
| H | 2.34980 | -1.75954 | 1.21487 |

**6h**

| C | 1.45369 | 1.14704 | -0.99021 |
| --- | --- | --- | --- |
| C | 1.20569 | 0.29715 | 0.29111 |
| C | 0.09473 | 1.84639 | -1.25805 |
| C | -0.55808 | 1.87975 | 0.10936 |
| C | 0.07660 | 1.07312 | 0.96811 |
| C | 2.44798 | 0.05003 | 1.18322 |
| C | 3.45189 | -0.89911 | 0.58014 |
| C | 4.71422 | -0.64944 | 0.19848 |
| C | 5.58264 | -1.75819 | -0.34870 |
| C | 5.39366 | 0.69554 | 0.28539 |
| C | -1.83324 | 2.61662 | 0.39329 |
| O | -2.95355 | 1.90335 | -0.21921 |
| C | -3.46594 | 0.88218 | 0.50284 |
| C | -4.49360 | 0.08686 | -0.28804 |
| O | -3.13849 | 0.61826 | 1.64061 |
| C | -4.34875 | -1.42335 | -0.01126 |
| O | 2.46036 | 2.10934 | -0.67348 |
| C | 0.51125 | -1.01041 | -0.10251 |
| O | 0.17140 | -1.28601 | -1.24977 |
| O | 0.20683 | -1.77034 | 0.94897 |
| C | -2.95653 | -1.93620 | -0.33014 |
| O | -2.32030 | -2.68506 | 0.39710 |
| O | -2.49177 | -1.45483 | -1.48622 |
| H | 1.77138 | 0.51682 | -1.82662 |
| H | -0.51743 | 1.27825 | -1.96996 |
| H | 0.24441 | 2.84875 | -1.68233 |
| H | -0.23042 | 0.87011 | 1.98849 |
| H | 2.08552 | -0.37552 | 2.12829 |
| H | 2.88907 | 1.02043 | 1.41386 |
| H | 3.09240 | -1.92402 | 0.46982 |
| H | 5.05608 | -2.71740 | -0.37581 |
| H | 5.92416 | -1.52704 | -1.36766 |
| H | 6.48954 | -1.88562 | 0.25949 |
| H | 4.70408 | 1.50740 | 0.51936 |
| H | 6.19151 | 0.67962 | 1.04174 |
| H | 5.88046 | 0.93724 | -0.66964 |
| H | -2.01504 | 2.71015 | 1.46510 |
| H | -1.85910 | 3.60190 | -0.07870 |
| H | -5.49246 | 0.41387 | 0.02711 |
| H | -4.38822 | 0.30635 | -1.35159 |
| H | -4.54431 | -1.64933 | 1.03800 |
| H | -5.07416 | -1.97240 | -0.62430 |
| H | 2.69384 | 2.56273 | -1.49724 |
| H | -0.51079 | -2.40202 | 0.68627 |
| H | -1.52360 | -1.66568 | -1.55573 |

## 3.7 ECD calculation of 7

Conformation search of **7** at MMFF94s force field gave four conformers **7a**-**7d**. These conformers were optimized at B3LYP/6-31G(d) level, and then calculated the ECD at B3LYP/6-31G(d,p) level.

| Conformers | Gibbs free energies | Population (%) |
| --- | --- | --- |
| **7a** | -769.933090 | 3.94 |
| **7b** | -769.935824 | 71.33 |
| **7c** | -769.931907 | 1.13 |
| **7d** | -769.934780 | 23.61 |

Standard orientation of optimized **7** at B3LYP/6-31G(d) level.

**7a**

| C | -0.21043 | 0.55127 | -0.37537 |
| --- | --- | --- | --- |
| C | -0.43061 | -0.25070 | 0.94339 |
| C | -0.93395 | -1.61591 | 0.45343 |
| C | -1.81396 | -1.31333 | -0.79760 |
| C | -1.46193 | 0.14929 | -1.21198 |
| C | 1.08442 | 0.07366 | -1.09764 |
| C | 2.34716 | 0.28818 | -0.30610 |
| C | 3.27211 | -0.61298 | 0.06296 |
| C | 4.49682 | -0.18332 | 0.83700 |
| C | 3.22463 | -2.08970 | -0.24925 |
| C | -3.30667 | -1.49689 | -0.53121 |
| O | -3.68524 | -0.62247 | 0.54287 |
| O | -1.40447 | 0.35514 | 1.81892 |
| C | -0.15072 | 2.09535 | -0.25388 |
| O | 0.41708 | 2.77537 | -1.08228 |
| O | -0.80729 | 2.65736 | 0.76956 |
| H | 0.48793 | -0.31925 | 1.53055 |
| H | -1.48017 | -2.13345 | 1.24926 |
| H | -0.08575 | -2.25105 | 0.18260 |
| H | -1.56024 | -2.00749 | -1.60719 |
| H | -1.26858 | 0.24779 | -2.28428 |
| H | -2.29888 | 0.81410 | -0.97231 |
| H | 0.97089 | -0.97298 | -1.39538 |
| H | 1.14552 | 0.66390 | -2.01931 |
| H | 2.52314 | 1.32791 | -0.03029 |
| H | 4.49138 | 0.89087 | 1.04530 |
| H | 4.56817 | -0.71683 | 1.79556 |
| H | 5.41712 | -0.41764 | 0.28318 |
| H | 2.33663 | -2.38844 | -0.81200 |
| H | 4.10453 | -2.38830 | -0.83616 |
| H | 3.25528 | -2.68423 | 0.67464 |
| H | -3.51549 | -2.54207 | -0.26046 |
| H | -3.88707 | -1.25223 | -1.43243 |
| H | -4.61042 | -0.77890 | 0.78134 |
| H | -2.29785 | 0.08051 | 1.51645 |
| H | -1.15813 | 1.93502 | 1.35585 |

**7b**

| C | -0.24000 | 0.44673 | -0.34736 |
| --- | --- | --- | --- |
| C | -0.44135 | -0.41376 | 0.93565 |
| C | -1.02633 | -1.71880 | 0.39533 |
| C | -1.96726 | -1.31019 | -0.77523 |
| C | -1.50286 | 0.11393 | -1.20589 |
| C | 1.05857 | 0.02076 | -1.10740 |
| C | 2.34018 | 0.29170 | -0.36430 |
| C | 3.29716 | -0.57601 | 0.00277 |
| C | 4.53316 | -0.09549 | 0.72649 |
| C | 3.27027 | -2.06237 | -0.25872 |
| C | -3.46269 | -1.32950 | -0.39514 |
| O | -3.85743 | -0.28916 | 0.47598 |
| O | -1.41782 | 0.13095 | 1.82745 |
| C | -0.17664 | 1.93473 | -0.04703 |
| O | -0.32125 | 2.46395 | 1.04350 |
| O | 0.05274 | 2.67744 | -1.15131 |
| H | 0.50620 | -0.55545 | 1.47051 |
| H | -1.54562 | -2.25866 | 1.19446 |
| H | -0.22256 | -2.37185 | 0.03939 |
| H | -1.84396 | -2.02052 | -1.60213 |
| H | -1.27570 | 0.18447 | -2.27380 |
| H | -2.30562 | 0.82470 | -0.98835 |
| H | 0.96562 | -1.03581 | -1.37172 |
| H | 1.06876 | 0.57474 | -2.05466 |
| H | 2.50623 | 1.34187 | -0.11680 |
| H | 4.51292 | 0.98493 | 0.89936 |
| H | 4.63945 | -0.59442 | 1.69981 |
| H | 5.44197 | -0.33242 | 0.15566 |
| H | 2.37001 | -2.39936 | -0.77780 |
| H | 4.13659 | -2.36228 | -0.86442 |
| H | 3.34420 | -2.62144 | 0.68410 |
| H | -3.70073 | -2.32221 | 0.02709 |
| H | -4.06961 | -1.21406 | -1.30220 |
| H | -3.15148 | -0.17115 | 1.14549 |
| H | -1.16311 | 1.06322 | 1.97680 |
| H | 0.04332 | 3.60976 | -0.86079 |

**7c**

| C | -0.16782 | 0.20917 | -0.25610 |
| --- | --- | --- | --- |
| C | -0.75274 | -0.53590 | 0.98195 |
| C | -1.62337 | -1.63550 | 0.35856 |
| C | -2.24694 | -0.99902 | -0.92123 |
| C | -1.40430 | 0.28361 | -1.20259 |
| C | 0.95815 | -0.63067 | -0.93815 |
| C | 2.07629 | -1.09278 | -0.03709 |
| C | 3.34475 | -0.65228 | 0.00829 |
| C | 4.33600 | -1.27968 | 0.96062 |
| C | 3.91368 | 0.44975 | -0.85123 |
| C | -3.73500 | -0.69115 | -0.76738 |
| O | -3.89829 | 0.21558 | 0.33384 |
| O | -1.54782 | 0.31511 | 1.83230 |
| C | 0.37156 | 1.64262 | -0.02521 |
| O | 1.10333 | 2.17995 | -0.83056 |
| O | -0.05511 | 2.30020 | 1.05997 |
| H | 0.03143 | -0.92683 | 1.63286 |
| H | -2.37340 | -1.98735 | 1.07482 |
| H | -1.00263 | -2.49686 | 0.09340 |
| H | -2.16466 | -1.69854 | -1.76107 |
| H | -1.09435 | 0.36328 | -2.24858 |
| H | -1.99817 | 1.17521 | -0.97400 |
| H | 0.49606 | -1.51519 | -1.39861 |
| H | 1.34098 | -0.01927 | -1.75735 |
| H | 1.82020 | -1.91163 | 0.63765 |
| H | 3.89121 | -2.09179 | 1.54490 |
| H | 5.20101 | -1.68535 | 0.41698 |
| H | 4.73416 | -0.53277 | 1.66139 |
| H | 3.15447 | 0.99365 | -1.41349 |
| H | 4.44153 | 1.18168 | -0.22546 |
| H | 4.65963 | 0.04437 | -1.55025 |
| H | -4.29473 | -1.61909 | -0.58050 |
| H | -4.12544 | -0.23303 | -1.68751 |
| H | -4.84070 | 0.36058 | 0.50142 |
| H | -2.44371 | 0.39287 | 1.43681 |
| H | -0.67129 | 1.71029 | 1.56952 |

**7d**

| C | -0.22039 | 0.04970 | -0.18299 |
| --- | --- | --- | --- |
| C | -0.89584 | -0.66759 | 1.02586 |
| C | -1.83036 | -1.67784 | 0.36124 |
| C | -2.39064 | -0.96793 | -0.90487 |
| C | -1.38975 | 0.18617 | -1.21143 |
| C | 0.93864 | -0.80983 | -0.78812 |
| C | 2.12474 | -1.01731 | 0.11851 |
| C | 3.37987 | -0.56604 | -0.04297 |
| C | 4.44882 | -0.89766 | 0.97181 |
| C | 3.86378 | 0.26951 | -1.20324 |
| C | -3.83019 | -0.44107 | -0.72739 |
| O | -3.94149 | 0.68277 | 0.12230 |
| O | -1.72093 | 0.20813 | 1.79778 |
| C | 0.33378 | 1.41341 | 0.19148 |
| O | 0.28507 | 1.94070 | 1.29089 |
| O | 0.90340 | 2.04605 | -0.85664 |
| H | -0.15757 | -1.13948 | 1.68627 |
| H | -2.61291 | -1.98722 | 1.06238 |
| H | -1.27265 | -2.57950 | 0.08639 |
| H | -2.41825 | -1.68339 | -1.73593 |
| H | -1.00984 | 0.15907 | -2.23694 |
| H | -1.90359 | 1.14189 | -1.07293 |
| H | 0.51471 | -1.78627 | -1.05842 |
| H | 1.24074 | -0.34050 | -1.72715 |
| H | 1.93141 | -1.62107 | 1.00537 |
| H | 4.06223 | -1.51384 | 1.78939 |
| H | 5.28320 | -1.43785 | 0.50279 |
| H | 4.87663 | 0.01632 | 1.40675 |
| H | 3.06953 | 0.56484 | -1.89078 |
| H | 4.34658 | 1.18627 | -0.83784 |
| H | 4.62919 | -0.27385 | -1.77478 |
| H | -4.46490 | -1.27529 | -0.37868 |
| H | -4.22399 | -0.12576 | -1.70202 |
| H | -3.34045 | 0.54690 | 0.88405 |
| H | -1.16115 | 0.97554 | 2.03071 |
| H | 1.20790 | 2.91084 | -0.52064 |

# Experimental section

## 4.1 General experimental procedures

Optical rotations were obtained on an Autopol IV-T digital polarimeter (Rudolph, Hackettstown, USA). UV spectra were recorded on a Hitachi UH5300 spectrophotometer (Hitachi, Tokyo, Japan). CD spectra were measured on a Chirascan Circular Dichroism Spectrometer (Applied Photophysics Limited, Leatherhead, Surrey, UK). 1D and 2D NMR spectra were obtained on a Bruker Avance III 600 MHz spectrometer (Bruker Corporation, Karlsruhe, Germany). HRESIMS data were measured on a Q Exactive Orbitrap mass spectrometer (Thermo Fisher Scientific, MA, USA). Medium pressure liquid chromatography (MPLC) was performed on an Interchim system equipping with a column packed with Chromatorex C_18_ gel (40–75 *μ*m, Fuji Silysia Chemical Ltd., Kasugai, Japan). Preparative high performance liquid chromatography (prep-HPLC) was performed on an Agilent 1260 Infinity II liquid chromatography system equipped with a Zorbax SB-C_18_ column (particle size 5 *μ*m, dimensions 9.4 mm × 150 mm or 7 *μ*m, 21.2 mm × 250 mm, flow rate 5 and 20 mL·min^-1^, respectively) and a DAD detector (Agilent Technologies, Santa Clara, CA, USA). Silica gel (200-300 mesh, Qingdao Haiyang Chemical Co., Ltd., China) and Sephadex LH-20 (GE Healthcare, Sweden) were used for column chromatography (CC).

## 4.2 Fungal material

The fungus *Stereum hirsutum* HFG27 was collected from Jianchuan County, Yunnan Province, China in 2006, and was identified by the molecular evidence of ITS gene fragment (GenBank accession No. MG655586.1). *Boreostereum vibrans* was collected from the Ailao Mountain of Yunnan Province, China in August 2005, and identified by Prof. Mu Zang of Kunming Institute of Botany, CAS. A voucher specimen (NO. BV20150901D.4) has been deposited in the School of Pharmaceutical Sciences, South-Central Minzu University.

The strains of *S. hirsutum* and *B. vibrans* were each activated on potato dextrose agar medium at 25 °C. After seven days, the agar plugs were cut into small pieces to seeding two hundred and fifty 500-mL Erlenmeyer flasks, each containing 400 mL of liquid culture medium (20% peeled-potato, 2% glucose, 0.1% peptone from porcine meat, 0.15% KH_2_PO_4_, 0.15% MgSO_4_, and 0.001% vitamin B_1_). The flasks were incubated on rotatory shakers at 25 °C and 150 rpm for 25 d in dark environment.

## 4.3 Extraction and isolation

The co-culture broth (100 L) of *S. hirsutum* and *B. vibrans* was centrifuged to separate the mycelium and liquid layer. The liquid layer was concentrated under reduced pressure to 1 liter and was further partitioned against ethyl acetate (EtOAc) (each time 2 L) and water for three times to obtain an EtOAc layer (6 L). The mycelia were soaked with 95% MeOH (total 1.5 L) at room temperature. The extract was evaporated under reduced pressure and re-dissolved in water (0.5 L) and was further partitioned against EtOAc (each time 1 L) for three times to give an EtOAc layer. The EtOAc layer was further concentrated *in vacuo* and the combining EtOAc layer (3 L) was further concentrated *in vacuo* to obtain 36.3 g of the extract. The extracts were combined and fractionated on MPLC with a stepwise gradient of MeOH-H_2_O (0-100%) to afford twelve fractions (A–L).

Fraction C was subjected to a Sephadex LH-20 column chromatography (CC) eluting with acetone and yielded six subfractions (C1–C6). Subfractions C4 were purified by prep-HPLC [(MeCN/MeOH= 2/1)-H_2_O (supplemented with 0.1% formic acid): 7–35%, 35 min, 4 mL·min^-1^) and yielded two subfractions C4-1–C4-2. Subfractions C4-1 were purified by prep-HPLC [MeOH-H_2_O (supplemented with 0.1% formic acid): 25–45%, 25 min, 4 mL·min^-1^) to afford compound **9** (t*_R_* = 22.00 min, 3.1 mg).

Fraction D was subjected to a Sephadex LH-20 column chromatography (CC) eluting with acetone and yielded seven subfractions (D1–D7). Subfraction D6 was subjected to silica gel CC using petroleum ether-acetone mixtures of increasing polarities (v/v, 10:1 to 1:1) to yield seven main subfractions D6-1–D6-7. Subfraction D6-3 was purified by prep-HPLC [MeCN-H_2_O (supplemented with 0.1% Formic acid): 15–29.4%, 18 min, 4 mL·min^-1^] to afford compound **7** (t*_R_* = 14.86 min, 3.3 mg). Subfraction F6-4 was separated by prep-HPLC [MeCN-H_2_O (supplemented with 0.1% formic acid): 15–33.4%, 23 min, 4 mL·min^-1^] to obtain compounds **8** (t*_R_* = 14.89 min, 2.1 mg) and **1** (t_R_ = 19.21 min, 7.1 mg). Subfraction D7 was purified by prep-HPLC [MeCN-H_2_O (supplemented with 0.1% formic acid): 16.0–30.6%, 18 min, 4 mL·min^-1^] to afford compound **2** (t*_R_* = 13.95 min, 14.6 mg).

Fraction E was subjected to silica gel CC eluting with petroleum ether-acetone mixtures of increasing polarities (v/v, 10:1 to 1:1) to yield four main subfractions (E1–E4). Subfraction E3 was subjected to Sephadex LH-20 column chromatography eluting with acetone and yielded four subfractions E3-1–E3-4. Subfraction E3-3 was separated by prep-HPLC [MeOH-H_2_O (supplemented with 0.1% formic acid): 32–60%, 35 min, 15 mL·min^-1^] and yielded ten subfractions E3-3-1–E3-3-10. Subfraction E3-3-6 was separated by prep-HPLC [MeOH-H_2_O (supplemented with 0.1% formic acid): 16–36%, 25 min, 4 mL·min^-1^] to obtain compound **5** (t_R_= 20.14 min, 2.4 mg). Subfraction E4 was subjected to Sephadex LH-20 column chromatography eluting with Acetone and yielded five subfractions E4-1–E4-5. Subfraction E4-2 was separated by prep-HPLC [MeOH-H_2_O (supplemented with 0.1% formic acid): 20–52%, 40 min, 15 mL·min^-1^] to yield eight subfractions E4-2-1–E4-2-8 and obtain compound **4** (t_R_= 22.43 min, 9.8 mg). Subfraction E4-3 was separated by prep-HPLC [MeOH-H_2_O (supplemented with 0.1% formic acid): 15–31%, 40 min, 15 mL·min^-1^] to obtain compounds **10** (t*_R_* = 13.45 min, 2.3 mg), **11** (t*_R_* = 26.43 min, 1.6 mg), **6** (t_R_= 30.35 min, 6.0 mg) and **3** (t_R_ = 33.42 min, 1.7 mg).

## 4.4 Characterization data

### 4.4.1 *Hirsutavibrin A (****1****)*

Colorless oil; C_12_H_16_O_5_; [*α*]^21.4^_D_ +43.6 (*c* 0.05, MeOH); UV (MeOH) λ_max_ (log *ε*) 215.0 (3.83) nm; ^1^H NMR (600 MHz, CD_3_OD) data: Table 1, ^13^C NMR (150 MHz, CD_3_OD) data: Table 3; HRESIMS *m/z* 241.10703 [M + H]^+^ (calcd for C_12_H_17_O_5_, 241.10705).

### 4.4.2 *Hirsutavibrin B (****2****)*

Colorless oil; C_12_H_16_O_5_; [*α*]^21.4^_D_ +14.4 (*c* 0.05, MeOH); UV (MeOH) λ_max_ (log *ε*) 215.0 (3.84) nm; ^1^H NMR (500 MHz, CD_3_OD) data: Table 1, ^13^C NMR (125 MHz, CD_3_OD) data: Table 3; HRESIMS *m/z* 241.10707 [M + H]^+^ (calcd for C_12_H_17_O_5_, 241.10705).

### 4.4.3 *Hirsutavibrin C (****3****)*

Colorless oil; C_14_H_18_O_6_; [*α*]^23.6^_D_ +45.8 (*c* 0.05, MeOH); UV (MeOH) λ_max_ (log *ε*) 210.0 (4.00) nm; ^1^H NMR (600 MHz, CD_3_OD) data: Table 1, ^13^C NMR (150 MHz, CD_3_OD) data: Table 3; HRESIMS *m/z* 283.11765 [M + H]^+^ (calcd for C_14_H_19_O_6_, 283.11761).

### 4.4.4 *Hirsutavibrin D (****4****)*

Colorless oil; C_14_H_20_O_5_; [*α*]^23.7^_D_ +37.8 (*c* 0.05, MeOH); UV (MeOH) λ_max_ (log *ε*) 205.0 (3.61) nm; ^1^H NMR (600 MHz, CD_3_OD) data: Table 1, ^13^C NMR (150 MHz, CD_3_OD) data: Table 3; HRESIMS *m/z* 269.13834 [M + H]^+^ (calcd for C_14_H_21_O_5_, 269.13835).

### 4.4.5 *Hirsutavibrin E (****5****)*

Colorless oil; C_14_H_20_O_5_; [*α*]^23.6^_D_ +14.7 (*c* 0.05, MeOH); UV (MeOH) λ_max_ (log *ε*) 210.0 (3.73) nm; ^1^H NMR (600 MHz, CDCl_3_) data: Table 1, ^13^C NMR (150 MHz, CDCl_3_) data: Table 3; HRESIMS *m/z* 269.13834 [M + H]^+^ (calcd for C_14_H_21_O_5_, 269.13835).

### 4.4.6 *Hirsutavibrin F (****6****)*

Colorless oil; C_16_H_22_O_7_; [*α*]^23.6^_D_ +2.0 (*c* 0.05, MeOH); UV (MeOH) λ_max_ (log *ε*) 210.0 (3.63) nm; ^1^H NMR (600 MHz, CD_3_OD) data: Table 1, ^13^C NMR (150 MHz, CD_3_OD) data: Table 3; HRESIMS *m/z* 327.14359 [M + H]^+^ (calcd for C_16_H_23_O_7_, 327.14383).

### 4.4.7 *Hirsutavibrin G (****7****)*

Colorless oil; C_12_H_20_O_4_; [*α*]^23.9^_D_ −12.4 (*c* 0.05, MeOH); UV (MeOH) λ_max_ (log *ε*) 215.0 (2.29) nm; ^1^H NMR (600 MHz, CD_3_OD) data: Table 2, ^13^C NMR (150 MHz, CD_3_OD) data: Table 3; HRESIMS *m/z* 229.14349 [M + H]^+^ (calcd for C_12_H_21_O_4_, 229.14344).

### 4.4.8 *Hirsutavibrin H (****8****)*

Colorless oil; C_12_H_20_O_4_; [*α*]^22.2^_D_ −15.6 (*c* 0.05, MeOH); UV (MeOH) λ_max_ (log *ε*) 210.0 (2.44) nm; ^1^H NMR (600 MHz, CD_3_OD) data: Table 2, ^13^C NMR (150 MHz, CD_3_OD) data: Table 3; HRESIMS *m/z* 229.14352 [M + H]^+^ (calcd for C_12_H_21_O_4_, 229.14344).

### 4.4.9 *Hirsutavibrin I (****9****)*

Colorless oil; C_12_H_18_O_5_; [*α*]^23.6^_D_ +22.4 (*c* 0.05, MeOH); UV (MeOH) λ_max_ (log *ε*) 205.0 (3.15) nm; ^1^H NMR (500 MHz, DMSO-*d*_6_) data: Table 2, ^13^C NMR (125 MHz, DMSO-*d*_6_) data: Table 3; HRESIMS *m/z* 265.10452 [M + Na]^+^ (calcd for C_12_H_18_O_5_Na, 265.10464).

### 4.4.10 *Hirsutavibrin J (****10****)*

Colorless oil; C_14_H_22_O_7_; [*α*]^23.6^_D_ −4.0 (*c* 0.05, MeOH); UV (MeOH) λ_max_ (log *ε*) 205.0 (3.52) nm; ^1^H NMR (600 MHz, DMSO-*d*_6_) data: Table 2, ^13^C NMR (150 MHz, DMSO-*d*_6_) data: Table 3; HRESIMS *m/z* 303.14383 [M + H]^+^ (calcd for C_14_H_23_O_7_, 303.14383).

### 4.4.11 *Hirsutavibrin K (****11****)*

Colorless oil; C_12_H_16_O_4_; [*α*]^23.6^_D_ +8.4 (*c* 0.05, MeOH); UV (MeOH) λ_max_ (log *ε*) 205.0 (3.26) nm; ^1^H NMR (500 MHz, CD_3_OD) data: Table 2, ^13^C NMR (125 MHz, CD_3_OD) data: Table 3; HRESIMS *m/z* 265.10461 [M + Na]^+^ (calcd for C_12_H_16_O_4_Na, 265.10464).

## 4.5 Biological Activity Assessment

The effects on nitric oxide production in RAW 264.7 macrophages of the isolates were screened by the protocol as previously described [4]. The human lung cancer cell line A549 used in this study was purchased from Conservation Genetics CAS Kunming Cell Bank. Cisplatin was used as positive control. The cytotoxicity assay procedures are the same as previously reported [4].

**References:**

1. Gaussian 16, Revision C.01, Frisch MJ., Trucks GW, Schlegel HB, Scuseria GE, Robb MA, Cheeseman JR, Scalmani G, Barone V, Petersson GA, Nakatsuji H, Li X, Caricato M, Marenich AV, Bloino J, Janesko BG, Gomperts R, Mennucci B, Hratchian HP, Ortiz JV, Izmaylov AF, Sonnenberg JL, Williams-Young D, Ding F, Lipparini F, Egidi F, Goings J, Peng B, Petrone A, Henderson T, Ranasinghe D, Zakrzewski VG, Gao J, Rega N, Zheng G, Liang W, Hada M, Ehara M, Toyota K, Fukuda R, Hasegawa J, Ishida M, Nakajima T, Honda Y, Kitao O, Nakai H, Vreven T, Throssell K, Montgomery JA Jr, Peralta JE, Ogliaro F, Bearpark MJ, Heyd JJ, Brothers EN, Kudin KN, Staroverov VN, Keith TA, Kobayashi R, Normand J, Raghavachari K, Rendell AP, Burant JC, Iyengar SS, Tomasi J, Cossi M, Millam JM, Klene M, Adamo C, Cammi R, Ochterski JW, Martin RL, Morokuma K, Farkas O, Foresman JB, Fox DJ. Gaussian, Inc., Wallingford CT, 2016.
2. Bruhn T, A. S, Y. H, G. P. SpecDis version 1.71, Berlin, Germany, 2017, https:/specdis-software.jimdo.com. 2017.
3. Bruhn T, Schaumlöffel A, Hemberger Y, Bringmann G. SpecDis: Quantifying the comparison of calculated and experimental electronic circular dichroism spectra. Chirality*.* 2013; 25: 243-9.
4. Chen HP, Zhao ZZ, Li ZH, Huang Y, Zhang SB, Tang Y, Yao JN, Chen L, Isaka M, Feng T, Liu JK. Anti-proliferative and anti-inflammatory lanostane triterpenoids from the polish edible mushroom *Macrolepiota procera*. J Agric. Food Chem*.* 2018; 66: 3146-54.
